# Curated collection of yeast transcription factor DNA binding specificity data reveals novel structural and gene regulatory insights

**DOI:** 10.1186/gb-2011-12-12-r125

**Published:** 2011-12-21

**Authors:** Raluca Gordân, Kevin F Murphy, Rachel P McCord, Cong Zhu, Anastasia Vedenko, Martha L Bulyk

**Affiliations:** 1 Division of Genetics, Department of Medicine, Brigham and Women's Hospital and Harvard Medical School, Boston, MA 02115, USA; 2 Committee on Higher Degrees in Biophysics, Harvard University, Cambridge, MA 02138, USA; 3 Department of Pathology, Brigham and Women's Hospital and Harvard Medical School, Boston, MA 02115, USA; 4 Harvard-MIT Division of Health Sciences and Technology (HST), Harvard Medical School, Boston, MA 02115, USA

## Abstract

**Background:**

Transcription factors (TFs) play a central role in regulating gene expression by interacting with *cis*-regulatory DNA elements associated with their target genes. Recent surveys have examined the DNA binding specificities of most *Saccharomyces cerevisiae *TFs, but a comprehensive evaluation of their data has been lacking.

**Results:**

We analyzed *in vitro *and *in vivo *TF-DNA binding data reported in previous large-scale studies to generate a comprehensive, curated resource of DNA binding specificity data for all characterized *S. cerevisiae *TFs. Our collection comprises DNA binding site motifs and comprehensive *in vitro *DNA binding specificity data for all possible 8-bp sequences. Investigation of the DNA binding specificities within the basic leucine zipper (bZIP) and *VHT1 *regulator (VHR) TF families revealed unexpected plasticity in TF-DNA recognition: intriguingly, the VHR TFs, newly characterized by protein binding microarrays in this study, recognize bZIP-like DNA motifs, while the bZIP TF Hac1 recognizes a motif highly similar to the canonical E-box motif of basic helix-loop-helix (bHLH) TFs. We identified several TFs with distinct primary and secondary motifs, which might be associated with different regulatory functions. Finally, integrated analysis of *in vivo *TF binding data with protein binding microarray data lends further support for indirect DNA binding *in vivo *by sequence-specific TFs.

**Conclusions:**

The comprehensive data in this curated collection allow for more accurate analyses of regulatory TF-DNA interactions, in-depth structural studies of TF-DNA specificity determinants, and future experimental investigations of the TFs' predicted target genes and regulatory roles.

## Background

Transcription factors (TFs) control and mediate cellular responses to environmental stimuli through sequence-specific interactions with *cis *regulatory DNA elements within the promoters and enhancers of their target genes, thus directing the expression of those genes in a coordinated manner. Because of the importance of TFs and their DNA binding sites in targeting gene regulation, numerous studies have aimed to identify the DNA binding specificities and target genes of these regulatory factors. *Saccharomyces cerevisiae *is one of the most extensively studied eukaryotic organisms and has served as an important model in understanding eukaryotic transcriptional regulation and regulatory networks [1,2]. Computational approaches, including phylogenetic footprinting [3,4], sequence analysis of sets of functionally related genes [5], and analysis of co-expressed groups of genes [6], as well as experimental approaches, including *in vivo *chromatin immunoprecipitation (ChIP) followed by microarray readout (ChIP-chip) [7], protein binding microarrays (PBMs) [8-11], and *in vitro *mechanically induced trapping of molecular interactions (MITOMI) [12], have sought to determine and catalog the DNA binding specificities of *S. cerevisiae *TFs.

Recently, several studies [10-12] have examined at high resolution (that is, at the level of '*k*-mer' binding site 'words') the *in vitro *DNA binding preferences of a large number of *S. cerevisiae *TFs. These studies used high-throughput *in vitro *techniques (PBM or MITOMI) to measure the DNA binding specificities of TFs for all possible 8-bp DNA sequences (8-mers), and used the resulting data to derive DNA binding site motifs. In addition to the comprehensive nature of the *in vitro *data reported in these studies (that is, covering all possible 8-mers), these data reflect the direct DNA binding preferences of the tested TFs; in contrast, ChIP data sometimes reflect indirect DNA binding of the immunoprecipitated TF by recruiting TFs [13]. The *in vitro *data reported in these studies are complementary to ChIP data, in that the *in vitro *data provide higher-resolution measurements of DNA binding preferences compared to ChIP (8 bp versus hundreds of base pairs, respectively) and they test the intrinsic DNA binding specificity of a TF in the absence of any protein co-factors or competitors (such as other TFs or nucleosomes).

There is substantial overlap among the sets of TFs tested in the *in vitro *studies. Badis *et al. *[10] and Zhu *et al. *[11] report PBM data for 112 and 89 TFs, respectively, with data for 64 TFs reported by both studies. Fordyce *et al. *[12] report MITOMI data for 28 TFs, 20 of which also have PBM data reported by either Badis *et al. *or Zhu *et al. *Despite the large overlap among these studies, a comprehensive comparison, evaluation and integration of these different data sets has been lacking. Where DNA binding site motifs have been reported in several studies, in most cases the motifs agree across the studies, but it is unclear which motif would be best to use, such as for prediction of putative TF binding sites.

Here, we analyzed the existing *in vitro *DNA binding specificity data from prior studies [10-12] and complemented those data with new PBM data for 27 DNA-binding proteins, with the goal of creating a single, curated resource of comprehensive DNA binding specificity data for *S. cerevisiae *TFs. We analyzed a total of 150 TFs, 90 of which have now been tested in at least two different studies. For each TF we report both its optimal DNA binding site motif that we selected from the four surveys (evaluated according to several criteria, including concordance with *in vivo *data) and the corresponding DNA binding specificity measurements for all 8-mer DNA sequences.

This curated collection allowed for an in-depth investigation of the DNA binding specificities within an important eukaryotic family of TFs (the basic leucine zippers, or bZIPs), resulting in novel findings of plasticity in TF-DNA recognition. We found that the newly characterized *VHT1 *regulator (VHR) TFs (Vhr1 and Vhr2) recognize bZIP-like DNA motifs, while the bZIP TF Hac1 recognizes a motif highly similar to the canonical E-box motif of basic helix-loop-helix (bHLH) TFs. We also observed that 39 of the 150 yeast TFs in our curated list have distinct primary and secondary motifs, likely corresponding to different modes of binding DNA and potentially different regulatory functions. Thus, our results illustrate how one can take advantage of the comprehensive nature of the *in vitro *DNA binding specificity data in our curated collection to identify novel structural and gene regulatory features of TF-DNA interactions. These comprehensive data will allow for more accurate computational analysis of gene regulatory networks and directed experimental investigations of their predicted target genes and regulatory roles, as well as more in-depth structural studies of TF-DNA specificity determinants.

## Results and discussion

### Curated collection of high-resolution *in vitro *DNA binding data for *S. cerevisiae *TFs

We compiled *in vitro *DNA binding specificity data from three prior large-scale studies [10-12] (Tables S1 and S2 in Additional file 1) and complemented them with newly generated universal PBM data for 27 TFs (see below), with the goal of generating the most up-to-date and comprehensive resource of *in vitro *DNA binding site motifs (Additional file 2) and corresponding high-resolution DNA binding data, represented here as measurements of DNA binding specificity for all possible 8-bp sequences (Additional file 3). Briefly, the relative binding preference for each 8-mer on universal PBMs is quantified by the PBM enrichment score (E-score) [14]. The E-score is a modified form of the Wilcoxon-Mann Whitney statistic and ranges from -0.5 (least favored sequence) to +0.5 (most favored sequence), with values above 0.35 corresponding, in general, to sequence-specific DNA binding of the tested TF [8]. We used the 8-mer data to compute DNA binding site motifs using the Seed-and-Wobble algorithm [8,15]. For each TF we ranked all the 8-mers according to their E-scores and chose the highest scoring 8-mer as a seed to construct a primary motif. The PBM data were then analyzed to determine if there are spots of high signal intensity that do not score well by the primary motif; the 8-mer data were then analyzed to derive a secondary motif that does explain the residual binding to the DNA microarray probes. The set of 8-mers represented by a secondary motif can be of similar affinity as those of the primary motif, or can be of distinctly lower affinity [16]. We note that the E-scores we report for 8-mer seeds of secondary motifs are based on the initial ranking of all 8-mers and thus are directly comparable with the E-scores reported for primary motif 8-mers. Secondary motifs derived from PBM data are unlikely to be attributable to a motif-finding artifact, and TF binding to secondary motifs has been confirmed by electrophoretic mobility shift assay for six mouse TFs [16]. Supporting results from a recent PBM survey of 104 mouse TFs [16], we observed that 39 of the 150 yeast TFs in our curated list recognize distinct primary and secondary DNA motifs (discussed in detail in a separate section in the Results and discussion). We analyzed in detail one of these 39 TFs, Sko1, and found that both the primary and secondary motifs are utilized *in vivo *and that they are potentially associated with different regulatory functions of Sko1 (discussed in detail later in the Results and discussion).

Specifically, to complement the existing *in vitro *DNA binding data for *S. cerevisiae *TFs, we tested 155 proteins on universal PBMs [8]. Unlike previous studies, which focused on known and predicted TFs based on the presence of known sequence-specific DNA-binding domains (DBDs), our criteria for including candidate regulatory proteins were permissive and included many proteins without well-characterized DBDs and proteins for which we had low confidence in their being potential sequence-specific, double-stranded DNA binding proteins; thus, we did not expect many of these proteins to yield highly specific DNA binding sequences typical of TFs, but we tested them nevertheless in an attempt to obtain the most comprehensive TF DNA binding specificity collection possible. We also included proteins for which the existing *in vitro *data were of low quality or did not agree with previous literature (for example, Ste12, Ecm22). Of the 155 proteins attempted on universal PBMs, 27 resulted in sequence-specific DNA binding. In total, our collection encompasses 150 TFs, 90 of which have been examined in at least two different studies (Tables S3 in Additional file 1 and Additional file 4). For each of these 90 TFs, we chose the highest quality motif based on the agreement between the motif and other *in vitro *binding data, the enrichment of the motif in ChIP-chip data [7], and the quality of the raw 8-mer data used to generate the motif (Additional file 1). The enrichment of a motif in a ChIP-chip data set was expressed as an area under the receiver operating characteristic (ROC) curve (AUC); an AUC of 1 corresponds to perfect enrichment, while an AUC of 0.5 corresponds to the enrichment of a random motif. The selected DNA binding site motifs for the 150 TFs (represented as position weight matrices (PWMs)) are available in Additional file 2 with the source of each motif specified in Table S3 in Additional file 1.

**Figure 1:**
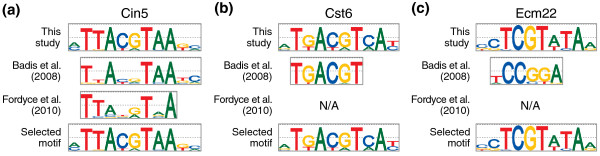
**Selecting DNA binding site motifs for our curated collection**. **(a) **The *in vitro *motifs for TF Cin5 are very similar, but not equally enriched in the ChIP-chip data (see main text). **(b) **The Cst6 *in vitro *motif reported by Badis *et al. *[10] is a truncated version of the Cst6 motif reported in this study. The latter better matches TGACGTCA, the known site for the ATF/CREB family of bZIP TFs, of which Cst6 is a member. **(c) **For TF Ecm22 we selected the motif obtained in this study (which is different from the motif previously reported by Badis *et al. *[10]). The selected motif matches the sterol regulatory element TCGTATA, which had been reported to be bound by Ecm22 (and also its close paralog, Upc2). N/A, not available in Fordyce *et al. *[12].

For most TFs analyzed here, the motifs reported in different studies look very similar, but are not equally enriched in the ChIP-chip data. For example, the Cin5 motifs reported in this study, Badis *et al. *[10], and Fordyce *et al. *[12] are very similar (Figure 1a), but their AUC enrichment in the Cin5_YPD ChIP-chip data [7] is 0.89, 0.88, and 0.81, respectively; thus, we chose the Cin5 motif newly reported in this study. For other TFs, the motif reported in one study is a truncated version of the motif reported in a different study, as illustrated in Figure 1b for Cst6; in this case, we chose the DNA binding site motif reported in this study because it better matches TGACGTCA, the known site for the ATF/CREB family of bZIP TFs [17], of which Cst6 is a member. There are also a few TFs for which the motifs reported in different studies do not match, as shown in Figure 1c for Ecm22; in this case we turned to the existing literature and found that Ecm22 (and its close paralog Upc2) bind to the sterol regulatory element (SRE; TCGTATA) [18], which clearly matches the motif reported in this study, but not the motif reported by Badis *et al. *[10]. Overall, no single study clearly outperformed the other studies in terms of quality of the reported motifs (Additional file 4).

**Figure 2:**
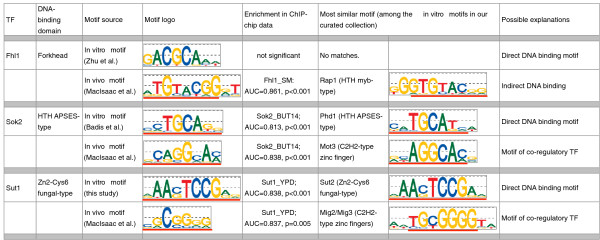
**Examples of TFs for which the *in vitro *and *in vivo *DNA binding site motifs are different**. For both the *in vitro *and *in vivo *motifs of the three TFs we show their enrichment in the corresponding ChIP-chip data set, measured by the AUC and the associated *P*-value. We also show the *in vitro *motifs (from our curated collection) that are most similar to the *in vitro *and *in vivo *motifs of the three TFs of interest (the red lines indicate which parts of the motifs are similar). We notice that in all three cases the *in vivo *motifs are similar to the DNA binding site motifs of TFs from a different structural class. This suggests that in each of the three cases the *in vivo *motif (derived from ChIP-chip data) does not belong to the TF profiled by ChIP, but either to a co-regulatory TF (which binds a common set of targets as the profiled factor), or to a mediating TF (which binds DNA directly and mediates the interaction between the TF profiled by ChIP and the DNA - in this case we hypothesize that the TF tested by ChIP binds DNA indirectly thought the mediating TF). Motif sources from this study and Zhu *et al. *[11], Badis *et al. *[10], and MacIsaac *et al. *[20].

We also compared the curated, *in vitro *DNA binding site motifs against motifs derived from the *in vivo *ChIP-chip data of Harbison *et al. *[7], which were available for 85 TFs (Table S5 in Additional file 1 and Additional file 5). In most cases, the *in vivo *and *in vitro *motifs are in good agreement, and we did not find that the *in vivo *motif explains the ChIP-chip data either better or worse than the *in vitro *motifs (data not shown). We did find, however, 15 TFs for which the *in vivo *and *in vitro *motifs are different (Figure 2; Additional file 6), typically because the TF profiled by ChIP does not bind DNA directly (in which case the motif of the mediating factor is recovered from the ChIP data), or alternatively because a motif of a co-factor is also enriched in the sequences bound by ChIP (and is reported as the ChIP-derived motif) (Additional file 6). For example, our analysis supports a model whereby Fhl1 binds DNA indirectly through a mediating factor, Rap1 [19], since the Fhl1 motif is not significantly enriched in the ChIP data whereas the Rap1 motif is, and the two TFs belong to different structural classes and thus are not anticipated to have similar DNA binding site motifs. In Figure 2 we show the *in vitro *and *in vivo *motifs for Sok2 and Sut1, members of the HTH APSES and Zn_2_Cys_6 _families, respectively. The Sok2 and Sut1 *in vitro *motifs are in excellent agreement with the PBM-derived motifs for the highly similar TFs Phd1 and Sut2, respectively, but are significantly different from the motifs derived from ChIP-chip data [7,20]. As shown in Figure 2, both the PBM-derived motifs and the ChIP-derived motifs of Sok2 and Sut1 are significantly enriched in the ChIP-chip data. In such cases we conclude that the PBM-derived motifs reflect the direct DNA binding specificities of the TFs, while the ChIP-derived motifs may represent the DNA binding specificities of co-regulatory TFs (often belonging to different DBD structural classes) that bind *in vivo *to many of the genomic regions bound by the TFs profiled by ChIP. In total, we noticed discrepancies between *in vitro *and *in vivo *TF binding data for 15 of the 150 TFs in our curated list. These cases are discussed in detail in Additional files 1 and Additional file 6 and later in the Results and discussion section we present a thorough re-analysis of the *in vivo *ChIP-chip data of Harbison *et al. *[7] using our curated collection of *in vitro *motifs.

### Comprehensive PBM data reveal new insights into the DNA binding specificities of bZIP and VHR TFs

**Figure 3:**
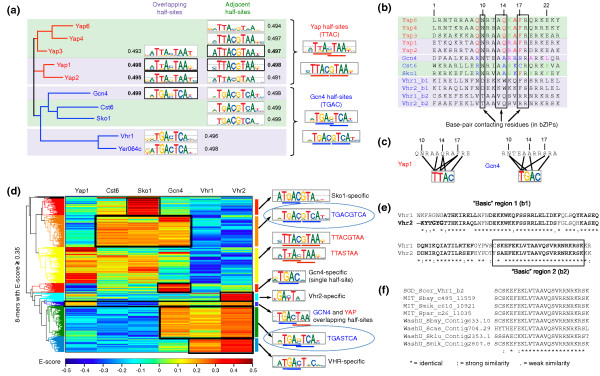
**bZIP and VHR TFs**. **(a) **Phylogeny and PBM-derived motifs for the eight bZIP and two VHR proteins analyzed in this study. The evolutionary tree was built from a ClustalW2 [59] multiple sequence alignment of the DBDs of the ten proteins, as annotated in UniProt [60]. Green and magenta backgrounds correspond to TFs that bind primarily to overlapping or adjacent half-sites, respectively. TFs that bind Yap-like half-sites are shown in red. TFs that bind Gcn4-like half-sites are shown in blue. All motif logos were generated using EnoLOGOS [58], based on motifs generated from PBM data in this study and Zhu *et al. *[11] using the Seed-and-Wobble algorithm [8,15]. The numbers next to the motif logos represent the E-scores of the 8-mer seeds used to construct the motifs [8]. For proteins that bind both overlapping and adjacent half-sites, the motif corresponding to the largest seed E-score (sometimes referred to as the primary motif) is shown in a black box. **(b) **ClustalW2 multiple sequence alignment of the basic regions of bZIP proteins against the DBDs of VHRs. The Vhr1 and Vhr2 regions shown are the ones that best align to the eight basic regions considered, and they correspond to the first putative VHR basic region (see (e)). The residues shown in red and blue are important for YAP-like versus Gcn4-like half-site specificity. The residues shown in green and magenta are important for overlapping versus adjacent half-site binding. **(c) **Recognition of Yap-like and Gcn4-like half-sites [30,61]. **(d) **Heat map of the DNA-binding preferences of Yap1 (as a representative of the Yap subfamily), Cst6, Sko1, Gcn4, Vhr1, and Vhr2. The rows correspond to 8-mers with an E-score ≥0.35 for any of the six TFs; the columns correspond to the TFs. The E-score scale is shown at the bottom. Black boxes indicate the 8-mers that correspond to various motifs (shown on the right). **(e) **Alignment of the full DBDs of Vhr1 and Vhr2. Residues that fold into alpha-helices (according to PSIPRED [62]) are shown in bold. Black boxes show the two putative basic domains in VHR proteins. **(f) **Alignment of the second putative VHR basic region to basic regions of the eight bZIPs analyzed in this study.

Comprehensive data on the DNA binding specificities of TFs, such as PBM data, can reveal insights into the differences in DNA sequence preferences among TFs within the same structural class [21-25]. Here, we studied in detail eight bZIP DNA-binding proteins: five Yap (yeast AP-1) proteins and three additional bZIP proteins (Cst6, Gcn4, and Sko1) for which high-resolution PBM data are available (this study and Zhu *et al. *[11]). In Figure 3a, next to each DNA binding specificity motif logo we show the E-score of the 8-bp seed sequence used to construct the PBM-derived motif [8]. E-scores above 0.45 generally indicate highly preferred binding sequences.

The bZIP DBD consists of two functionally distinct subdomains: the basic region (which makes specific DNA contacts) and the leucine zipper region (which is involved in dimerization) [26]. Proteins of this class homo- and heterodimerize, and typically bind either overlapping or adjacent TGAC half-sites, based on which bZIPs are often categorized into two subclasses: AP-1 factors that prefer the TGA(C|G)TCA motif and ATF/CREB factors that prefer TGACGTCA [17]. The *S. cerevisiae *genome encodes 14 bZIP factors, 8 of which belong to the fungal-specific Yap subfamily [27] and bind overlapping or adjacent T**T**AC half-sites instead of T**G**AC half-sites. Our results on the DNA binding specificities of bZIP proteins largely agree with what has been reported previously based on ChIP data: Yap3, Yap4 and Yap6 prefer adjacent TTAC half-sites, Yap1 and Yap2 prefer overlapping TTAC half-sites [28,29], and Gcn4 prefers overlapping TGAC half-sites [30]. Also in agreement with previous reports [17], we find that AP-1 bZIPs (Yap1, Yap2, and Gcn4), which generally prefer overlapping half-sites, bind to adjacent half-sites with almost equal affinity: the E-scores of the 8-bp seeds for the primary and secondary DNA binding site motifs of Yap1, Yap2, and Gcn4 are very close or even identical (Figure 3a). Previous reports also suggest that ATF/CREB bZIPs, which generally prefer adjacent half-sites, bind poorly to overlapping half-sites [17]. However, our high-resolution PBM data indicate that while this is true for Cst6, Sko1, Yap4, and Yap6, the TF Yap3 can also bind overlapping TTAC half-sites with high specificity (the seed E-score for the secondary Yap3 motif is 0.493, close to that of the Yap3 primary motif seed: 0.497). This finding suggests that, despite the fact that some of the residues important for half-site spacing specificity have been identified (Figure 3b; Additional file 1), it is not yet fully understood how these proteins achieve their specificity. It is possible that specific combinations of residues (not necessarily DNA-contacting residues) determine the preference for binding to overlapping versus adjacent half-sites.

**Figure 4:**
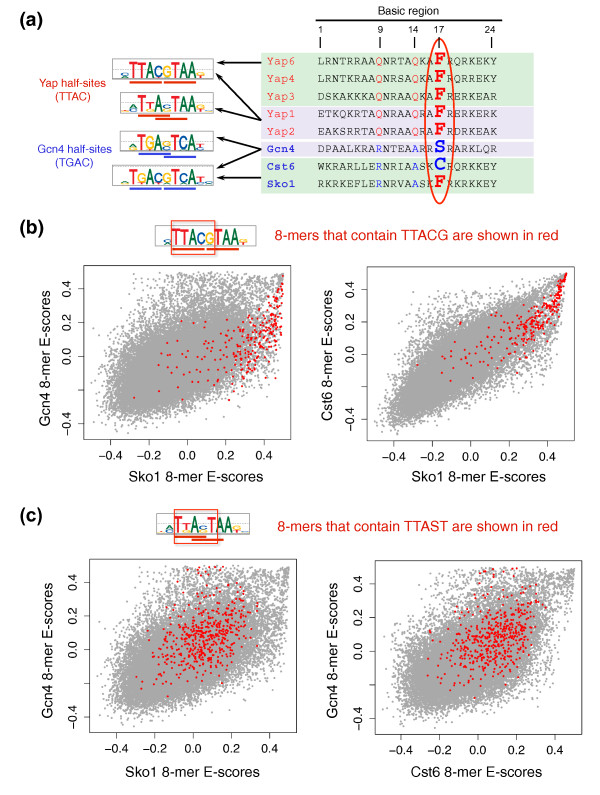
**Position 17 in the basic region of bZIP TFs is important for specifying Yap versus Gcn4 half-sites**. **(a) **ClustalW2 alignment of the basic regions of eight yeast bZIP proteins, and the corresponding DNA binding site motifs. **(b) **Sko1 (which contains a phenylalanine at position 17 in the basic region, similar to Yap proteins) has a stronger preference for adjacent Yap half-sites compared to Gcn4 and Cst6 (which contain serine and cysteine at position 17, respectively). **(c) **The trend observed in (b) is not simply due to the fact that Sko1 prefers adjacent half-sites to overlapping half-sites. If this were the case, we would expect Gcn4 to bind overlapping Yap half-sites with higher affinity than Sko1, but we do not observe such a trend.

Since the Yap family of bZIP proteins was first characterized [27], the basic region residues Gln9, Gln14, Ala16, and Phe17 (Figure 3b) have been reported to provide specificity for Yap-like half-sites (TTAC). However, we noticed that Sko1, a typical bZIP protein that binds to adjacent TGAC half-sites [31], also has a phenylalanine at position 17 of the basic region. Our high-resolution PBM data allowed us to analyze in more detail the specificity of Sko1 for TGAC versus TTAC half-sites. As shown in Figure 4, Sko1 does indeed have a higher preference for TTAC half-sites than do the typical bZIP proteins Gcn4 and Cst6. This finding confirms the importance of residue Phe17 for conferring Yap-like versus Gcn4-like half-site preference.

In addition to bZIP proteins, we analyzed PBM data for Vhr1 and Yer064c, members of the fungal VHR (*VHT1 *regulator) class of DNA-binding proteins, for which only a single DNA consensus sequence had been reported previously [32]. The Yer064c protein sequence and its DNA binding specificity are very similar to those of Vhr1 (Figure 3), so we henceforth refer to Yer064c as Vhr2. Our PBM data indicate that these VHR proteins bind Gcn4-like motifs despite the fact that their DBD is of a different structural class. As shown in the dendrogram in Figure 3a, the DBDs of Vhr1 and Vhr2 are closely related to each other, but not to DBDs of bZIP proteins. Furthermore, in an alignment of the Vhr1 and Vhr2 DBDs against the basic regions of bZIP proteins (Figure 3b), it is apparent that essential DNA-contacting residues in the basic region of bZIPs (for example, Asn10, Arg18; Figure 3c) are not found in the VHR domain.

In an attempt to identify the DNA-contacting region in the VHR domain, we analyzed the protein sequences of Vhr1 and Vhr2 and found that these proteins have two putative basic regions, which we denote as b1 and b2 (Figure 3e). The second basic region seems to align better to the basic regions of bZIP proteins (Figure 3b) than does the first basic region, and it is also more conserved across *Saccharomyces *species in the *sensu stricto *clade (Figure 3f; Figure S1 in Additional file 1). These observations suggest that the second basic region in the VHR domain is more likely to be the one that interacts with DNA. Identifying the exact DNA-contacting residues and key specificity determinants will require further experimentation, involving mutagenesis experiments and structural analyses. It would be interesting to see whether VHR proteins contact DNA in a way similar to bZIPs or if they utilize a completely different structural mode of protein-DNA recognition.

We also note that VHR proteins bind exclusively to overlapping TGAC half-sites, unlike AP-1 proteins (including Gcn4), which can bind both overlapping and adjacent half-sites (Figure 3a,d). We are not aware of any AP-1 protein that binds exclusively to overlapping half-sites. As shown in Figure S2 in Additional file 1 all AP-1 proteins with PBM data in UniPROBE can also bind adjacent half-sites, unlike VHR proteins. All this evidence indicates that VHR is a distinct DBD structural class, despite the fact that there is significant overlap between the DNA sequences preferred by VHR and bZIP proteins.

### Yeast Hac1 is a bZIP TF whose specificity is more similar to bHLHs than bZIPs

**Figure 5:**
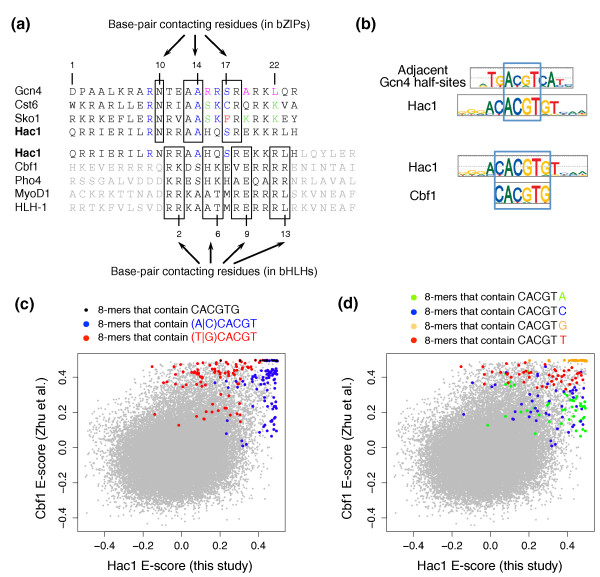
**DNA binding specificity of Hac1**. **(a) **Alignment of the Hac1 basic region against the basic regions of the eight bZIP proteins shown in Figure 3. Hac1 is most similar to Gcn4 in terms of DNA-contacting residues. The font color scheme is the same as in Figure 3; residues indicated in gray are not part of the basic region (of either bZIP or bHLH proteins). **(b) **PBM-derived motifs for Gcn4, Hac1, and Cbf1. Despite the fact that Hac1 belongs to the bZIP structural class, the Hac1 motif barely matches the motif of the bZIP protein Gcn4. Instead, the Hac1 motif resembles E-box motifs bound by bHLH proteins (such as Cbf1). **(c, d) **In-depth comparison of the DNA binding specificities of Hac1 and Cbf1. The scatter plots show 8-mer E-scores.

In the above analysis of bZIP factors, we did not include Hac1, an essential TF involved in the unfolded protein response in *S. cerevisiae *[33], for which high-resolution PBM data are available (this study and Badis *et al. *[10]). According to key residues in its DBD (Figure 5a, residues marked in blue), Hac1 is a bZIP factor that should bind either overlapping or adjacent TGAC half-sites. However, its primary PBM-derived motif, obtained using the full-length protein in PBM experiments, is most similar to an E-box, which is characteristic of bHLH proteins such as Cbf1 (Figure 5b). We note that Hac1 does not have a secondary DNA binding site motif that resembles a bZIP motif. Furthermore, its E-box motif appears to be utilized by Hac1 *in vivo*: this motif is significantly enriched in the Harbison *et al. *[7] Hac1_YPD ChIP-chip dataset (AUC = 0.6906, *P *= 0.005), while typical bZIP motifs (TGAsTCA and TGACGTCA) are not significantly enriched (*P *> 0.1) in that same ChIP-chip dataset.

Visual inspection of the Hac1 DBD revealed a portion that aligns well to the basic regions of bHLH proteins, especially those of the human myogenic factor MyoD1 and its *Caenorhabditis elegans *ortholog HLH-1. Hac1 shares many of the DNA-contacting residues [22] with the myogenic bHLHs (Figure 5a). However, unlike the myogenic factors, which prefer the hexamers CACCTG and CAGCTG [34], Hac1 strongly prefers CACGTG; thus, we compared the DNA binding specificity of Hac1 with that of the *S. cerevisiae *TF Cbf1, which also strongly prefers CACGTG.

Although the motifs of Hac1 and Cbf1 are very similar, the 8-mer PBM data reveal that there are significant differences in their DNA binding specificities. Whereas Cbf1 has a strong preference for G or T upstream of the CACGTG core motif, Hac1 prefers A or C (Figure 5c). Similarly, while both Hac1 and Cbf1 bind CACGT with high affinity, Cbf1 strongly prefers CACGT(G|T) to CACGT(A|C) (Figure 5d). These differences in specificity are supported by the PBM data from Badis *et al. *[10], which show the same trends (Figure S3 in Additional file 1). Thus, despite the fact that the Hac1 and Cbf1 motifs look very similar, there are substantial differences in the DNA binding preferences of these two proteins, which likely contribute to their *in vivo *specificities. Indeed, all sequences bound by Cbf1 in a ChIP-chip experiment performed on yeast grown in rich medium (Cbf1_YPD) [7] contain (T|G)CACGT, while only 4 of the 16 sequences bound by Hac1 in this same condition (dataset Hac1_YPD) contain this motif, and 2 of these 4 sequences also contain the (A|C)CACGT motif that is preferred by Hac1 (Figure 5c). In conclusion, Hac1 seems to be a hybrid between a bHLH and a bZIP protein. Its DBD strongly resembles the domains of bZIP proteins, although part of its basic region shows strong similarity with the basic regions of bHLHs (Figure 5a); the similarity to bHLH proteins likely explains why it can bind an E-box motif. However, the DNA binding specificity of Hac1, as analyzed here by PBM, is not that of a typical bHLH protein. In-depth structural investigations of Hac1 and its homologs in other organisms would reveal whether its DNA-contacting residues are indeed the same as in bHLH proteins and might provide insights into the evolutionary relationship between bZIP and bHLH domains.

### *S. cerevisiae *TFs with two distinct DNA binding site motifs

**Figure 6:**
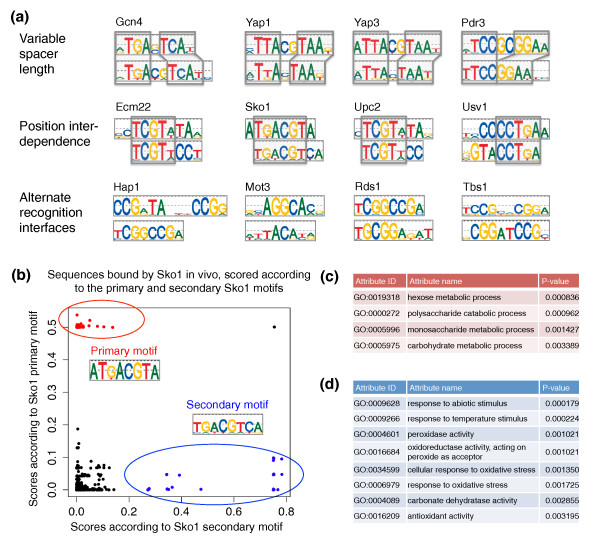
**Primary and secondary DNA binding site motifs**. **(a) **Different categories of TFs with two distinct modes of binding DNA. **(b) **Scatter plot of GOMER scores for regions bound *in vivo *by Sko1, according to the primary versus the secondary Sko1 DNA binding site motifs. Regions that score highly according to the primary but not the secondary motifs are shown in red. Regions that score highly according to the secondary but not the primary motifs are shown in blue. **(c) **Gene Ontology (GO) categories enriched in the regions that score highly according to the primary but not the secondary Sko1 motif. **(d) **GO categories enriched in the regions that score highly according to the secondary but not the primary Sko1 motif. See main text and Additional file 1 for details.

Prior surveys have not investigated whether *S. cerevisiae *TFs recognize primary and secondary DNA binding site motifs, as do numerous mouse TFs [16]. We found that 39 of the 150 TFs in our curated list recognize two distinct motifs (Figure 6a; Figures S4 and S8 in Additional file 1). For 5 of the 39 TFs (Leu3, Lys14, Tea1, Ylr287c, and Zap1), the two motifs correspond to a full motif versus a single half-site; while this might be an artifact of Seed-and-Wobble, the algorithm used to compute the motifs from PBM data, the fact that TFs can bind DNA both as homodimers and as monomers is supported by results reported in a recent survey of mouse TFs using PBMs [16] and a recent survey of human TFs using an *in vitro *selection approach [35]. We note that for two TFs that have ChIP-chip data available (Leu3 and Zap1) [7], the full motif was more enriched than the half-site, which is consistent with the model that these TFs bind DNA *in vivo *as homodimers, at least in the conditions tested thus far by ChIP. 

The remaining 34 TFs with secondary DNA motifs can be grouped into three categories, analogous to categories noted previously for mouse TFs [16]. We found five variable spacer length TFs (Gcn4, Pdr3, Yap1, Yap2, and Yap3), for which the primary and secondary motifs contain similar half-sites separated by different spacer lengths. For some of these TFs (Yap1 and Gcn4) the secondary motifs were bound nearly as well as the primary motifs, as illustrated by the fact that the 8-mer seeds for the two motifs have similar or identical E-scores (Figure 3). We found 24 cases of position interdependence TFs (Figure 6a; Figure S4 in Additional file 1). For each of these 24 TFs, the primary and secondary motifs share a common portion that typically spans three to five (often adjacent) nucleotide positions, but that are otherwise different. For example, the primary and secondary Ecm22 motifs share the core TCGT(A|T), but the primary motif ends in TA(A|G) while the secondary motifs ends in CCT. In such cases the primary and secondary motifs cannot be combined into a single PWM because the PWM model assumes independence between nucleotide positions. This implies that in order to accurately represent the DNA binding specificity of these TFs using standard PWM models, one has to consider both the primary and secondary motifs. The secondary motifs of five TFs were not readily explainable by either variable spacer length or position interdependence. These TFs, classified as alternative recognition interfaces, might bind DNA either through alternative structural features [36] of the DBD or by adopting alternative conformations.

Given the high number of TFs with secondary DNA motifs, we asked whether both modes of binding DNA are used *in vivo *and whether the primary and secondary motifs of a TF are associated with different regulatory functions. We first attempted to use the ChIP-chip data from the large-scale study of Harbison *et al. *[7] to address these questions. However, of the 34 TFs classified as variable spacer length, position interdependence, or alternative recognition interfaces, 12 TFs are not represented in the ChIP-chip data and for another 11 TFs neither the primary nor the secondary motif is enriched in the ChIP-chip data. Of the remaining 11 TFs, 5 have fewer than 30 bound sequences in the ChIP-chip data (for this analysis of primary and secondary motifs, we required a minimum of 30 bound sequences), and 6 TFs were tested only in rich medium although they are known to function in different cellular conditions. Thus, the ChIP-chip data of Harbison *et al. *[7] cannot be used to address the question of whether the primary and secondary motifs may be associated with different biological functions of the same TF. This question needs to be addressed for each TF individually using high-quality, high-resolution *in vivo *DNA binding data collected under cellular conditions where the TF is known to be active. While generating or compiling such data is beyond the scope of this paper, for one of the TFs with a secondary motif, Sko1, suitable ChIP-chip data were readily available and we analyzed them in detail (see below).

### Primary and secondary DNA binding site motifs for TF Sko1 are associated with different regulatory functions

When the *SKO1 *gene was first cloned [31], it was reported to encode a bZIP protein that binds to the ATF/CREB motif (TGACGTCA) but that can also bind a slightly different site (ATGACGTACT) in the promoter region of *SUC2 *(a sucrose hydrolyzing enzyme), acting as a repressor of *SUC2 *transcription [31]. These two sites are perfect matches for the secondary and primary Sko1 motifs - TGACGTCA and ATGACGTA - respectively.

Recently, Ni *et al. *[37] analyzed the temporal DNA binding of several TFs involved in osmotic stress response in *S. cerevisiae*, including Sko1, by ChIP-chip on high-density oligonucleotide arrays. The ChIP-chip experiments were performed after incubation of the yeast in high salt concentration for 0, 5, 15, 30, and 45 minutes; for each time point, Ni *et al. *reported the regions bound by Sko1 at a false discovery rate of 0.01. Each bound region located within 1 kb of a gene was assigned to that gene [37]. We scored the regions bound by Sko1 *in vivo *according to the primary and the secondary motifs using the GOMER model [38], which computes the probability that a DNA sequence is bound by a TF with a particular PWM. Figure 6b shows a scatter plot of these scores for the regions bound by Sko1 *in vivo *after salt treatment for 5 minutes; we obtained similar results for other time points (data not shown). There are high-scoring regions for both the primary and the secondary Sko1 motifs, which suggests that both motifs are utilized *in vivo*. 

Next, for the bound regions that score highly according to the primary motif but low according to the secondary motif (marked in red in Figure 6b), we performed a Gene Ontology (GO) annotation term enrichment analysis of the bound genes using FuncAssociate2 [39] and found significant enrichment (*P *< 0.005; Additional file 1) for the categories hexose metabolic process, polysaccharide catabolic process, monosaccharide metabolic process, and carbohydrate metabolic process (Figure 6c). Similarly, we analyzed the ChIP-bound regions that score highly according to the secondary motif but low according to the primary motif (marked in blue in Figure 6b) and found that different GO categories were significantly enriched, including peroxidase activity, cellular response to oxidative stress, response to oxidative stress, and antioxidant activity (Figure 6d), which indicates that the secondary Sko1 motif is associated primarily with genes involved in oxidative stress. In addition to its critical role during osmotic stress response [37], Sko1 has also been shown to regulate genes encoding enzymes implicated in protection from oxidative damage [40]; our analysis suggests that Sko1 performs this function through its secondary DNA binding site motif. We also find that the Sko1 secondary motif may be used to regulate heat response genes, which suggests a novel regulatory function for this TF.

Sko1 is not the only TF that utilizes both the primary and the secondary motifs *in vivo*. Evidence from small-scale studies shows that Gcn4, which binds primarily to TGACTCA sites upstream of amino acid biosynthetic genes [41], also binds with high affinity to the secondary motif TGACGTCA and activates transcription through this site *in vivo *[42]. We anticipate that future in-depth analyses of high-quality ChIP-chip data, similar to the analysis we performed for Sko1, will show that many of the secondary DNA binding site motifs of yeast TFs are used *in vivo*, and that they are associated with different regulatory functions of the TF.

### Predicted functions of the newly characterized TFs Vhr1 and Vhr2

We used the PBM data in a sequence-based promoter analysis as described previously [11] to predict target genes and biological roles for the newly characterized proteins (Additional file 7). Briefly, this method scores genes according to the presence of PBM-derived DNA binding sequences in their promoter regions; although the presence of a binding site sequence does not guarantee *in vivo *TF binding and regulation of the downstream gene, this analysis provides computational predictions of TF regulatory targets and associated biological functions. This analysis allowed us to make initial function predictions for two newly characterized proteins, Vhr1 and Vhr2, with poorly annotated functions. The top 200 predicted target genes of Vhr1, scored according to the PBM 8-mer data (Additional files 1 and 8), are significantly enriched [39] (P_adj ≤ 0.001) for the GO categories small molecule biosynthetic process, small molecule metabolic process, and cofactor binding (Additional file 7), consistent with its previously discovered role in regulating *VHT1 *(Vitamin H transporter) and *BIO5 *in a biotin-dependent manner [32]. Additional, novel roles for Vhr1 are predicted for cellular nitrogen compound biosynthetic process and the biosynthesis and metabolism of arginine, glutamine, serine, and other amino acids (Additional file 7). Because of its highly similar DNA binding specificity, Vhr2 is also predicted to function in most of these same biological processes.

Gene expression data from a large microarray compendium containing 352 datasets from 233 published studies [43] lend additional support for a role of Vhr1 in amino acid and nitrogen-related biological processes. Using the SPELL search engine [43], we find that gene expression microarray experiments involving leucine [44] and histidine limitation [45] are among those ranking highest for Vhr1 differential gene expression. Additionally, when considering the 50 genes most similarly expressed as Vhr1 across all datasets, the significantly enriched GO terms (*P *< 0.05, Bonferroni-corrected Fisher's exact test [43]) include cellular amino acid biosynthetic process and cellular nitrogen compound biosynthetic process; similar enrichment is observed for Vhr2. These amino acid-related roles for Vhr2 are further supported by its known physical interaction with Ape2p [46], a leucine aminopeptidase involved in the cellular supply of leucine from external substrates as well as in general peptide metabolism [47,48]. Finally, we used the CRACR algorithm [49] to survey approximately 1,700 gene expression microarray data sets to identify conditions in which Vhr1 or Vhr2 are predicted to regulate their target genes, and found that the putative target genes of these TFs are predicted to be significantly induced under amino acid starvation and nitrogen depletion conditions (Additional file 9).

### Inference of direct versus indirect TF DNA binding in ChIP-chip data

ChIP-chip and ChIP-Seq data, which reflect genome-wide, *in vivo *TF DNA binding, are powerful approaches for determining what genomic regions are occupied by a TF *in vivo *and thus what target genes they might regulate. Although such ChIP data are often used to derive TF DNA binding site motifs, the reported binding sites and motifs may reflect the DNA specificity of multiprotein complexes in addition to, or instead of, direct DNA binding of the profiled factor. We re-analyzed the *S. cerevisiae in vivo *ChIP-chip data of Harbison *et al. *[7] using the *in vitro *motifs for 150 TFs to determine whether the factors profiled by ChIP bind DNA directly or indirectly [13]. For each ChIP data set we computed the enrichment of the 150 primary motifs and the 39 secondary motifs in the ChIP-bound versus the ChIP-unbound sequences, as described previously [13] and in the initial section of the Results and discussion. We consider a motif significantly enriched in a ChIP data set if it has an AUC ≥ 0.65 and an associated *P* -value ≤0.005 (based on randomizations of the motif) [13].

For each ChIP-chip data set, if either the primary or the secondary motif of the profiled TF was significantly enriched, then we conclude that the factor binds DNA directly. This was the case for 71 of the 167 examined ChIP-chip data sets. For 22 additional data sets the profiled TF was enriched, but its enrichment was just below our stringent significance criteria. We analyzed these sets more closely and similarly conclude that direct DNA binding of the profiled TFs is the most likely explanation for these 22 data sets (Additional file 10). For 33 ChIP-chip data sets, the motif of the profiled TF was not significantly enriched and only the motifs of TFs with different DNA binding specificities were significantly enriched. The most likely explanation for these data sets is indirect DNA binding of the profiled factor through one of the TFs whose motifs are significantly enriched. Thus, of the 167 ChIP-chip data sets for which high-resolution *in vitro *data were available for the profiled TF, roughly half (93) can be readily explained by direct DNA binding, about 20% can be explained by indirect DNA binding, while the remaining 41 data sets were not explained by any of the *in vitro *motifs, either because the set of motifs is still incomplete, or because the analyzed ChIP-chip data were too noisy, or because the profiled TF might bind DNA directly or indirectly through association with a variety of different motifs, no one of which is responsible for a significant fraction of the regions occupied *in vivo*.

### Approaching a complete collection of TF DNA binding specificities in *S. cerevisiae*

Because of our goal of identifying previously unknown TFs and our willingness to test even low-confidence predictions of potentially sequence-specific DNA binding proteins, our criteria for including candidate regulatory proteins in this study were permissive (that is, chromatin-associated proteins or proteins simply annotated as transcriptional regulatory protein) and thus included proteins that likely do not have sequence-specific DNA binding activity. Of the 92 proteins (out of 155 attempted) that did not belong to a well-characterized DBD family that we nevertheless assayed by PBM, only 2 (Msn1, Gcr1) resulted in sequence-specific DNA binding motifs. Several classes of proteins contain structural domains that have failed to yield sequence-specific DNA binding motifs in this study or any of the previous high resolution *in vitro *studies performed for *S. cerevisiae *or mouse proteins [10-12,16]: bromodomain; c; FYVE; HhH-GPD; HHH; HTH_3; PHD; SAP; SIR2; SNF2_N; XPG_N; zf-CCCH; zf-CCHC; zf-DHHC; zf-MIZ; and zf-BED. Furthermore, both the CBFD_NFYB_HMF and Copper-fist domains have produced sequence-specific DNA binding motifs from *in vivo *ChIP-chip experiments [7,20], but have failed to do so in any of the aforementioned *in vitro *studies, most likely due to the absence of protein partners or the necessary copper ion cofactor, respectively.

Of the 27 TFs whose DNA binding specificities were determined successfully by PBMs in this study, nine lacked prior high-resolution *in vitro *DNA binding data from universal PBM or MITOMI assays: Gcr1, Hmlalpha2, Mot3, Stp1, Sut1, Upc2, Vhr1, Vhr2, and Zap1 (Figure S5 in Additional file 1 and Additional file 11). Vhr1 and Vhr2 are discussed in detail in an earlier section. Sut1, a member of the Zn_2_Cys_6 _TF family, binds the motif AASTCCGA, which is in excellent agreement with the PBM-derived motif for the highly similar Zn_2_Cys_6 _TF Sut2 [11], but differs significantly from a prior motif for Sut1 derived from *in vivo *ChIP-chip data [7,20]. As discussed above, we conclude that the ChIP-derived motif represents the DNA binding specificity of a co-regulatory TF (the ChIP-derived Sut1 motif matches the motifs of the TFs Mig1, Mig2, and Mig3; Figure 2). For 13 of the 27 factors characterized in this study, PBM data have been reported previously by Badis *et al. *[10], and for 18 of the 27 factors MacIsaac *et al. *[20] reported DNA binding site motifs derived from ChIP-chip data [7]. However, when we computed the enrichment of our PBM-derived motifs and previously reported motifs in 17 ChIP-chip data sets where these factors were profiled [7], we found that in 13 of the 17 ChIP data sets the motif reported in this study was the most significantly enriched motif (Figure S5 in Additional file 1). Thus, the new PBM data reported in this study improve on and complement the existing high-resolution DNA binding specificity data, bringing us closer to the goal of obtaining a complete set of high-resolution DNA binding specificity data for all *S. cerevisiae *TFs.

## Conclusions

In this study, we present high-resolution *in vitro *DNA binding specificity data and motifs for 27 *S. cerevisiae *TFs, including some that contain a DBD for which no high-resolution motif had existed previously (for example, Vhr1 and Vhr2). These results contribute towards a complete set of high-resolution DNA binding specificity data for all TFs in this important model organism. In particular, our *in vitro *PBM analysis of *S. cerevisiae *TF DNA binding brings the set of known yeast TFs with high-resolution DNA binding specificity data to 150 (about 85%) out of a conservative total estimate of 176 TFs likely to have inherent sequence-specific, double-stranded DNA binding activity. With the addition of a more permissive set of 40 proteins (Additional file 12) that might exhibit DNA binding specificity (total of 216), this still brings us to at least 70% coverage of all *S. cerevisiae *TF DNA binding specificities. We note that these estimates may differ from previous studies because we refer strictly to TFs with intrinsic DNA binding specificity and do not include proteins that interact with DNA only indirectly.

In total, our curated collection contains high-resolution DNA binding data for approximately 85% of all known and likely sequence-specific DNA-binding proteins in *S. cerevisiae*. The remaining approximately 15% of sequence-specific *S. cerevisiae *DNA-binding proteins might require targeted investigation or specialized strategies in order to achieve complete coverage of high-resolution DNA binding specificity data for all *S. cerevisiae *TFs. We have identified 26 proteins that either are known TFs or have demonstrated lower resolution experimental data on their DNA binding specificity, or that contain a known sequence-specific DBD; we consider these proteins as the highest confidence candidates for future high-resolution *in vitro *PBM analysis (Additional file 12). Although most of these 26 proteins are from DBD classes with known sequence-specific DNA binding activity (bZIP, homeodomain, zinc cluster, copper-fist, bHLH), their previous failed attempts by *in vitro *methods may indicate that specific small-molecule cofactors and/or protein partners may be required for specific DNA binding [22]. Investigations of the effects of post-translational modifications on TFs might also reveal requirements for DNA binding specificity or conditions for modified DNA binding specificities.

Generation of a complete set of DNA binding specificity profiles for all *S. cerevisiae *TFs might also require experimental testing of proteins of even lower confidence, or to be identified by other criteria, for having potential sequence-specific DNA binding activity. Considering the set of all 222 proteins identified from previous TF candidate lists [7,10,11] and updated annotations in the *Saccharomyces *Genome Database [50], we identified 40 proteins (Additional file 12) either that contain putative nucleic acid binding domains (Myb; zf-C2H2) found in other proteins that exhibit sequence-specific DNA binding, or that are known to interact with DNA or to be involved in transcriptional regulation, but for which it is currently unknown if they bind DNA directly in a sequence-specific manner (we note that availability of a DNA binding site motif from ChIP-chip data cannot be considered evidence of direct DNA binding of the TF tested by ChIP, as some factors may bind DNA only indirectly as part of transcriptional regulatory complexes [13]). Several of these proteins belong to multisubunit complexes (for example, Hap2/3/4/5 complex) and may need to be examined for DNA binding specificity in the context of their protein partners [51]. We annotated a set of 156 proteins as unlikely (Additional file 12) to possess sequence-specific DNA binding activity since they either contain protein structural domains that have never successfully yielded a motif from this or prior large-scale *in vitro *surveys of TF DNA binding specificity, or interact with DNA indirectly, or lack prior literature evidence for direct sequence-specific DNA binding. Finally, in addition to traditional sequence-specific DNA binding site motifs, DNA structural motifs such as the recombination intermediates recognized by HU protein [52] or alterations in DNA helical twist angle patterns could be investigated.

Towards the goal of collating a complete set of *cis*-regulatory DNA sequences in *S. cerevisiae*, we performed a complementary analysis - that is, considering candidate regulatory elements not from a protein-centric viewpoint, but rather from the standpoint of putative *cis*-regulatory motifs. We collected 4,160 previously published *S. cerevisiae *DNA motifs (Additional file 13), including known TF binding site motifs and candidate regulatory motifs derived from ChIP and gene expression data (Additional file 1). Our goal was to identify 'orphan' motifs, that is, those that do not match any known TF DNA binding site motifs. We identified 34 orphan motifs (Figure S6 in Additional file 1); comparisons to all TF DNA binding site motifs in the JASPAR, TRANSFAC, and UniPROBE databases [53] (Additional file 1) did not identify significant matches to known TF DNA binding site motifs containing DBDs not yet annotated as occurring in any *S. cerevisiae *genes. Some orphan motifs might correspond to novel TFs with DBDs not yet annotated in yeast, while others might represent weak matches to known TF binding site motifs for TFs that might be utilized only in specific cellular conditions, or in the presence of particular co-factors, or in the context of a limited number of *cis *regulatory regions. Alternatively, some of the orphan motifs may represent enriched DNA sequences without a transcriptional regulatory role, or may be artifactual motifs returned by various motif discovery algorithms. Directed experimentation will be required to distinguish among these different possible scenarios.

The high-resolution nature of the *in vitro *data that we compiled in this study allowed us to perform in-depth analyses of the DNA binding specificity of TFs, resulting in novel structural and gene regulatory insights, which would not have been possible using only the motifs reported in the literature from small-scale experiments that assay binding to only a subset of potential DNA binding sequences or from ChIP experiments. Our results suggest a number of structural studies that would be interesting to pursue to investigate distinct DNA binding specificities recognized either by an individual TF or different TF family members. For example, structural studies would aid in understanding how the bZIP protein Hac1 can bind E-boxes (typical of bHLH proteins) as well as the bZIP ATF/CREB motifs [54]. Similarly, structural studies of Upc2 would provide insights on how it (and its close paralog Ecm22) recognize the sterol response element (SRE; TCGTATA) [55], whereas most other members of the fungal-specific Zn_2_Cys_6 _family recognize CG-rich binding sites primarily comprising CGG triplet half-sites separated by degenerate spacers of varying lengths [11]. It would also be interesting to determine how structurally distinct DBDs can recognize similar DNA sequences. Vhr1 and Vhr2 contain a relatively uncharacterized DBD for which no structural data are available from any species; it is not yet even known which amino acid residues in the Vhr1 DBD contact DNA. Our PBM data indicate many similarities in DNA binding specificity between the VHR class and members of the well-characterized bZIP family. Finally, the *in vivo *utilization of primary and secondary motifs for distinct biological functions by Sko1 suggests a novel gene regulatory mechanism, namely, the potential for different functions to be divided among distinct DNA binding sites in the genome for a particular TF. The extent of functionally distinct primary and secondary TF motifs would be interesting to investigate in higher eukaryotes in future studies.

In summary, this study expands our understanding of redundancy and divergence among TF family members from a structural standpoint and in terms of their regulatory functions. Moreover, this study brings us closer to, and outlines a set of priorities for, the complete characterization of TF-DNA interaction specificities in *S. cerevisiae*. The data presented here will be a valuable resource for further studies of transcriptional regulatory networks, and also for further investigations of protein-DNA recognition rules within different TF families. Such efforts in *S. cerevisiae *serve as a template for similar work aimed at cataloguing and completely characterizing TF DNA binding specificity in higher eukaryotic model organisms and in human. Ultimately, a complete compendium of human TF-DNA interaction specificity will involve cell- and tissue-specific, as well as disease-specific, interaction data that will provide invaluable details towards our understanding of development and disease.

## Materials and methods

### DNA binding specificity survey of *S. cerevisiae *TFs

Working towards the goal of obtaining high-resolution DNA binding specificities for essentially all *S. cerevisiae *TFs, we considered existing yeast TF clone collections as well as additional TFs that may have been missed or did not previously generate high-quality *in vitro *DNA binding specificity data. The proteins we examined in this study were largely derived from a collection consisting of both full-length ORF and DBD clones constructed in our prior, large-scale survey [11], plus a few additional clones either tested previously (Hap1, Stb4, Ylr278c) [10] or newly cloned by us (Ste12, Stb5, Vhr1). We selected 106 known or putative TFs that lacked high-resolution *in vitro *PBM data and 122 *S. cerevisiae *ORFs and DBDs for which we had lower confidence in their being potential sequence-specific, double-stranded DNA binding proteins; these proteins had only putative or hypothesized domains for binding double-stranded DNA, weak homology to DNA binding proteins, or literature references to potential DNA binding activity. Overall, from the combined set of 228 ORFs and DBDs, 155 were successfully cloned, expressed by *in vitro *transcription and translation (see below), and attempted on universal PBMs (Figure S7 in Additional file 1). Of these 155 proteins, we successfully obtained high-resolution DNA binding data for 27 TFs (Figure S5 in Additional file 1 and Additional file 12). Of the 128 proteins that were unsuccessful, only 38 contained known sequence-specific DBDs (bZIP, bHLH, Homeobox, Myb, zf-C2H2, zf-GATA, Zn_clus; see Conclusions).

### TF cloning and protein expression

Full-length ORFs and/or DNA binding domains were either cloned into the Gateway pDEST15 (amino-terminal GST-tag) expression vector (Invitrogen, Carlsbad, CA, USA) by recombinational cloning from previously created pENTR clones [11] or were cloned by PCR amplification from genomic DNA and Gateway cloning into pDONR221 as described previously [56] (Additional file 14). All pDEST15 clones were end-sequence verified; the source clones from which these clones were derived were previously full-length sequence verified. Nineteen genes were from a previously published, non-Gateway clone collection [10]. All proteins were produced from purified plasmids by *in vitro *transcription and translation using the PURExpress^® ^*In Vitro *Protein Synthesis Kit (New England Biolabs, Ipswich, MA, USA) according to the manufacturer's instructions. Glycerol was added to a final concentration of 38%, and proteins were stored at -20°C until further use. Western blots were performed for each protein to assess quality and to approximate protein concentration by visual inspection relative to a dilution series of a recombinant GST standard (Sigma-Aldrich, St. Louis, MO, USA), as described previously [11].

### Protein binding microarray experiments and data analysis

Custom-designed, universal 'all 10-mer' microarrays were synthesized (AMADID #015681, Agilent Technologies, Santa Clara, CA, USA) [21], converted to double-stranded DNA arrays by primer extension, and used in PBM experiments essentially as described previously [8,15]. All newly reported PBM data in this study are from experiments performed either on a fresh slide or a slide that had been stripped exactly once [21]. Microarray scanning, quantification, and data normalization were performed using masliner (MicroArray LINEar Regression) software [57] and the Universal PBM Data Analysis Suite [15] as previously described [8,15]. Determination of binding preferences for all 8-mers and derivation of associated DNA binding site PWMs were calculated using the Universal PBM Analysis Suite and the Seed-and-Wobble motif derivation algorithm [8,15]. Acceptable quality of PBM data was assessed according to visual inspection of the Cy3 and Alexa488 scans of the microarrays, the seed 8-mer from Seed-and-Wobble having an E-score of at least 0.45 [21], and obtaining at least five 8-mers with E-scores ≥0.45 matching the derived motif. These filtration criteria are based on our extensive experience with PBM data sets in this and prior studies. Graphical sequence logos were generated from the obtained PWMs using enoLOGOS [58].

### Compilation, processing, and annotation of TF DNA binding site motifs

We compiled high-resolution TF DNA binding site motifs from four studies: 1) 27 PBM-derived motifs newly generated in this study; 2) 89 PBM-derived motifs from Zhu *et al. *[11]; 3) 110 PBM-derived motifs from Badis *et al. *[10]; and 4) 28 MITOMI-derived motifs from Fordyce *et al. *[12] (see Additional file 1 for details). All 254 motifs were represented as PWMs. We trimmed all the motifs from both the 5' and 3' ends until two consecutive positions with information content ≥0.3 were reached. The motifs of TFs Cst6, Fkh1, Hcm1, Leu3, Rsc3, Ste12, Stp1, and Ydr520c were trimmed further after visual inspection. Next, we computed the AUC enrichment [13] of each motif in ChIP-chip data sets from the large-scale study of Harbison *et al. *[7]. We considered all ChIP-chip data sets with at least ten probes reported to be bound at *P *< 0.001.

For the 90 TFs examined in at least two different large-scale studies, we compared the available *in vitro *DNA binding site motifs and chose the final motifs based on the quality of the *in vitro *data, the agreement between the *in vitro *motif and previously reported motifs for the same TF, and the enrichment of the motif in *in vivo *TF binding data [7] (see Additional file 1 for details). The selected high-resolution DNA binding site motifs are available in Additional file 2 and the source of each motif is specified in Additional file 5.

Secondary motifs were computed from the PBM data using the Seed-and-Wobble algorithm, as described previously [16]. Only secondary motifs for which the 8-mer seed had an E-score > 0.48 (conservative threshold) were considered, to avoid selecting spurious secondary motifs. The selected 39 secondary motifs, trimmed as described above, are available in Additional file 2. For the comparison between *in vitro *and *in vivo *DNA binding site motifs, the *in vivo *motifs reported by MacIsaac *et al. *[20] were also trimmed, and their enrichment in the ChIP-chip data was computed as described previously [13].

### ChIP-chip data analysis using PBM data

We analyzed ChIP-chip data from Harbison *et al. *[7] essentially as described previously [13]. We use the notation *TF_cond *to refer to the ChIP-chip experiment for transcription factor *TF *under environmental condition *cond*. We scored DNA sequences using a model similar to GOMER [38], but taking into account DNA accessibility, as described previously [13]. Briefly, we use the probability that a TF ***T***binds a DNA sequence ***X ***to score every intergenic probe present on the microarrays used in the ChIP-chip experiments [7]. Using the sets of 'bound' and 'unbound' probes from each ChIP-chip experiment, and the probabilities that TF ***T ***binds each of the probes, we compute the enrichment of the PBM-derived motif for TF ***T ***in the ChIP-chip data by an AUC value. For each ChIP-chip experiment *TF_cond *we computed the AUC values of the 194 *in vitro *DNA binding motifs selected as describe above. We consider an AUC value significant if it is at least 0.65 and has an associated *P*-value ≤0.005 (that is, at most one of the 200 random motifs has an AUC value equal to or greater than the AUC value of the real motif).

### Accession IDs

PBM 8-mer data reported in this paper for 27 TFs have been deposited in the NCBI Gene Expression Omnibus (GEO) database with Platform ID GPL6796 and Series ID GSE34306.

## Abbreviations

AUC: area under the receiver operating characteristic (ROC) curve; bHLH: basic helix-loop-helix; bZIP: basic leucine zipper; ChIP: chromatin immunoprecipitation; DBD: DNA-binding domain; E-score: enrichment score; GO: Gene Ontology; MITOMI: mechanically induced trapping of molecular interactions; ORF: open reading frame; PBM: protein binding microarray; PWM: position weight matrix; TF: transcription factor; VHR: *VHT1 *regulator.

## Authors' contributions

RG conceived and performed analysis of PBM and ChIP-chip data, and structural analysis. KM performed cloning, protein expression, PBM experiments, and PBM data analysis. RPM developed software and together with KM performed analysis of functional category enrichment and gene expression data using PBM data. CZ performed cloning. AV performed protein expression and PBM experiments. MLB conceived of the study and supervised the research. KM, RG, and MLB wrote the manuscript. All authors read and approved of the manuscript.

## Supplementary Material

Additional file 1 **Detailed methods, additional figures, and additional tables**. Figure S1: ClustalW protein sequence alignment of Vhr1 and its homologs in *sensu stricto Saccharomyces *species. The alignment shows that the second putative basic region of Vhr1 is more conserved than the first basic region. Figure S2: unlike AP-1 bZIPs, Vhr1 and Vhr2 bind only to overlapping half-sites. **(a) **AP-1 bZIP transcription factors (Gcn4, Yap1, Jundm2, and the Fos-Jun heterodimer) and Vhr1 transcription factors (Vhr1 and Vhr2) bind to overlapping TGAC or TTAC half-sites. For each TF we sorted the 8-mers in decreasing order of their E-score, from 0.5 (highest affinity) to -0.5 (lowest affinity). The black lines show the 8-mers that contain TGACT (or TTACT for Yap1). **(b) **AP-1 factors (Gcn4, Yap1, Jundm2, and Fos-Jun) also bind to non-overlapping half-sites, while Vhr1 factors (Vhr1 and Vhr2) do not bind to non-overlapping half-sites. The black lines show the 8-mers that contain TGACGT (or TTACGT for Yap1). The PBM data were reported in Zhu *et al. *[11] (Gcn4, Yap1), Badis *et al. *[16] (Jundm2), Alibés *et al. *[76] (Jun-Fos), or this study (Vhr1 and Vhr2). Figure S3: comparison of the DNA binding specificities of Hac1 (both from this study and from Badis *et al. *[10]) against bHLH and bZIP TFs. **(a) **PBM-derived motifs for bZIP TF Hac1 match motifs of bHLH TFs better than motifs of bZIP TFs. **(b, c) **In-depth comparison of the DNA binding specificities of Hac1 and bHLH TF Cbf1. **(d) **In-depth comparison of the DNA binding specificities of Hac1 (this study) and two bZIP proteins that bind overlapping or adjacent TGAC half-sites: Gcn4 and Sko1, respectively. The scatter plots show the 8-mer E-scores. Figure S4: primary and secondary DNA binding site motifs derived from high-resolution *in vitro *PBM data. Figure S5: comparison of motif enrichment in ChIP-chip data for the 27 TF motifs reported in this study versus previously reported PBM-derived (Badis *et al. *[10]), ChIP-derived (MacIsaac *et al. *[20]), or MITOMI-derived (Fordyce *et al. *[12]) motifs for these 27 TFs (where available). Figure S6: *S. cerevisiae *orphan DNA binding site motifs. Figure S7: Schema of PBM experimental pipeline and results. A total of 228 ORFs/DBDs were considered in this study. Those lacking *in vitro *PBM data refers to initiation of this study in late 2008 after completion of our prior PBM survey (Zhu *et al. *[11]) and prior to publication of two more recent *in vitro *surveys (Badis *et al. *[10]; Fordyce *et al. *[12]). Table S1: TF DNA binding site motifs from the *in vitro *PBM data of Badis *et al. *[10]. Table S2: TF DNA binding site motifs from the *in vitro *MITOMI data of Fordyce *et al. *[12]. Table S3: TFs with curated high-resolution DNA binding site motifs derived from *in vitro *PBM data. The source of the selected motif (PWM) is indicated. Table S5: TFs with DNA binding site motifs reported by MacIsaac *et al. *[20] according to *in vivo *ChIP-chip data. TFs for which high-resolution *in vitro *motifs are also available are marked in boldface font. Table S8: TFs with secondary DNA binding site motifs identified from the curated set of high-resolution PBM data. Click here for file

Additional file 2 **Data file S1**. Curated set of high-resolution DNA binding site motifs (PWMs) for 150 *S. cerevisiae *TFs. The file contains 150 primary motifs and 39 secondary motifs derived from PBM data. Click here for file

Additional file 3 **Data file S2**. Curated high-resolution PBM data for 150 *S. cerevisiae *TFs, represented as E-scores for all ungapped 8-mers. These data correspond to the motifs provided in Additional file 2 (that is, the E-scores in this data file and the PWMs in Additional file 2 were generated from the same PBM experiments). Click here for file

Additional file 4 **Table S4**. Comparison of high-resolution *in vitro *DNA binding site motifs for *S. cerevisiae *TFs. Click here for file

Additional file 5 **Table S6**. Comparison of *in vivo *motifs (MacIsaac *et al. *[20]) and *in vitro *motifs (selected from this study, Zhu *et al. *[11], or Badis *et al. *[10]) for 150 *S. cerevisiae *TFs. TFs for which the *in vivo *and *in vitro *motifs are different are marked in red font. Click here for file

Additional file 6 **Table S7**. Discrepancies between *in vivo *and *in vitro *motifs for *S. cerevisiae *TFs. Click here for file

Additional file 7 **Table S9**. All over-represented functional categories of target genes for each TF examined in this study. Click here for file

Additional file 8 **Data file S3**. Gapped and ungapped 8-mers with a PBM enrichment score of at least 0.35. Click here for file

Additional file 9 **Table S10**. All significant specific conditions and condition categories from CRACR analysis for each TF examined in this study. Click here for file

Additional file 10 **Table S11**. Predicted direct and indirect TF-DNA interactions. Click here for file

Additional file 11 **Table S12**. DNA binding site motifs available for known or putative *S. cerevisiae *TFs. Click here for file

Additional file 12 **Table S13**. Categorization of remaining *S. cerevisiae *potential sequence-specific DNA binding proteins. For each of the 222 yeast proteins below, we list: the systematic name (column A); standard name (column B); structural domain found within protein (column C); designation for sequence specific DNA binding ability, either Likely, Maybe or Unlikely (column D); description of protein from the *Saccharomyces *Genome Database, including additional literature references to experimental evidence for DNA binding consensus sequences, ChIP motifs or other relevant information (column E). Criteria used for categorizing likelihood of sequence-specific DNA binding for Likely category included having a well characterized sequence-specific DNA binding domain and/or experimental evidence for sequence-specific DNA binding involving direct contact with DNA molecule (as opposed to indirect binding mediated through another protein factor). The Maybe category included proteins that contain structural domains for which instances of sequence-specific DNA binding have been demonstrated in other proteins containing that domain. Additionally, literature evidence for DNA binding ability, though not determined if sequence specific, or directly contacting DNA, was also considered. Finally, the Unlikely category contains proteins with structural domains that have failed to produce sequence-specific DNA binding *in vitro*, or have ChiP motifs likely to be through indirect interactions with DNA, or completely lack literature evidence for sequence-specific DNA binding by direct contact with DNA. Click here for file

Additional file 13 **Data file S4**. Collection of 4,160 previously published PWMs derived from *S. cerevisiae *TF-DNA binding and gene expression data. Click here for file

Additional file 14 **Table S14**. List of the 27 *S. cerevisiae *TFs that successfully yielded PBM data in this study. For each TF the table shows: (A) SGD ID; (B) common gene symbol; (C) Pfam DBD class (if known); (D) clone type (full-length ORF or DBD alone); (E) the Gateway entry clone used; (F) nucleotide sequence of cloned insert; (G) amino acid sequence of cloned insert; (H) the expected molecular weight (kDa) for the GST fusion protein expressed; (I) estimated concentration of protein used on PBM experiment, based on Western blot visual examination. All proteins were expressed by *in vitro *transcription and translation. Click here for file
