# Supplementary material for: Curated collection of yeast transcription factor DNA binding specificity data reveals novel structural and gene regulatory insights
Source: Genome Biol. 2011 Dec 21;12(12):R125. doi: 10.1186/gb-2011-12-12-r125 (PMC3334620; doi:10.1186/gb-2011-12-12-r125)
Supplement: Additional file 1 — Detailed methods, additional figures, and additional tables. Figure S1: ClustalW protein sequence alignment of Vhr1 and its homologs in sensu stricto Saccharomyces species. The alignment shows that the second putative basic region of Vhr1 is more conserved than the first basic region. Figure S2: unlike AP-1 bZIPs, Vhr1 and Vhr2 bind only to overlapping half-sites. (a) AP-1 bZIP transcription factors (Gcn4, Yap1, Jundm2, and the Fos-Jun heterodimer) and Vhr1 transcription factors (Vhr1 and Vhr2) bind to overlapping TGAC or TTAC half-sites. For each TF we sorted the 8-mers in decreasing order of their E-score, from 0.5 (highest affinity) to -0.5 (lowest affinity). The black lines show the 8-mers that contain TGACT (or TTACT for Yap1). (b) AP-1 factors (Gcn4, Yap1, Jundm2, and Fos-Jun) also bind to non-overlapping half-sites, while Vhr1 factors (Vhr1 and Vhr2) do not bind to non-overlapping half-sites. The black lines show the 8-mers that contain TGACGT (or TTACGT for Yap1). The PBM data were reported in Zhu et al. [11] (Gcn4, Yap1), Badis et al. [16] (Jundm2), Alibés et al. [76] (Jun-Fos), or this study (Vhr1 and Vhr2). Figure S3: comparison of the DNA binding specificities of Hac1 (both from this study and from Badis et al. [10]) against bHLH and bZIP TFs. (a) PBM-derived motifs for bZIP TF Hac1 match motifs of bHLH TFs better than motifs of bZIP TFs. (b, c) In-depth comparison of the DNA binding specificities of Hac1 and bHLH TF Cbf1. (d) In-depth comparison of the DNA binding specificities of Hac1 (this study) and two bZIP proteins that bind overlapping or adjacent TGAC half-sites: Gcn4 and Sko1, respectively. The scatter plots show the 8-mer E-scores. Figure S4: primary and secondary DNA binding site motifs derived from high-resolution in vitro PBM data. Figure S5: comparison of motif enrichment in ChIP-chip data for the 27 TF motifs reported in this study versus previously reported PBM-derived (Badis et al. [10]), ChIP-derived (MacIsaac et al. [20]), or MITOMI-de [file gb-2011-12-12-r125-S1.DOC]

**Additional file 1**

**Detailed methods, additional tables, and additional figures for**

“Curated collection of yeast transcription factor DNA binding specificity data

reveals novel structural and gene regulatory insights”

^1,5^Raluca Gordân, ^1,5^Kevin F. Murphy, ^1,2^Rachel Patton McCord, ^1^Cong Zhu,

^1^Anastasia Vedenko, ^1,2,3,4,6^Martha L. Bulyk

^1^Division of Genetics, Department of Medicine, Brigham and Women’s Hospital and Harvard Medical School, Boston, MA 02115, USA.

^2^Committee on Higher Degrees in Biophysics, Harvard University, Cambridge, MA 02138, USA.

^3^Department of Pathology, Brigham and Women’s Hospital and Harvard Medical School, Boston, MA 02115, USA.

^4^Harvard-MIT Division of Health Sciences and Technology, Harvard Medical School, Boston, MA 02115, USA.

^5^These authors contributed equally to this work.

^6^Corresponding author. E-mail: mlbulyk@receptor.med.harvard.edu

**Table of contents:**

1. Generating a curated resource of high-resolution DNA binding specificity data for *S. cerevisiae* TFs [2](#__RefHeading___Toc181428626)

1.1. Compiling a collection of high-resolution DNA binding data [2](#__RefHeading___Toc181428627)

1.2. Primary and secondary DNA binding site motifs [3](#__RefHeading___Toc181428628)

1.3. Discrepancies between in vitro motifs reported by different studies [3](#__RefHeading___Toc181428629)

2. Comparison of *in vitro* and *in vivo* DNA binding site motifs for *S. cerevisiae* TFs [4](#__RefHeading___Toc181428630)

3. In-depth analysis of the DNA binding specificities of bZIP and VHR TFs [5](#__RefHeading___Toc181428631)

4. Comparison of 4,160 previously published PWMs against the high-resolution *in vitro* DNA binding site motifs, and generation of the list of ‘orphan’ motifs [7](#__RefHeading___Toc181428632)

5. Scoring potential target genes of TFs using the PBM *k*-mer data [7](#__RefHeading___Toc181428633)

6. Analysis of functional category enrichment among a TF’s predicted target genes [8](#__RefHeading___Toc181428634)

7. Prediction of condition-specificity using CRACR [8](#__RefHeading___Toc181428635)

**Table S1 10**

**Table S2 13**

**Table S3 14**

**Table S5 18**

**Table S8 19**

**Figure S1 20**

**Figure S2 21**

**Figure S3 22**

**Figure S4 23**

**Figure S5 24**

**Figure S6 25**

**Figure S7 26**

1. Generating a curated resource of high-resolution DNA binding specificity data for *S. cerevisiae* TFs

1.1. Compiling a collection of high-resolution DNA binding data

We started with the high-resolution TF DNA binding data from four studies: 1) 27 PBM data sets and PBM-derived motifs from this study; 2) 89 PBM data sets and PBM-derived motifs reported by Zhu et al. and available in the UniPROBE database ; 3) 110 PBM data sets and PBM-derived motifs reported by Badis et al. ; and 4) 84 MITOMI-derived motifs for 28 TFs (i.e., 3 motifs for each TF) reported by Fordyce et al. . All motifs were represented as position weight matrices (PWMs). We trimmed all the motifs from both sides until two consecutive positions with information content ≥0.3 were reached. The motifs of TFs Cst6, Fkh1, Hcm1, Leu3, Rsc3, Ste12, Stp1, and Ydr520c were additionally trimmed after visual inspection. Next, we computed the area under a receiver operating characteristic curve (AUC) enrichment of each motif in ChIP-chip data sets from the large-scale study of Harbison et al. . We considered all 237 ChIP-chip data sets with at least 10 probes reported to be bound at a p-value < 0.001.

In this study and in Zhu et al. , we used the Seed-and-Wobble algorithm (which was specifically designed for analysis of universal PBM data ) to generate motifs from the raw PBM data. The motifs reported by Badis et al. were computed using a panel of motif discovery tools with subsequent selection of the motifs that best predicted the PBM data. In addition to using the motifs reported by Badis et al. in their Web Supplement (http://hugheslab.ccbr.utoronto.ca/supplementary-data/yeastDBD/), we ran the Seed-and-Wobble algorithm on their raw PBM data to generate an additional motif for each TF. Next, for each of the 110 TFs in , we compared the reported motif against the Seed-and-Wobble motif and chose the one that best explained the *in vivo* ChIP-chip data from Harbison et al. for that TF (i.e., the motif with the most significant AUC enrichment). Our selected source for each of the 110 chosen motifs is shown in **Additional file 1, Table S1** below. We note that Badis et al. reported DNA binding site motifs for two additional factors, Rap1 and Leu3, from DIP-chip data. These motifs did not explain the *in vivo* ChIP-chip data better than PBM-derived motifs, so we did not include them in our final curated list of high-resolution motifs.

In another recent survey, Fordyce et al. reported 84 MITOMI-derived motifs for 28 TFs (three motifs for each factor, as reported in their Web Supplement). For each TF we chose the motif that best explained the ChIP-chip data for that TF (see **Additional file 1, Table S2** for the list of motifs selected from the study of Fordyce et al. ).

Thus, we obtained a list of 254 motifs: 27 from this study, 89 from Zhu et al. , 110 from the data of Badis et al. , and 28 from Fordyce et al. . These motifs cover a total of 150 TFs, with 90 TFs being examined in at least two different studies. For these 90 TFs we compared the available high-resolution motifs using the following criteria: 1) similarity to motif(s) reported in the literature for the same TF; 2) the enrichment of the motifs in ChIP-chip data, whenever available (we considered both the AUC enrichment value and the p-value associated with the AUC, computed as described in Gordân et al. (2009) ; and 3) the quality of the PBM data, assessed based on the E-score of the seed used to compute the PWM (for PWMs computed using Seed-and-Wobble) and the number of ungapped 8-mers with E-scores above 0.49 or 0.45 (we note that E-scores above 0.45 are generally taken as a success criterion for PBM experiments ; when comparing two PBM data sets that both have numerous 8-mers with E-scores above 0.45, we use the number of 8-mers with E-score>0.49 as a measure of the quality of the PBM data). **Additional file 2,** **Data file S1** contains the selected high-resolution DNA binding site motifs. The source of each motif is specified in **Additional file 1, Table S3**.

Of the 28 motifs selected from the study of Fordyce et al. , 27 motifs are also reported in one or more PBM studies (the only exception is TF Yap7 – see below). In comparison to PBM-derived motifs (from this study, Zhu et al. , or Badis et al. ), the MITOMI-derived motifs reported by Fordyce et al. are generally shorter and more degenerate, and their enrichment in the ChIP-chip data was not better than the enrichment of corresponding motifs derived from PBM data. For this reason, we did not include any of the 27 MITOMI-derived motifs in the final set of high-resolution motifs. Furthermore, we noticed that for 6 of the 7 bZIP TFs in the study of Fordyce et al., the reported motif is the one containing adjacent TTAC or TGAC half-sites, irrespective of the half-site spacing preferences of the TF (TFs Cad1 (Yap2), Yap1 and Yap7 prefer overlapping TTAC half-sites, while Cin5 (Yap4), Sko1 and Yap3 prefer adjacent TTAC or TGAC half-sites . As mentioned above, Yap7 is the only TF analyzed by Fordyce et al. that is not present in any of the PBM studies. However, we did not add the Yap7 motif to the final list of high-resolution *in vitro* motifs because Yap7 is known to bind preferentially to overlapping TTAC half-sites , while the motif reported by Fordyce et al. contains adjacent half-sites. Furthermore, the reported Yap7 motif (TTACGTAA) does not seem to be used *in vivo*: the motif is not enriched in the Yap7 ChIP-chip data , while the overlapping half-site version of the motif (TTACTAA) is significantly enriched.

Thus, all 150 selected high-resolution motifs are in fact PBM-derived motifs. **Additional file 2, Data file S1** contains all 150 motifs in PWM format. The corresponding high-resolution PBM data for each TF (represented as E-scores for all possible ungapped 8-mers) is available in **Additional file 3, Data file S2** and in the Gene Expression Omnibus (GEO) database (Platform ID GPL6796, Series ID GSE34306).

1.2. Primary and secondary DNA binding site motifs

As observed earlier for mouse TFs , several *S. cerevisiae* TFs recognize multiple distinct DNA binding site motifs. These motifs can be identified from the high-resolution universal PBM data using the Seed-and-Wobble algorithm, as described previously . Briefly, Seed-and-Wobble first searches for a motif that captures the k-mers with high-signal intensity (including the top-scoring k-mer), also called the “primary motif”. Then, the high-scoring k-mers that are not explained well by the primary motif (if such k-mers exist) are used to build a “secondary motif” which represents the alternate binding preference of the tested TF. We identified secondary motifs for 39 of the 150 TFs in our curated TF list (**Additional file 1, Table S8; Additional file 2, Data file S1**).

1.3. Discrepancies between in vitro motifs reported by different studies

For most TFs examined here the DNA binding site motifs reported by different *in vitro* studies are in very good agreement (**Additional file 4, Table S4**). The only exceptions are the TFs Ecm22, Pdr1 and Stp4.

For TF Ecm22 there are no ChIP-chip data in Harbison et al. , so we cannot use the agreement with *in vivo* binding data as a criterion for choosing the best motif between the motifs reported in this study) (CTCGTWTAA) and by Badis et al. (TCCGGA). However, our motif matches the sterol response element previously reported to be bound by Ecm22: CTCGTATAAGC . Furthermore, TF Upc2, a close paralog of Ecm22, has the same binding site motif: CTCGTWTAA. Thus, the Ecm22 motif reported in this study was the one chosen for the curated list of high-resolution DNA binding site motifs. We note that a different PBM-derived motif for Ecm22 (CGGGnnnnCGGA) was reported in a previous study from our lab . We believe that that motif is not correct and was most likely due to cross-chamber contamination in the multiplexed PBM experiment.

Genome-wide *in vivo* TF DNA binding data are available for TF Pdr1 , so we computed the enrichment of the Pdr1 motifs reported by Zhu et al. (TTCCGGAA) and Badis et al. (CCGCGGA) in the ChIP-chip data, in an attempt to find the best motif. However, the two motifs were not significantly enriched in the ChIP-chip data, so we could not use this criterion. Next, we compared the raw PBM data reported by Zhu et al. and Badis et al. for TF Pdr1, and found that the latter were of higher quality (it contained 96 ungapped 8-mers with E-scores above 0.45, compared to only one such 8-mer for the data of Zhu et al. ). Furthermore, the Pdr1 motif of Badis et al. matches previous reported motifs for Pdr1 and Pdr3 (a closely related TF): CCGCGG and TCCGCGGA, respectively . Thus, the Pdr1 motif of Badis et al. was the one chosen for the curated list of high-resolution DNA binding site motifs.

The case of TF Stp4 is somewhat similar to that of Pdr1: DNA binding site motifs were reported by both Zhu et al. and Badis et al. , and the motifs were not enriched in the Stp4 ChIP-chip data. Both studies report high-quality PBM data for Stp4. However, according to the PBM data form Zhu et al. , Stp4 has two distinct modes of binding (i.e., both a primary and a secondary motif), and the secondary motif matches the Stp4 motif of Badis et al. . Thus, the PBM data of Zhu et al. were chosen for the curated resource of high-resolution DNA binding specificity data.

2. Comparison of *in vitro* and *in vivo* DNA binding site motifs for *S. cerevisiae* TFs

We compared the selected high-resolution *in vitro* motifs against motifs derived from the *in vivo* ChIP-chip data of Harbison et al. and MacIsaac at al. . MacIsaac et al. reported DNA binding site motifs for 124 TFs (**Additional file 1, Table S5**), available in their online supplement: http://fraenkel.mit.edu/improved_map/table/.

Of the 150 TFs with high-resolution *in vitro* motifs (listed in **Additional file 1, Table S3**), 85 TFs also have motifs derived from ChIP-chip data . We compared the *in vitro* and *in vivo* motifs for these 85 TFs (shown as logos in **Additional file 5, Table S6**) using the online version of TomTom with default settings and a database of yeast DNA binding site motifs from this study and the studies of Zhu et al. , Badis et al. , Fordyce et al. , and MacIsaac et al. . We found that for 15 of the 85 TFs (marked in red font in **Additional file 5, Table S6**) the *in vitro* high-resolution motif is different from the motif reported by MacIsaac et al. (criterion for calling two motifs similar: TomTom p-value<0.005); these TFs with discrepant motifs are: Cha4, Cst6, Fhl1, Gat3, Phd1, Rox1, Rtg3, Sfp1, Sok2, Sum1, Sut1, Uga3, Yml081w, Yox1, and Yrr1. Each of these 15 cases is discussed in detail in **Additional file 6, Table S7**, where for each TF we specify the structural class of its DNA binding domain (DBD). In the majority of these 15 cases the *in vitro* motif matches the motifs of TFs with DBDs of the same structural class, while the *in vivo* motif matches the motifs of TFs with DBDs of a different structural class. For example, the *in vitro* motif of Zn2Cys6 zinc finger TF Yrr1 matches the motifs of Zn2Cys6 TFs Yrm1 and Ykl222c, while the *in vitro* Yrr1 motif matches the motif of Reb1, a TF with a HTH Myb-type DBD. Thus, we believe the *in vitro* motifs in our curated collection reflect the direct DNA binding specificity of the TFs, while the *in vivo* motifs may reflect indirect DNA binding through a mediating TF of a different DBD class, or they may correspond to co-regulatory TFs, or they may simply be spurious motifs returned by the de novo DNA motif discovery algorithms applied to the ChIP-chip data . Each case is discussed in detail in **Additional file 6, Table S7.**

There are 39 TFs with DNA binding site motifs reported by MacIsaac et al. that do not have corresponding *in vitro* high resolution DNA binding data. Although these motifs are widely used, especially in computational studies of transcriptional regulation in *S. cerevisiae*, we caution users that some of these motifs may not reflect the DNA binding preferences of the profiled TFs. For example, MacIsaac et al. report that TF Dig1 binds the motif TGAAAC, which is a perfect match to the DNA binding site motif of another TF, Ste12. Dig1 is known to bind DNA indirectly as part of TF complexes together with Ste12 and Tec1 , so the TGAAAC motif may not reflect the direct DNA binding preference of Dig1, but rather the preference of Ste12. Similarly, Swi6 is known to bind DNA indirectly through Swi4 or Mbp1 , and the Swi6 motif reported by MacIsaac et al. (GACGCG) matches the Mbp1 motif perfectly. Another example is that of TF Met4, which does not bind DNA directly, but is recruited to specific DNA binding sites as part of complexes with TFs Cbf1, Met31, Met32 . The Met4 motif reported by MacIsaac et al. (ANCTGTG) matches the Met31/Met32 motifs. The bZIP TF Arr1 (Yap8) was reported to bind ANYTGAAT , although a detailed analysis of the DNA-contacting residues in bZIP proteins shows that Arr1 is unlikely to bind DNA because it lacks a conserved Asn in the basic region, a residue that makes essential DNA contacts (see main text).

3. In-depth analysis of the DNA binding specificities of bZIP and VHR TFs

The basic leucine zipper (bZIP) DNA-binding domain consists of 2 functionally distinct subdomains: the basic region (which makes specific DNA contacts) and the leucine zipper region (which is involved in dimerization) . Proteins of this class homo- and heterodimerize, and typically bind either overlapping or adjacent TGAC half-sites, based on which bZIPs are often categorized into two subclasses: AP-1 factors that prefer the TGA(C|G)TCA motif, and ATF/CREB factors that prefer TGACGTCA *.*

The *S. cerevisiae* genome encodes 14 bZIP factors, 8 of which belong to the fungal-specific Yap (yeast AP-1) subfamily . Unlike typical bZIPs, which prefer T**G**AC half-sites, Yap factors prefer overlapping or adjacent T**T**AC half-sites. Based on ChIP experiments, Yap1, Yap2, Yap5, Yap7, and Yap8 have been reported to bind TTACGTAA, while Yap3, Yap4, and Yap6 have been reported to bind TTA(C|G)TAA . Here, we analyze five Yap proteins – Yap1-4 and Yap6 – for which high-resolution in vitro PBM data are available either from this study or from Zhu et al. (**Figure 3A**). Of the remaining three Yap factors, Yap5 and Yap8 might not bind DNA directly: Yap5 has been reported not to bind DNA either as a homo- or heterodimer , and Yap8 contains a Leu at position 10 in the basic region, whereas the vast majority of bZIP factors contain a conserved Asn (**Figure 3B**). Furthermore, an Asn to Leu substitution at position 10 in the basic regions of *S. cerevisiae* bZIP factor Gcn4 completely abolishes binding of Gcn4 to DNA , which suggests that Yap8 might not bind DNA directly. We studied in detail the five Yap proteins (Yap1-4 and Yap6) and three additional bZIP factors (Cst6, Gcn4, and Sko1) for which high-resolution PBM data are available (this study and Zhu et al. ) (see **Figure 3A**).

In addition to bZIP proteins, we analyzed PBM data for Vhr1 and Vhr2, two TFs with previously uncharacterized DNA binding specificities. Our data indicate that these VHR proteins bind Gcn4-like motifs despite the fact that their DBD is of a different structural class. As shown in the dendrogram in **Figure 3A**, the DBDs of Vhr1 and Vhr2 are closely related to each other, but not to DBDs of bZIP proteins. Furthermore, in an alignment of the Vhr1 and Vhr2 DBDs against the basic regions of bZIP proteins (**Figure 3B**), it is apparent that essential DNA-contacting residues in the basic region of bZIPs (e.g., Asn10, Arg18) are not found in the VHR domain. We also note that VHR proteins bind exclusively to overlapping TGAC half-sites, unlike AP-1 proteins (including Gcn4), which can bind both overlapping and adjacent half-sites (**Figures 3A, 3D**). As shown in **Figure S2**, all AP-1 proteins with PBM data in UniPROBE (both homodimers and heterodimers) can also bind adjacent half-sites, unlike Vhr1 proteins. All this evidence indicates that the VHR domain is a distinct DBD structural class, despite the fact that there is significant overlap between the DNA sequences preferred by VHR and bZIP proteins.

Our results on the DNA binding specificities of bZIP proteins largely agree with what has been reported previously based on ChIP data: Yap3, Yap4 and Yap6 prefer adjacent TTAC half-sites, Yap1 and Yap2 prefer overlapping TTAC half-sites , and Gcn4 prefers overlapping TGAC half-sites . Also in agreement with previous reports , we find that AP-1 bZIP proteins (Yap1, Yap2, and Gcn4), which generally prefer overlapping half-sites, bind to adjacent half-sites with almost equal affinity: the E-scores of the 8-bp seeds for the primary and secondary DNA binding site motifs of Yap1, Yap2, and Gcn4 are very close or even identical (**Figure 3A**).

The residues that thus far have been implicated in the half-site spacing preferences of bZIPs are shown in **Figure 3B**. Residues 15, 19, and 22 are important for specifying overlapping versus adjacent half-site binding preference: Arg15, Ala19, and Leu22 are specific to AP-1 proteins, while Lys15, Ser15, Lys19, and Lys22 are specific to ATF/CREB proteins. Previous reports suggest that ATF/CREB bZIPs, which generally prefer adjacent half-sites, bind poorly to overlapping half-sites . However, our high-resolution PBM data indicate that while this is true for Cst6, Sko1, Yap4, and Yap6, the TF Yap3 can also bind overlapping TTAC half-sites with high specificity (the seed E-score for the secondary Yap3 motif is 0.493, close to that of the Yap3 primary motif seed: 0.497). This finding suggests that not all residues important for half-site spacing preference have been identified; it is possible that specific combinations of residues (not necessarily DNA-contacting residues) determine binding to overlapping versus adjacent half-sites.

Since the Yap family of bZIP proteins was first characterized , the basic region residues Gln9, Gln14, Ala16, and Phe17 (**Figures 3B, 3C**) have been reported to provide specificity for Yap-like half-sites (T**T**AC). However, we noticed that Sko1, a typical bZIP protein that binds to adjacent T**G**AC half-sites , also has a Phe at position 17 of the basic region. Our high-resolution PBM data allowed us to analyze in more detail the specificity of Sko1 for TGAC versus TTAC half-sites. As shown in **Figure 4**, Sko1 does indeed have a higher preference for TTAC half-sites than do the typical bZIP proteins Gcn4 and Cst6. This finding confirms the importance of residue Phe17 for conferring Yap-like versus Gcn4-like half-site preference.

Phe17 is the only residue that appears to be conserved between bZIP and VHR proteins (**Figure 3B**). However, we do not believe that this is significant because other essential DNA-contacting residues of bZIP proteins (at positions 10, 13, 14, and 18) are not conserved, which suggests that VHRs might not bind DNA using the region shown in this alignment. In an attempt to identify the DNA-contacting region in the VHR domain, we analyzed the protein sequence of Vhr1 and Vhr2 and found that these proteins have two putative “basic” regions (**Figure 3E**). The second basic region seems to align better to the basic regions of bZIP proteins (**Figure 3B**) than does the first basic region, and it is also more conserved across sensu stricto *Saccharomyces* species (**Figure S1**). These observations suggest that the second basic region in the VHR domain is more likely to be the one that interacts with DNA. Identifying the exact DNA-contacting residues and key specificity determinants will require further experimentation, involving mutagenesis experiments and structural studies. It would be interesting to see whether VHR proteins contact DNA in a way similar to bZIPs or if they utilize a completely novel structural mode of protein-DNA recognition.

4. Comparison of 4,160 previously published PWMs against the high-resolution *in vitro* DNA binding site motifs, and generation of the list of ‘orphan’ motifs

We compiled a large collection of 4,160 PWMs for previously published motifs, including both known TF binding site motifs and purely *in silico* derived candidate cis regulatory motifs from the literature .

Our goal was to determine whether there are any PWMs reported in these studies that do not match any of the *in vitro* high-resolution motifs (from this study, Zhu et al. , or Badis et al. ), and do not correspond to any known TF in *S. cerevisiae*. We henceforth refer to these as “orphan” motifs. For motif comparison we first used CompareACE and required a minimum similarity score of 0.7 to consider motifs as matching. Using this criterion, 775 out of the 4610 PWMs did not match any of the high-resolution *in vitro* motifs. Two authors on this paper (R.G., M.L.B.) independently visually inspected all 775 of these motifs to determine if they “look like a TF binding site motif” (that is, they have at least a few consecutive positions with reasonable information content, and they do not represent repetitive sequences). We selected a total of 89 motifs that looked like real motifs according to two independent manual inspections. Next, we tested the 89 motifs to see if parts of these motifs match parts of the high-resolution *in vitro* motifs. For this purpose we used TomTom and considered motifs as matching at a TomTom p-value < 0.005. We found 34 “orphan” motifs (**Figure S6, Data File S4**) that did not match any of the high-resolution *in vitro* motifs even according to this less stringent criterion.

5. Scoring potential target genes of TFs using the PBM *k*-mer data

The PBM *k*-mer data were used to score potential target genes in yeast. A predicted total occupancy score for a given TF was calculated for the upstream promoter region of each gene by summing the background-subtracted median PBM signal intensities for each overlapping 8-mer, considering all those 8-mers with a PBM enrichment score of at least 0.35 (**Additional file 8, Data file S3**), over the sequence up to 600 bp upstream of translation start . (If a verified ORF overlapped with this 600 bp upstream sequence, then the sequence to score was truncated at the beginning of this nearby ORF.) In this summation, we used the median intensities calculated for all sequence variants of the 8-mer pattern that produced the highest overall enrichment score, considering both contiguous 8-mers as well as 8-mers spanning up to 10 total positions, or 8-mers of the pattern 1111-gap-1111 with gaps up to 12 positions (**Additional file 8, Data file S3**). The median value of the median intensities over all 8-mers was used as a measure of the background signal intensity and was subtracted from each individual 8-mer's median signal intensity before summation.

6. Analysis of functional category enrichment among a TF’s predicted target genes

Using the median signal intensity summation method described above, we calculated a predicted total occupancy score for each gene’s promoter. We then considered the top 200 scoring genes for analysis of enrichment of Gene Ontology (GO categories) among a TF’s predicted target genes using the FuncAssociate2 online tool , with parameter settings: Mode; ordered, over, 1000 simulations, significance cutoff of 0.05. FuncAssociate2 reports raw p-values and also adjusted p-values that correspond to p-values corrected for multiple hypotheses testing by empirical resampling (see **Additional file 7, Table S9**).

We also used FuncAssociate2 to perform the GO category enrichment analysis of the genes bound *in vivo* by Sko1 that also score highly according to either the primary or the secondary Sko1 motif (see main text and **Figure 6B, 6C, and 6D**). We report the enriched GO categories at p<0.005, without Bonferroni correction because of the small number of genes in our gene sets: 30 genes that score highly according to the primary but not the secondary Sko1 motif, and 18 genes that score highly according to the secondary but not the primary Sko1 motif.

7. Prediction of condition-specificity using CRACR

We utilized our CRACR (Combination Rank-order Analysis of Condition-specific Regulation; pronounced “cracker”) algorithm to predict the condition-specific functions of *S.* *cerevisiae* TFs by integrating 1,693 publicly available microarray gene expression data sets and TF binding data from PBMs essentially as described previously . The CRACR implementation as well as detailed instructions and relevant input files are available online at:

http://the_brain.bwh.harvard.edu/CRACR/updatedCRACRinfo.html.

Briefly, CRACR searches for conditions in which genes downstream of intergenic regions exhibiting significant TF binding in PBMs are enriched among differentially expressed genes. Each gene in the yeast genome is first ranked by the predicted total occupancy of the sequence up to 600 bp upstream of its translational start site by a TF as calculated from PBM data using 8-mer median intensity summations as described previously. We then order all yeast genes according to their expression in a single condition and use a mean-centered area under a receiver operating characteristic (ROC) curve (AUC) statistical test to compare the PBM-defined ranks of similarly expressed genes within a sliding foreground window to the ranks of a background set of genes outside this window. This “area statistic” indicates whether the similarly expressed genes within each window are enriched (positive area) or depleted (negative area) for likely TF targets. The statistical significance of the maximum enrichment in an expression condition is determined by permutation testing (1,000 permutations). In addition to discovering individual conditions in which a TF is likely to be regulating its target genes, each individual expression dataset is annotated with terms describing the biological functions affected by the given experimental condition (i.e., “heat shock response” or “sporulation”) to facilitate a search for the general biological functions of a particular TF . FuncAssociate is used to calculate the enrichment of these “condition annotation terms” within a set of conditions significant for a TF using a file describing the associations between annotation terms and conditions (http://the_brain.bwh.harvard.edu/CRACR/condAssociationS1693.xls) analogous to an association file between genes and GO terms, allowing the calculation of an annotation enrichment p-value adjusted for multiple hypothesis testing and accounting for the degree of significance of each condition as measured by its maximum area statistic.

**Table S1**. TF DNA binding site motifs selected from the *in vitro* PBM data of Badis et al. (2008).

| **No.** | **TF** | **Source of PWM** | |
| --- | --- | --- | --- |
| 1 | Abf1 | From Badis et al. Web supplement, motif: | ABF1_TRH1 |
| 2 | Abf2 | From Badis et al. Web supplement, motif: | ABF2_TRH1 |
| 3 | Ace2 | From running SnW on the Badis et al. data, experiment: | ACE2_2082.1_ArrayB |
| 4 | Adr1 | From Badis et al. Web supplement, motif: | ADR1_v1_TRH1 |
| 5 | Aft2 | From Badis et al. Web supplement, motif: | AFT2_TRH1 |
| 6 | Asg1 | From Badis et al. Web supplement, motif: | ASG1_TRH1 |
| 7 | Azf1 | From Badis et al. Web supplement, motif: | AZF1_TRH1 |
| 8 | Cat8 | From Badis et al. Web supplement, motif: | CAT8_TRH1 |
| 9 | Cbf1 | From running SnW on the Badis et al. data, experiment: | CBF1_2160.2_ArrayB.2 |
| 10 | Cep3 | From Badis et al. Web supplement, motif: | CEP3_TRH1 |
| 11 | Cha4 | From Badis et al. Web supplement, motif: | CHA4_TRH1 |
| 12 | Cin5 (Yap4) | From Badis et al. Web supplement, motif: | CIN5_TRH1 |
| 13 | Crz1 | From running SnW on the Badis et al. data, experiment: | CRZ1_4531.1_ArrayB |
| 14 | Cst6 | From Badis et al. Web supplement, motif: | CST6_TRH1 |
| 15 | Cup9 | From running SnW on the Badis et al. data, experiment: | CUP9_4508.1_ArrayB |
| 16 | Dal80 | From Badis et al. Web supplement, motif: | DAL80_TRH1 |
| 17 | Dal82 | From running SnW on the Badis et al. data, experiment: | DAL82_4561.2_ArrayA |
| 18 | Dot6 (Pbf2) | From running SnW on the Badis et al. data, experiment: | DOT6_2080.1_ArrayB.1 |
| 19 | Ecm22 | From Badis et al. Web supplement, motif: | ECM22_TRH1 |
| 20 | Ecm23 | From running SnW on the Badis et al. data, experiment: | ECM23_2123.1_ArrayB |
| 21 | Eds1 (Ybr033w) | From Badis et al. Web supplement, motif: | EDS1_TRH1 |
| 22 | Fhl1 | From Badis et al. Web supplement, motif: | FHL1_TRH1 |
| 23 | Fkh2 | From Badis et al. Web supplement, motif: | FKH2_TRH1 |
| 24 | Fzf1 | From Badis et al. Web supplement, motif: | FZF1_TRH1 |
| 25 | Gat1 | From Badis et al. Web supplement, motif: | GAT1_TRH1 |
| 26 | Gat3 | From running SnW on the Badis et al. data, experiment: | GAT3_2122.5_ArrayA.2 |
| 27 | Gat4 | From Badis et al. Web supplement, motif: | GAT4_TRH1 |
| 28 | Gis1 | From running SnW on the Badis et al. data, experiment: | GIS1_4481.2_ArrayA |
| 29 | Gln3 | From running SnW on the Badis et al. data, experiment: | GLN3_2032.2_ArrayB.2 |
| 30 | Gzf3 | From running SnW on the Badis et al. data, experiment: | GZF3_2167.1_ArrayB |
| 31 | Hac1 | From Badis et al. Web supplement, motif: | HAC1_TRH1 |
| 32 | Hal9 | From Badis et al. Web supplement, motif: | HAL9_TRH1 |
| 33 | Hap1 | From Badis et al. Web supplement, motif: | HAP1_TRH1 |
| 34 | Hcm1 | From running SnW on the Badis et al. data, experiment: | HCM1_2157.3_ArrayA.2 |
| 35 | Hmra2 | From running SnW on the Badis et al. data, experiment: | HMRA2_2071.1_ArrayB |
| 36 | Hsf1 | From Badis et al. Web supplement, motif: | HSF1_TRH1 |
| 37 | Lys14 | From Badis et al. Web supplement, motif: | LYS14_TRH1 |
| 38 | Mbp1 | From running SnW on the Badis et al. data, experiment: | MBP1_4486.1_ArrayB.1 |
| 39 | Met31 | From running SnW on the Badis et al. data, experiment: | MET31_2146.1_ArrayB.2 |
| 40 | Met32 | From Badis et al. Web supplement, motif: | MET32_TRH1 |
| 41 | Mig1 | From Badis et al. Web supplement, motif: | MIG1_TRH1 |
| 42 | Mig2 | From running SnW on the Badis et al. data, experiment: | MIG2_2053.1_ArrayB |
| 43 | Mig3 | From running SnW on the Badis et al. data, experiment: | MIG3_2054.1_ArrayB |
| 44 | Msn2 | From Badis et al. Web supplement, motif: | MSN2_TRH1 |
| 45 | Msn4 | From Badis et al. Web supplement, motif: | MSN4_TRH1 |
| 46 | Nhp10 | From Badis et al. Web supplement, motif: | NHP10_TRH1 |
| 47 | Oaf1 | From Badis et al. Web supplement, motif: | OAF1_TRH1 |
| 48 | Pdr1 | From Badis et al. Web supplement, motif: | PDR1_TRH1 |
| 49 | Pdr8 | From Badis et al. Web supplement, motif: | PDR8_TRH1 |
| 50 | Phd1 | From Badis et al. Web supplement, motif: | PHD1_TRH1 |
| 51 | Pho2 | From running SnW on the Badis et al. data, experiment: | PHO2_2074.1_ArrayB.2 |
| 52 | Pho4 | From running SnW on the Badis et al. data, experiment: | PHO4_4536.1_ArrayB.2 |
| 53 | Put3 | From Badis et al. Web supplement, motif: | PUT3_TRH1 |
| 54 | Rdr1 | From Badis et al. Web supplement, motif: | RDR1_TRH1 |
| 55 | Rds1 | From running SnW on the Badis et al. data, experiment: | RDS1_2068.5_ArrayB |
| 56 | Rds2 | From Badis et al. Web supplement, motif: | RDS2_TRH1 |
| 57 | Reb1 | From Badis et al. Web supplement, motif: | REB1_TRH1_flipped |
| 58 | Rei1 | From Badis et al. Web supplement, motif: | REI1_TRH1 |
| 59 | Rfx1 | From Badis et al. Web supplement, motif: | RFX1_TRH1 |
| 60 | Rgm1 | From running SnW on the Badis et al. data, experiment: | RGM1_2105.1_ArrayB |
| 61 | Rgt1 | From Badis et al. Web supplement, motif: | RGT1_TRH1 |
| 62 | Rim101 | From Badis et al. Web supplement, motif: | RIM101_TRH1 |
| 63 | Rox1 | From Badis et al. Web supplement, motif: | ROX1_TRH1 |
| 64 | Rph1 | From running SnW on the Badis et al. data, experiment: | RPH1_4571.1_ArrayB |
| 65 | Rpn4 | From Badis et al. Web supplement, motif: | RPN4_TRH1 |
| 66 | Rsc3 | From running SnW on the Badis et al. data, experiment: | RSC3_2035.2_ArrayA |
| 67 | Rsc30 | From Badis et al. Web supplement, motif: | RSC30_TRH1 |
| 68 | Sig1 | From Badis et al. Web supplement, motif: | SIG1_TRH1 |
| 69 | Sip4 | From Badis et al. Web supplement, motif: | SIP4_TRH1 |
| 70 | Skn7 | From running SnW on the Badis et al. data, experiment: | SKN7_2117.1_ArrayB.1 |
| 71 | Sok2 | From running SnW on the Badis et al. data, experiment: | SOK2_4560.1_ArrayA |
| 72 | Srd1 | From Badis et al. Web supplement, motif: | SRD1_TRH1 |
| 73 | Stb4 | From Badis et al. Web supplement, motif: | STB4_TRH1 |
| 74 | Stb5 | From Badis et al. Web supplement, motif: | STB5_TRH1 |
| 75 | Ste12 | From Badis et al. Web supplement, motif: | STE12_TRH1 |
| 76 | Stp3 | From running SnW on the Badis et al. data, experiment: | STP3_4483.2_ArrayA |
| 77 | Stp4 | From running SnW on the Badis et al. data, experiment: | STP4_2135.1_ArrayB |
| 78 | Sum1 | From Badis et al. Web supplement, motif: | SUM1_TRH1 |
| 79 | Swi4 | From Badis et al. Web supplement, motif: | SWI4_TRH1 |
| 80 | Swi5 | From Badis et al. Web supplement, motif: | SWI5_TRH1 |
| 81 | Tbf1 | From running SnW on the Badis et al. data, experiment: | TBF1_2078.1_ArrayB |
| 82 | Tbs1 | From Badis et al. Web supplement, motif: | TBS1_TRH1 |
| 83 | Tea1 | From Badis et al. Web supplement, motif: | TEA1_TRH1 |
| 84 | Tec1 | From Badis et al. Web supplement, motif: | TEC1_TRH1 |
| 85 | Tos8 | From Badis et al. Web supplement, motif: | TOS8_TRH1 |
| 86 | Tye7 | From Badis et al. Web supplement, motif: | TYE7_TRH1 |
| 87 | Uga3 | From Badis et al. Web supplement, motif: | UGA3_TRH1 |
| 88 | Ume6 | From Badis et al. Web supplement, motif: | UME6_TRH1 |
| 89 | Xbp1 | From Badis et al. Web supplement, motif: | XBP1_TRH1 |
| 90 | Yap3 | From running SnW on the Badis et al. data, experiment: | YAP3_4535.1_ArrayB.2 |
| 91 | Ybl054w (Pbf1) | From Badis et al. Web supplement, motif: | YBL054W_TRH1 |
| 92 | Ybr239c | From Badis et al. Web supplement, motif: | YBR239C_TRH1 |
| 93 | Ydr520c | From running SnW on the Badis et al. data, experiment: | YDR520c_2043.2_ArrayA |
| 94 | Yer130c | From running SnW on the Badis et al. data, experiment: | YER130C_2103.2_ArrayA |
| 95 | Yer184c | From Badis et al. Web supplement, motif: | YER184C_TRH1 |
| 96 | Ygr067c | From running SnW on the Badis et al. data, experiment: | YGR067C_2151.1_ArrayB |
| 97 | Yjl103c (Gsm1) | From Badis et al. Web supplement, motif: | YJL103C_TRH1 |
| 98 | Ykl222c | From Badis et al. Web supplement, motif: | YKL222C_TRH1 |
| 99 | Yll054c | From Badis et al. Web supplement, motif: | YLL054C_TRH1 |
| 100 | Ylr278c | From Badis et al. Web supplement, motif: | YLR278C_TRH1 |
| 101 | Yml081w | From running SnW on the Badis et al. data, experiment: | YML081W_2152.1_ArrayB |
| 102 | Ynr063w | From Badis et al. Web supplement, motif: | YNR063W_TRH1 |
| 103 | Yox1 | From Badis et al. Web supplement, motif: | YOX1_TRH1 |
| 104 | Ypl230w (Usv1) | From running SnW on the Badis et al. data, experiment: | YPL230W_2051.2_ArrayB |
| 105 | Ypr013c | From running SnW on the Badis et al. data, experiment: | YPR013C_2104.1_ArrayB |
| 106 | Ypr022c | From Badis et al. Web supplement, motif: | YPR022C_TRH1 |
| 107 | Ypr196w | From running SnW on the Badis et al. data, experiment: | YPR196W_2047.3_ArrayA.2 |
| 108 | Yrm1 | From Badis et al. Web supplement, motif: | YRM1_TRH1 |
| 109 | Yrr1 | From running SnW on the Badis et al. data, experiment: | YRR1_2114.2_ArrayA |
| 110 | Zms1 | From running SnW on the Badis et al. data, experiment: | ZMS1_4493.1_ArrayA |

**Table S2.** TF DNA binding site motifs selected from the *in vitro* MITOMI data of Fordyce et al. (2010).

| **No.** | **TF** | **Source of PWM** | |
| --- | --- | --- | --- |
| 1 | Ace2 | From Fordyce et al. web supplement, motif: | Ace2_NGCTGGYH |
| 2 | Aft1 (Rcs1) | From Fordyce et al. web supplement, motif: | Aft1_RGGTGYR |
| 3 | Aft2 | From Fordyce et al. web supplement, motif: | Aft2_NGGGTGTD |
| 4 | Bas1 | From Fordyce et al. web supplement, motif: | Bas1_NACTCTKT |
| 5 | Cad1 (Yap2) | From Fordyce et al. web supplement, motif: | Cad1_TTABGTAA |
| 6 | Cbf1 | From Fordyce et al. web supplement, motif: | Cbf1_VCACGTG |
| 7 | Cin5 (Yap4) | From Fordyce et al. web supplement, motif: | Cin5_TTABGTVA |
| 8 | Cup9 | From Fordyce et al. web supplement, motif: | Cup9_TGACGTVN |
| 9 | Dal80 | From Fordyce et al. web supplement, motif: | Dal80_SWTATCGN |
| 10 | Gat1 | From Fordyce et al. web supplement, motif: | Gat1_KCVMGATA |
| 11 | Gcn4 | From Fordyce et al. web supplement, motif: | Gcn4_TGASTCAN |
| 12 | Mata2 | From Fordyce et al. web supplement, motif: | Mata2_NDCRTGTH |
| 13 | Mcm1 | From Fordyce et al. web supplement, motif: | Mcm1_WTTACTAA |
| 14 | Met31 | From Fordyce et al. web supplement, motif: | Met31_CRCWGTRN |
| 15 | Met32 | From Fordyce et al. web supplement, motif: | Met32_NVCACAVT |
| 16 | Msn1 | From Fordyce et al. web supplement, motif: | Msn1_GGACATDN |
| 17 | Msn2 | From Fordyce et al. web supplement, motif: | Msn2_NVYCCYT |
| 18 | Nrg2 | From Fordyce et al. web supplement, motif: | Nrg2_HAGGGTCN |
| 19 | Pdr3 | From Fordyce et al. web supplement, motif: | Pdr3_NVVCGGA |
| 20 | Pho4 | From Fordyce et al. web supplement, motif: | Pho4_NCACGTGD |
| 21 | Reb1 | From Fordyce et al. web supplement, motif: | Reb1_NTTACCCK |
| 22 | Rox1 | From Fordyce et al. web supplement, motif: | Rox1_AVVACAAT |
| 23 | Rpn4 | From Fordyce et al. web supplement, motif: | Rpn4_RGTGGCDN |
| 24 | Sko1 | From Fordyce et al. web supplement, motif: | Sko1_ATKACGTN |
| 25 | Stb5 | From Fordyce et al. web supplement, motif: | Stb5_TMDCWCCG |
| 26 | Yap1 | From Fordyce et al. web supplement, motif: | Yap1_TYACGKAA |
| 27 | Yap3 | From Fordyce et al. web supplement, motif: | Yap3_TTAHSTAA |
| 28 | Yap7 | From Fordyce et al. web supplement, motif: | Yap7_NAWTTACG |

**Table S3.** TFs with curated high-resolution DNA binding site motifs derived from *in vitro* PBM data. The source of the selected motif (PWM) is indicated. “SnW” refers to the Seed-and-Wobble motif derivation algorithm (Berger et al., Nat. Biotech., 2006; Berger and Bulyk, Nat. Protocols, 2009).

| **No.** | **TF** | **Source of PWM** | |
| --- | --- | --- | --- |
| 1 | Abf1 | This study, motif: | Abf1 |
| 2 | Abf2 | From Badis et al. Web supplement, motif: | ABF2_TRH1 |
| 3 | Ace2 | From running SnW on the Badis et al. data, experiment: | ACE2_2082.1_ArrayB |
| 4 | Adr1 | From Badis et al. Web supplement, motif: | ADR1_v1_TRH1 |
| 5 | Aft1 (Rcs1) | From Zhu et al. (UniPROBE), motif: | Aft1 |
| 6 | Aft2 | From Badis et al. Web supplement, motif: | AFT2_TRH1 |
| 7 | Aro80 | From Zhu et al. (UniPROBE), motif: | Aro80 |
| 8 | Asg1 | From Zhu et al. (UniPROBE), motif: | Asg1 |
| 9 | Azf1 | From Badis et al. Web supplement, motif: | AZF1_TRH1 |
| 10 | Bas1 | From Zhu et al. (UniPROBE), motif: | Bas1 |
| 11 | Cad1 (Yap2) | This study, motif: | Cad1 |
| 12 | Cat8 | From Badis et al. Web supplement, motif: | CAT8_TRH1 |
| 13 | Cbf1 | From Zhu et al. (UniPROBE), motif: | Cbf1 |
| 14 | Cep3 | From Badis et al. Web supplement, motif: | CEP3_TRH1 |
| 15 | Cha4 | From Zhu et al. (UniPROBE), motif: | Cha4 |
| 16 | Cin5 (Yap4) | This study, motif: | Cin5 |
| 17 | Crz1 | From running SnW on the Badis et al. data, experiment: | CRZ1_4531.1_ArrayB |
| 18 | Cst6 | This study, motif: | Cst6 |
| 19 | Cup9 | From running SnW on the Badis et al. data, experiment: | CUP9_4508.1_ArrayB |
| 20 | Dal80 | From Badis et al. Web supplement, motif: | DAL80_TRH1 |
| 21 | Dal82 | From running SnW on the Badis et al. data, experiment: | DAL82_4561.2_ArrayA |
| 22 | Ecm22 | This study, motif: | Ecm22 |
| 23 | Ecm23 | From running SnW on the Badis et al. data, experiment: | ECM23_2123.1_ArrayB |
| 24 | Eds1 (Ybr033w) | This study, motif: | Ybr033w |
| 25 | Fhl1 | From Zhu et al. (UniPROBE), motif: | Fhl1 |
| 26 | Fkh1 | From Zhu et al. (UniPROBE), motif: | Fkh1 |
| 27 | Fkh2 | From Zhu et al. (UniPROBE), motif: | Fkh2 |
| 28 | Fzf1 | From Badis et al. Web supplement, motif: | FZF1_TRH1 |
| 29 | Gal4 | From Zhu et al. (UniPROBE), motif: | Gal4 |
| 30 | Gat1 | From Zhu et al. (UniPROBE), motif: | Gat1 |
| 31 | Gat3 | From Zhu et al. (UniPROBE), motif: | Gat3 |
| 32 | Gat4 | From Zhu et al. (UniPROBE), motif: | Gat4 |
| 33 | Gcn4 | From Zhu et al. (UniPROBE), motif: | Gcn4 |
| 34 | Gcr1 | This study, motif: | Gcr1 |
| 35 | Gis1 | From running SnW on the Badis et al. data, experiment: | GIS1_4481.2_ArrayA |
| 36 | Gln3 | From running SnW on the Badis et al. data, experiment: | GLN3_2032.2_ArrayB.2 |
| 37 | Gsm1 (Yjl103c) | From Zhu et al. (UniPROBE), motif: | Gsm1 |
| 38 | Gzf3 | From Zhu et al. (UniPROBE), motif: | Gzf3 |
| 39 | Hac1[[1]](#footnote-2)* | From Badis et al. Web supplement, motif: | HAC1_TRH1 |
| 40 | Hal9 | From Zhu et al. (UniPROBE), motif: | Hal9 |
| 41 | Hap1 | This study, motif: | Hap1 |
| 42 | Hcm1 | From running SnW on the Badis et al. data, experiment: | HCM1_2157.3_ArrayA.2 |
| 43 | Hmlalpha2 | This study, motif: | Hmlalpha2 |
| 44 | Hmra2 | From running SnW on the Badis et al. data, experiment: | HMRA2_2071.1_ArrayB |
| 45 | Hsf1 | From Badis et al. Web supplement, motif: | HSF1_TRH1 |
| 46 | Leu3 | From Zhu et al. (UniPROBE), motif: | Leu3 |
| 47 | Lys14 | From Zhu et al. (UniPROBE), motif: | Lys14 |
| 48 | Matalpha2 | From Zhu et al. (UniPROBE), motif: | Matalpha2 |
| 49 | Mbp1 | From Zhu et al. (UniPROBE), motif: | Mbp1 |
| 50 | Mcm1 | From Zhu et al. (UniPROBE), motif: | Mcm1 |
| 51 | Met31 | From running SnW on the Badis et al. data, experiment: | MET31_2146.1_ArrayB.2 |
| 52 | Met32 | From Badis et al. Web supplement, motif: | MET32_TRH1 |
| 53 | Mga1 | From Zhu et al. (UniPROBE), motif: | Mga1 |
| 54 | Mig1 | From Zhu et al. (UniPROBE), motif: | Mig1 |
| 55 | Mig2 | From Zhu et al. (UniPROBE), motif: | Mig2 |
| 56 | Mig3 | From Zhu et al. (UniPROBE), motif: | Mig3 |
| 57 | Mot3 | This study, motif: | Mot3 |
| 58 | Msn1 | This study, motif: | Msn1 |
| 59 | Msn2 | From Badis et al. Web supplement, motif: | MSN2_TRH1 |
| 60 | Msn4 | From Badis et al. Web supplement, motif: | MSN4_TRH1 |
| 61 | Ndt80 | From Zhu et al. (UniPROBE), motif: | Ndt80 |
| 62 | Nhp10 | From Badis et al. Web supplement, motif: | NHP10_TRH1 |
| 63 | Nhp6a | From Zhu et al. (UniPROBE), motif: | Nhp6a |
| 64 | Nhp6b | From Zhu et al. (UniPROBE), motif: | Nhp6b |
| 65 | Nrg1 | From Zhu et al. (UniPROBE), motif: | Nrg1 |
| 66 | Nrg2 | This study, motif: | Nrg2 |
| 67 | Oaf1 | From Badis et al. Web supplement, motif: | OAF1_TRH1 |
| 68 | Pbf1 (Ybl054w) | From Zhu et al. (UniPROBE), motif: | Pbf1 |
| 69 | Pbf2 (Dot6) | From Zhu et al. (UniPROBE), motif: | Pbf2 |
| 70 | Pdr1 | From Badis et al. Web supplement, motif: | PDR1_TRH1 |
| 71 | Pdr3 | This study, motif: | Pdr3 |
| 72 | Pdr8 | From Badis et al. Web supplement, motif: | PDR8_TRH1 |
| 73 | Phd1 | From Zhu et al. (UniPROBE), motif: | Phd1 |
| 74 | Pho2 | From running SnW on the Badis et al. data, experiment: | PHO2_2074.1_ArrayB.2 |
| 75 | Pho4 | From Zhu et al. (UniPROBE), motif: | Pho4 |
| 76 | Put3 | From Badis et al. Web supplement, motif: | PUT3_TRH1 |
| 77 | Rap1 | From Zhu et al. (UniPROBE), motif: | Rap1 |
| 78 | Rdr1 | From Zhu et al. (UniPROBE), motif: | Rdr1 |
| 79 | Rds1 | From Zhu et al. (UniPROBE), motif: | Rds1 |
| 80 | Rds2 | From Badis et al. Web supplement, motif: | RDS2_TRH1 |
| 81 | Reb1 | From Badis et al. Web supplement, motif: | REB1_TRH1_flipped |
| 82 | Rei1 | From Badis et al. Web supplement, motif: | REI1_TRH1 |
| 83 | Rfx1 | From Badis et al. Web supplement, motif: | RFX1_TRH1 |
| 84 | Rgm1 | From running SnW on the Badis et al. data, experiment: | RGM1_2105.1_ArrayB |
| 85 | Rgt1 | From Badis et al. Web supplement, motif: | RGT1_TRH1 |
| 86 | Rim101 | From Badis et al. Web supplement, motif: | RIM101_TRH1 |
| 87 | Rox1 | From Badis et al. Web supplement, motif: | ROX1_TRH1 |
| 88 | Rph1 | From running SnW on the Badis et al. data, experiment: | RPH1_4571.1_ArrayB |
| 89 | Rpn4 | From Badis et al. Web supplement, motif: | RPN4_TRH1 |
| 90 | Rsc3 | From Zhu et al. (UniPROBE), motif: | Rsc3 |
| 91 | Rsc30 | From Zhu et al. (UniPROBE), motif: | Rsc30 |
| 92 | Rtg3 | From Zhu et al. (UniPROBE), motif: | Rtg3 |
| 93 | Sfl1 | From Zhu et al. (UniPROBE), motif: | Sfl1 |
| 94 | Sfp1 | From Zhu et al. (UniPROBE), motif: | Sfp1 |
| 95 | Sig1 | From Badis et al. Web supplement, motif: | SIG1_TRH1 |
| 96 | Sip4 | From Badis et al. Web supplement, motif: | SIP4_TRH1 |
| 97 | Skn7 | From running SnW on the Badis et al. data, experiment: | SKN7_2117.1_ArrayB.1 |
| 98 | Sko1 | This study, motif: | Sko1 |
| 99 | Smp1 | From Zhu et al. (UniPROBE), motif: | Smp1 |
| 100 | Sok2 | From running SnW on the Badis et al. data, experiment: | SOK2_4560.1_ArrayA |
| 101 | Spt15 | From Zhu et al. (UniPROBE), motif: | Spt15 |
| 102 | Srd1 | From Badis et al. Web supplement, motif: | SRD1_TRH1 |
| 103 | Stb3 | From Zhu et al. (UniPROBE), motif: | Stb3 |
| 104 | Stb4 | This study, motif: | Stb4 |
| 105 | Stb5 | This study, motif: | Stb5 |
| 106 | Ste12 | This study, motif: | Ste12 |
| 107 | Stp1 | This study, motif: | Stp1 |
| 108 | Stp2 | From Zhu et al. (UniPROBE), motif: | Stp2 |
| 109 | Stp3 | From running SnW on the Badis et al. data, experiment: | STP3_4483.2_ArrayA |
| 110 | Stp4 | From Zhu et al. (UniPROBE), motif: | Stp4 |
| 111 | Sum1 | From Zhu et al. (UniPROBE), motif: | Sum1 |
| 112 | Sut1 | This study, motif: | Sut1 |
| 113 | Sut2 | From Zhu et al. (UniPROBE), motif: | Sut2 |
| 114 | Swi4 | From Badis et al. Web supplement, motif: | SWI4_TRH1 |
| 115 | Swi5 | From Badis et al. Web supplement, motif: | SWI5_TRH1 |
| 116 | Tbf1 | From Zhu et al. (UniPROBE), motif: | Tbf1 |
| 117 | Tbs1 | From Zhu et al. (UniPROBE), motif: | Tbs1 |
| 118 | Tea1 | From Zhu et al. (UniPROBE), motif: | Tea1 |
| 119 | Tec1 | From Badis et al. Web supplement, motif: | TEC1_TRH1 |
| 120 | Tos8 | From Badis et al. Web supplement, motif: | TOS8_TRH1 |
| 121 | Tye7 | From Zhu et al. (UniPROBE), motif: | Tye7 |
| 122 | Uga3 | From Badis et al. Web supplement, motif: | UGA3_TRH1 |
| 123 | Ume6 | From Zhu et al. (UniPROBE), motif: | Ume6 |
| 124 | Upc2 | This study, motif: | Upc2 |
| 125 | Usv1 (Ypl230w) | From Zhu et al. (UniPROBE), motif: | Usv1 |
| 126 | Vhr1 | This study, motif: | Vhr1 |
| 127 | Xbp1 | From Badis et al. Web supplement, motif: | XBP1_TRH1 |
| 128 | Yap1 | From Zhu et al. (UniPROBE), motif: | Yap1 |
| 129 | Yap3 | This study, motif: | Yap3 |
| 130 | Yap6 | From Zhu et al. (UniPROBE), motif: | Yap6 |
| 131 | Ybr239c | From Zhu et al. (UniPROBE), motif: | Ybr239c |
| 132 | Ydr520c | From running SnW on the Badis et al. data, experiment: | YDR520c_2043.2_ArrayA |
| 133 | Yer064c | This study, motif: | Yer064c |
| 134 | Yer130c | From running SnW on the Badis et al. data, experiment: | YER130C_2103.2_ArrayA |
| 135 | Yer184c | This study, motif: | Yer184c |
| 136 | Ygr067c | From running SnW on the Badis et al. data, experiment: | YGR067C_2151.1_ArrayB |
| 137 | Ykl222c | From Zhu et al. (UniPROBE), motif: | Ykl222c |
| 138 | Yll054c | From Zhu et al. (UniPROBE), motif: | Yll054c |
| 139 | Ylr278c | This study, motif: | Ylr278c |
| 140 | Yml081w | From Zhu et al. (UniPROBE), motif: | Yml081w |
| 141 | Ynr063w | From Badis et al. Web supplement, motif: | YNR063W_TRH1 |
| 142 | Yox1 | From Badis et al. Web supplement, motif: | YOX1_TRH1 |
| 143 | Ypr013c | From Zhu et al. (UniPROBE), motif: | Ypr013c |
| 144 | Ypr015c | From Zhu et al. (UniPROBE), motif: | Ypr015c |
| 145 | Ypr022c | From Badis et al. Web supplement, motif: | YPR022C_TRH1 |
| 146 | Ypr196w | From running SnW on the Badis et al. data, experiment: | YPR196W_2047.3_ArrayA.2 |
| 147 | Yrm1 | From Badis et al. Web supplement, motif: | YRM1_TRH1 |
| 148 | Yrr1 | From Zhu et al. (UniPROBE), motif: | Yrr1 |
| 149 | Zap1 | This study, motif: | Zap1 |
| 150 | Zms1 | From running SnW on the Badis et al. data, experiment: | ZMS1_4493.1_ArrayA |

**Table S5.** TFs with DNA binding site motifs reported by MacIsaac et al. (2006) according to *in vivo* ChIP-chip data. TFs for which high-resolution *in vitro* motifs are also available are marked in boldface font.

| **Abf1** | **Ecm22** | Ime1 | **Pdr3** | **Sip4** | **Tec1** |
| --- | --- | --- | --- | --- | --- |
| **Ace2** | **Fhl1** | Ino2 | **Phd1** | **Skn7** | Thi2 |
| **Adr1** | **Fkh1** | Ino4 | **Pho2** | **Sko1** | **Tye7** |
| **Aft2** | **Fkh2** | Ixr1 | **Pho4** | **Smp1** | **Uga3** |
| Arg80 | **Gal4** | **Leu3** | **Put3** | Snf1 | **Ume6** |
| Arg81 | Gal80 | Mac1 | **Rap1** | Snt2 | **Xbp1** |
| **Aro80** | **Gat1** | Mata1 | **Rcs1 (Aft1)** | **Sok2** | **Yap1** |
| Arr1 (Yap8) | **Gat3** | **Mbp1** | **Rds1** | Spt2 | **Yap3** |
| Ash1 | **Gcn4** | **Mcm1** | **Reb1** | Spt23 | Yap5 |
| **Azf1** | **Gcr1** | Met28 | **Rfx1** | Stb1 | **Yap6** |
| **Bas1** | Gcr2 | **Met31** | **Rgt1** | Stb2 | Yap7 |
| **Cad1 (Yap2)** | **Gln3** | **Met32** | **Rim101** | **Stb4** | Ydr026c |
| **Cbf1** | Gts1 | Met4 | Rlm1 | **Stb5** | **Ydr520c** |
| **Cha4** | **Gzf3** | **Mig1** | Rlr1 | **Ste12** | Yer051w |
| **Cin5 (Yap4)** | **Hac1** | **Mot3** | Rme1 | **Stp1** | Yhp1 |
| **Crz1** | **Hap1** | **Msn2** | **Rox1** | **Stp4** | **Yml081w** |
| **Cst6** | Hap2 | **Msn4** | **Rph1** | **Sum1** | **Yox1** |
| **Dal80** | Hap3 | Ndd1 | **Rpn4** | **Sut1** | **Yrr1** |
| Dal81 | Hap4 | **Nrg1** | **Rtg3** | **Swi4** | **Zap1** |
| **Dal82** | Hap5 | Opi1 | **Sfl1** | **Swi5** |  |
| Dig1 | **Hsf1** | **Pdr1** | **Sfp1** | Swi6 |  |

**Table S8.** TFs with secondary DNA binding site motifs identified from the curated set of high-resolution PBM data.

| No. | TF | Source |
| --- | --- | --- |
| 1 | Cad1 (Yap2) | This study. |
| 2 | Cst6 | This study. |
| 3 | Ecm22 | This study. |
| 4 | Gat1 | From Zhu et al. |
| 5 | Gat3 | From Zhu et al. |
| 6 | Gcn4 | From Zhu et al. |
| 7 | Hap1 | This study. |
| 8 | Hmlalpha2 | This study. |
| 9 | Leu3 | From Zhu et al. |
| 10 | Lys14 | From Zhu et al. |
| 11 | Mga1 | From Zhu et al. |
| 12 | Mig2 | From Zhu et al. |
| 13 | Mot3 | This study. |
| 14 | Msn1 | This study. |
| 15 | Pbf1 | From Zhu et al. |
| 16 | Pbf2 | From Zhu et al. |
| 17 | Pdr3 | This study. |
| 18 | Pho4 | From Zhu et al. |
| 19 | Rds1 | From Zhu et al. |
| 20 | Rgm1 | From running SnW on the Badis et al. data, experiment RGM1_2105.1_ArrayB |
| 21 | Sko1 | This study. |
| 22 | Stb3 | From Zhu et al. |
| 23 | Stb4 | This study. |
| 24 | Stb5 | This study. |
| 25 | Stp4 | From Zhu et al. |
| 26 | Tbf1 | From Zhu et al. |
| 27 | Tbs1 | From Zhu et al. |
| 28 | Tea1 | From Zhu et al. |
| 29 | Tye7 | From Zhu et al. |
| 30 | Ume6 | From Zhu et al. |
| 31 | Upc2 | This study. |
| 32 | Usv1 | From Zhu et al. |
| 33 | Yap1 | From Zhu et al. |
| 34 | Yap3 | This study. |
| 35 | Ybr239c | From Zhu et al. |
| 36 | Yer064c | This study. |
| 37 | Ylr278c | This study. |
| 38 | Yml081w | From Zhu et al. |
| 39 | Zap1 | This study. |

-
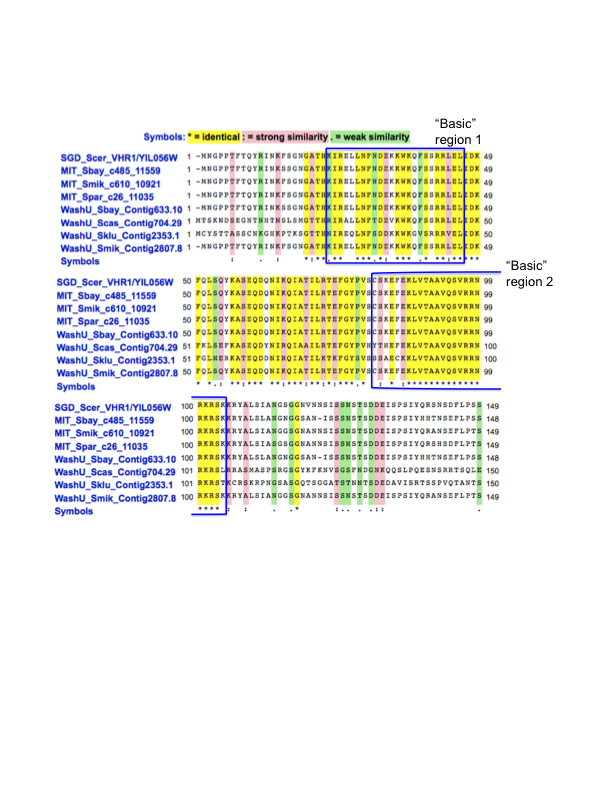

- **Figure S1.** ClustalW protein sequence alignment of Vhr1 and its homologs in *sensu stricto* *Saccharomyces* species. The alignment shows that the second putative basic region of Vhr1 is more conserved than the first basic region.


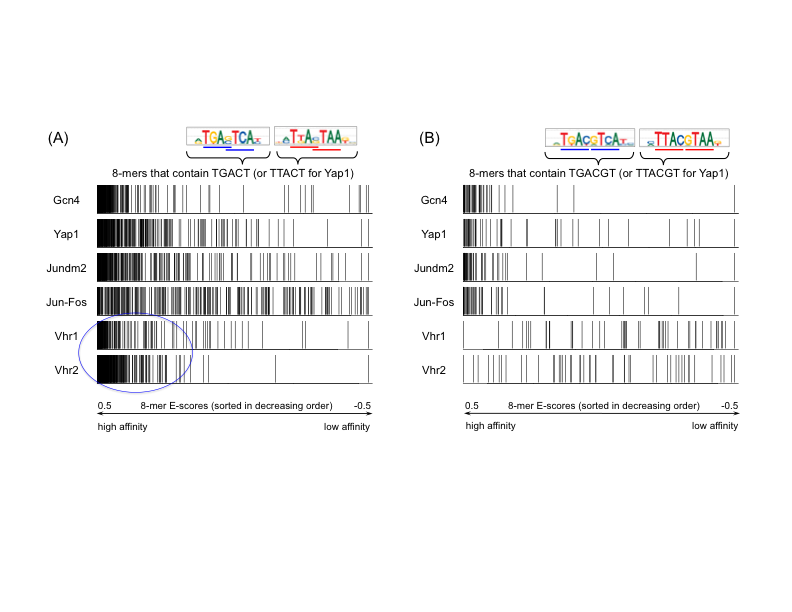


**Figure S2.** Unlike AP-1 bZIPs, Vhr1 and Vhr2 bind only to overlapping half-sites. (A) AP-1 bZIP transcription factors (Gcn4, Yap1, Jundm2, and the Fos-Jun heterodimer) and Vhr1 transcription factors (Vhr1 and Vhr2) bind to overlapping TGAC or TTAC half-sites. For each TF we sorted the 8-mers in decreasing order of their E-score, from 0.5 (highest affinity) to -0.5 (lowest affinity). The black lines show the 8-mers that contain TGACT (or TTACT for Yap1). (B) AP-1 factors (Gcn4, Yap1, Jundm2, and Fos-Jun) also bind to non-overlapping half-sites, while Vhr1 factors (Vhr1 and Vhr2) do not bind to non-overlapping half-sites. The black lines show the 8-mers that contain TGACGT (or TTACGT for Yap1). The PBM data was reported in Zhu et al. 2009 (Gcn4, Yap1), Badis et al. 2009 (Jundm2), Alibes et al. 2010 (Jun-Fos), or this study (Vhr1 and Vhr2).


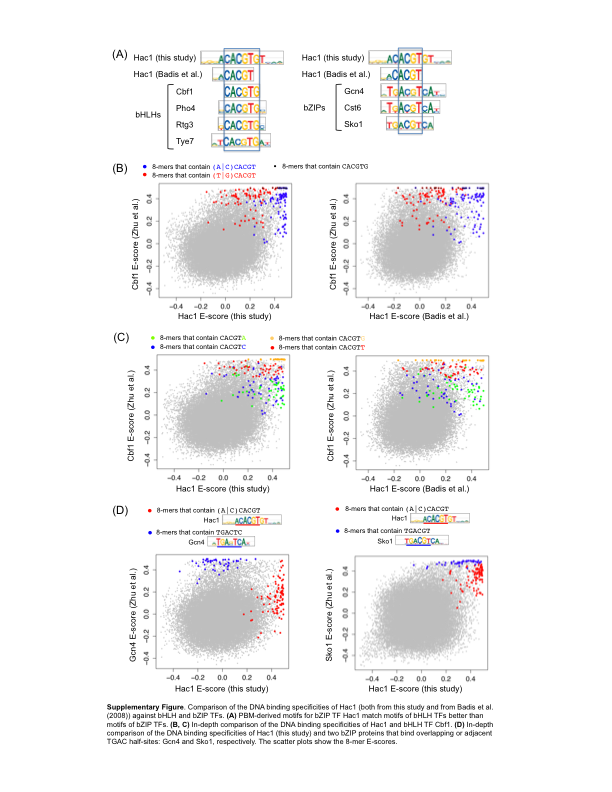


- **Figure S3.** Comparison of the DNA binding specificities of Hac1 (both from this study and from Badis et al. (2008)) against bHLH and bZIP TFs. **(A)** PBM-derived motifs for bZIP TF Hac1 match motifs of bHLH TFs better than motifs of bZIP TFs. **(B, C)** In-depth comparison of the DNA binding specificities of Hac1 and bHLH TF Cbf1. **(D)** In-depth comparison of the DNA binding specificities of Hac1 (this study) and two bZIP proteins that bind overlapping or adjacent TGAC half-sites: Gcn4 and Sko1, respectively. The scatter plots show the 8-mer E-scores.
-
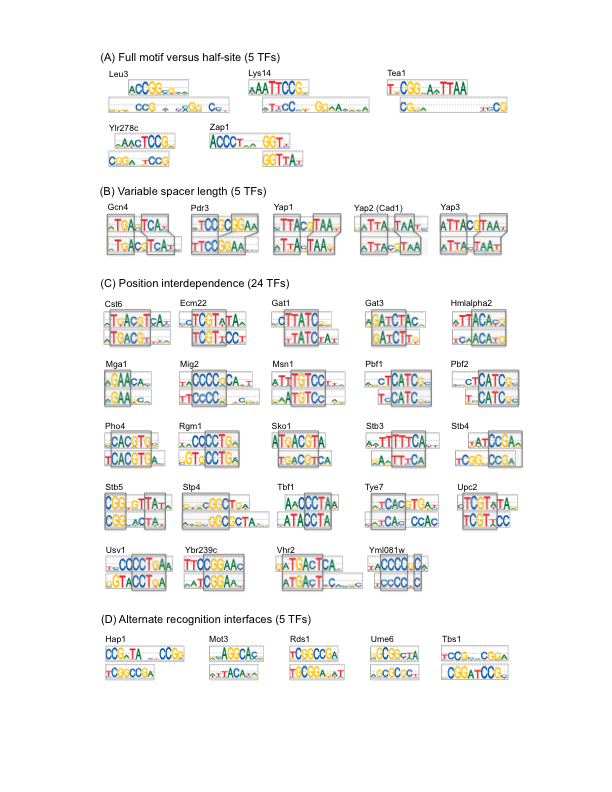

- **Figure S4.** Primary and secondary DNA binding site motifs derived from high-resolution *in vitro* PBM data.


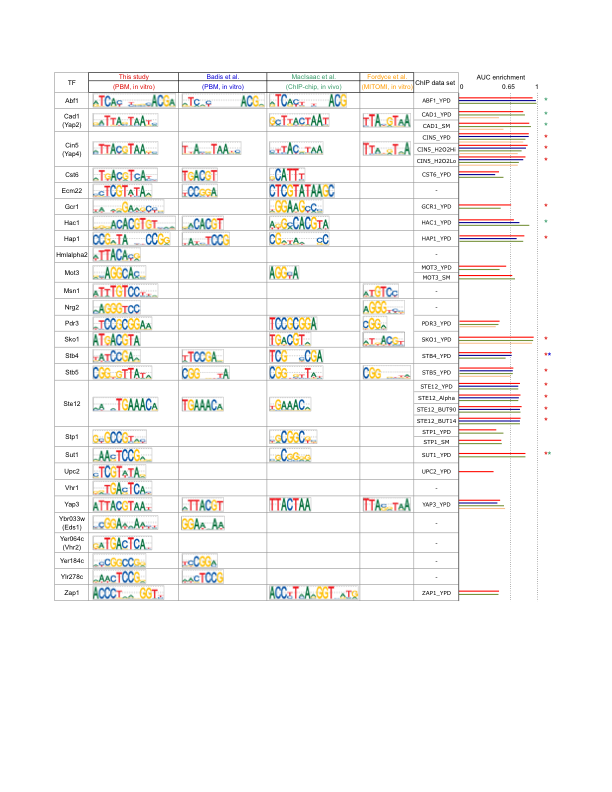


- **Figure S5.** Comparison of motif enrichment in ChIP-chip data for the 27 TF motifs reported in this study versus previously reported PBM-derived (Badis et al. 2008), ChIP-derived (MacIsaac et al. 2006), or MITOMI-derived (Fordyce et al. 2010) motifs for these 27 TFs (where available).


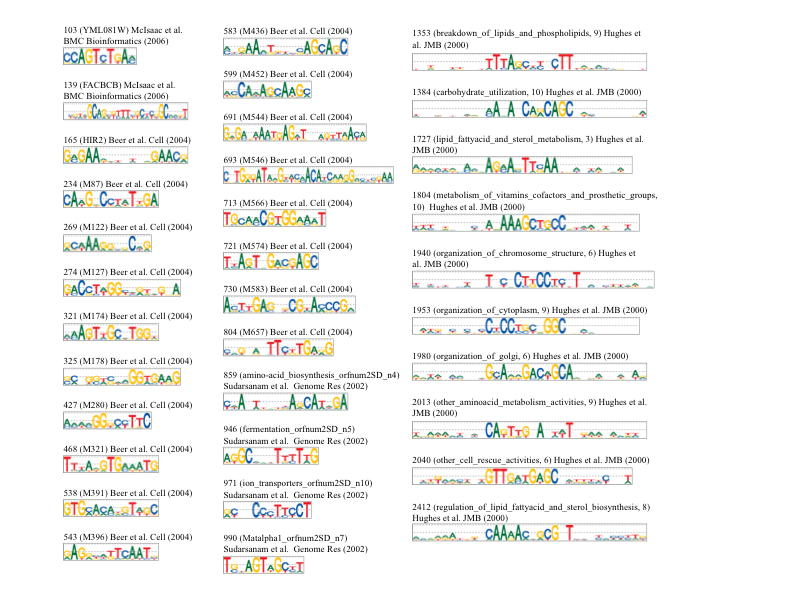


- **Figure S6.** *S. cerevisiae* orphan DNA binding site motifs.


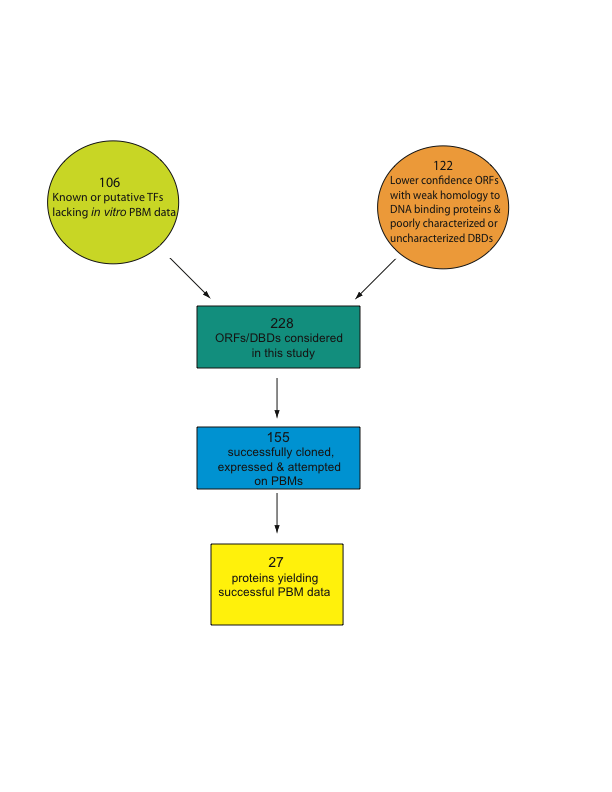


- **Figure S7.** Schema of PBM experimental pipeline and results. A total of 228 ORFs/DBDs were considered in this study. Those lacking *in vitro* PBM data refers to initiation of this study in late 2008 after completion of our prior PBM survey (Zhu et al. 2009) and prior to publication of two more recent *in vitro* surveys (Badis et al. 2008; Fordyce et al. 2010).

1. ^^ Of the 27 motifs derived from PBM data reported in the manuscript, the Hac1 motif is the only one that we did not select for the curated list of motifs (the motif from Badis et al. was chosen instead). We note that both the Badis et al. PBM data and the PBM data reported in this manuscript are of high quality and are in good agreement, but the motif reported by Badis et al. is more enriched in the ChIP-chip dataset Hac1_YPD (AUC=0.7656 compared to 0.6906, both with associated p-value≤0.005). [↑](#footnote-ref-2)
