# Supplementary material for: Curated collection of yeast transcription factor DNA binding specificity data reveals novel structural and gene regulatory insights
Source: Genome Biol. 2011 Dec 21;12(12):R125. doi: 10.1186/gb-2011-12-12-r125 (PMC3334620; doi:10.1186/gb-2011-12-12-r125)
Supplement: Additional file 4 — Table S4. Comparison of high-resolution in vitro DNA binding site motifs for S. cerevisiae TFs. [file gb-2011-12-12-r125-S4.PDF]

Table S4. Comparison of high-resolution *in vitro* DNA binding site motifs for *S. cerevisiae* TFs.

No.	TF	Murphy/Zhu et al. PWM	Badis et al. PWM	Fordyce et al. PWM	Selected PWM
1	Abf1	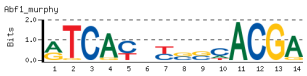	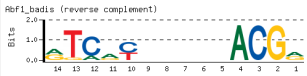		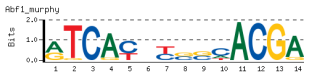
2	Abf2		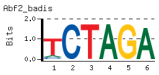		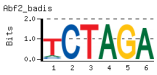
3	Ace2		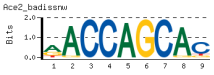	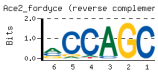	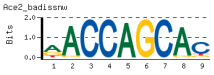
4	Adr1		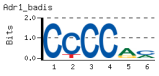		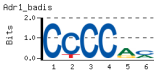
5	Aft1	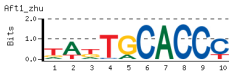		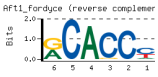	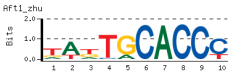
6	Aft2		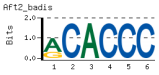	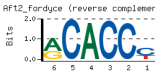	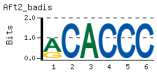
7	Aro80	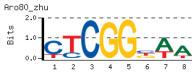			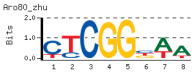
8	Asg1	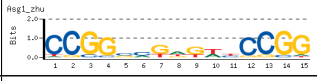	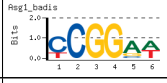		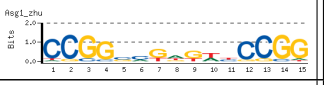
9	Azf1		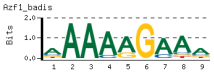		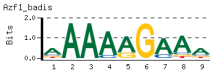
10	Bas1	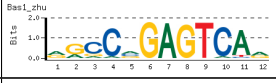		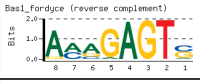	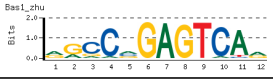
11	Cad1	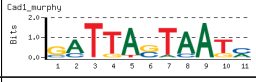		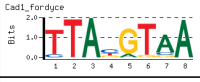	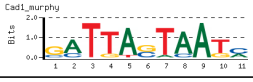
12	Cat8		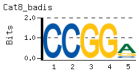		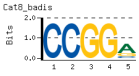
13	Cbf1	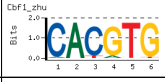	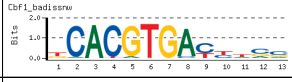	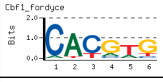	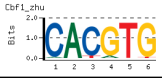
14	Cep3	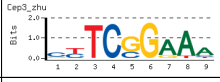	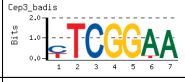		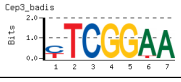
15	Cha4	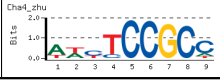	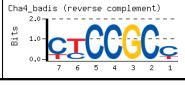		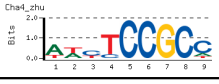

No.	TF	Murphy/Zhu et al. PWM	Badis et al. PWM	Fordyce et al. PWM	Selected PWM
16	Cin5	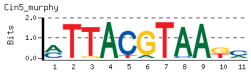	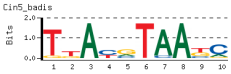	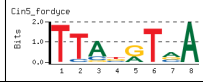	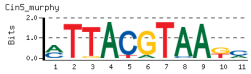
17	Crz1		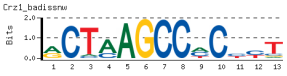		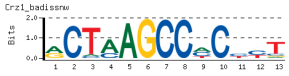
18	Cst6	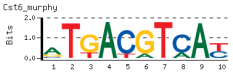	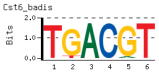		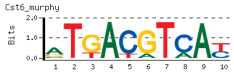
19	Cup9	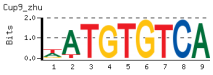	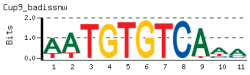	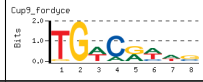	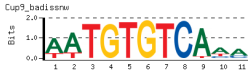
20	Dal80		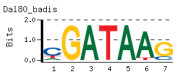	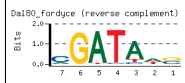	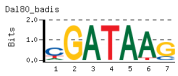
21	Dal82		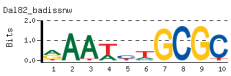		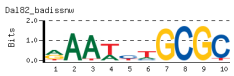
22	Ecm22	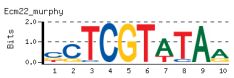	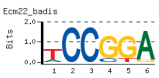		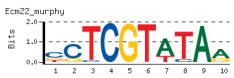
23	Ecm23		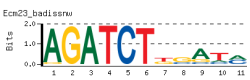		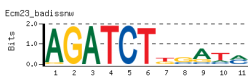
24	Eds1	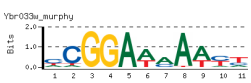	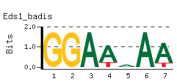		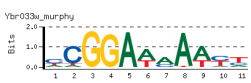
25	Fhl1	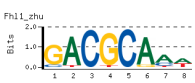	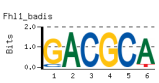		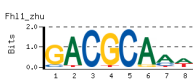
26	Fkh1	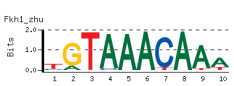			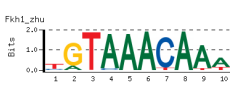
27	Fkh2	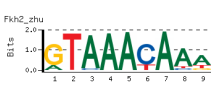	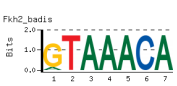		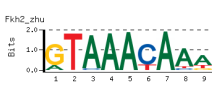
28	Fzf1		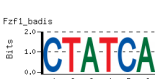		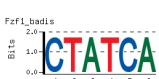
29	Gal4	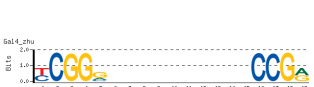			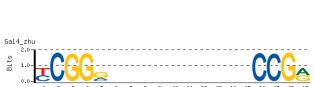
30	Gat1	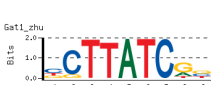	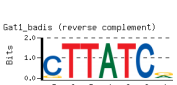	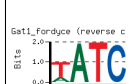	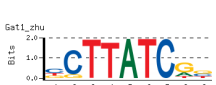
31	Gat3	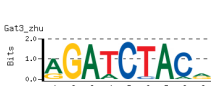	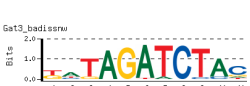		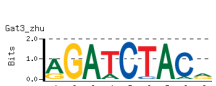

No.	TF	Murphy/Zhu et al. PWM	Badis et al. PWM	Fordyce et al. PWM	Selected PWM
32	Gat4	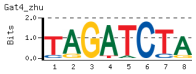	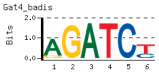		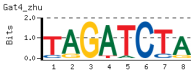
33	Gcn4	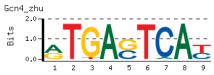		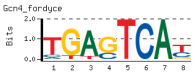	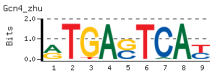
34	Gcr1	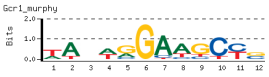			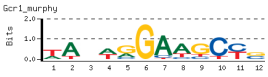
35	Gis1		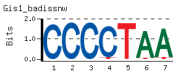		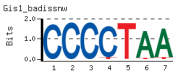
36	Gln3	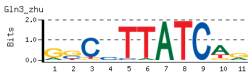	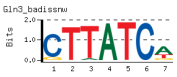		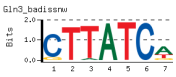
37	Gsm1	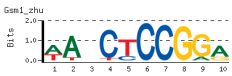	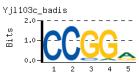		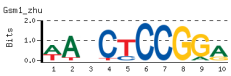
38	Gzf3	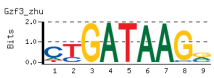	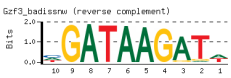		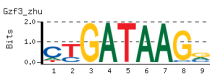
39	Hac1	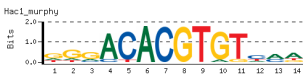	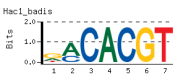		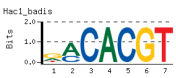
40	Hal9	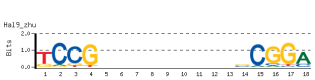	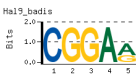		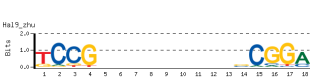
41	Hap1	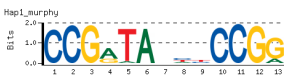	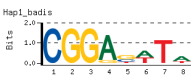		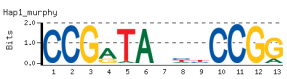
42	Hcm1		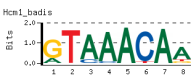		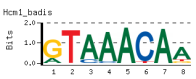
43	Hmlalpha2	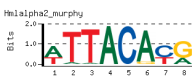			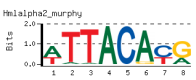
44	Hmra2		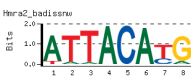		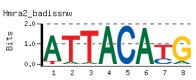
45	Hsf1		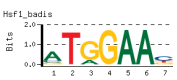		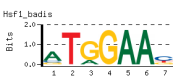
46	Leu3	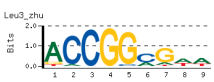			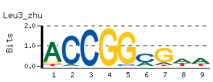
47	Lys14	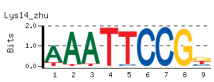	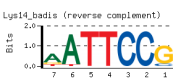		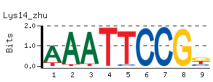

No.	TF	Murphy/Zhu et al. PWM	Badis et al. PWM	Fordyce et al. PWM	Selected PWM
48	Matalpha2	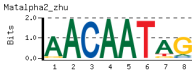		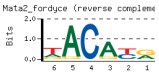	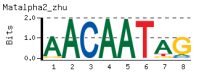
49	Mbp1	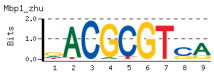	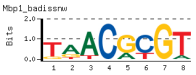		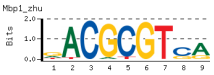
50	Mcm1	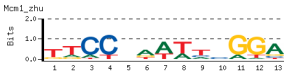		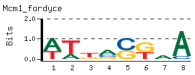	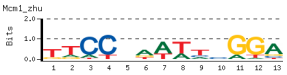
51	Met31		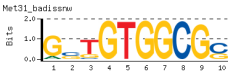	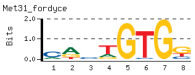	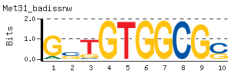
52	Met32	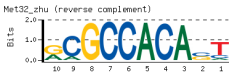	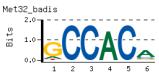	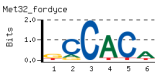	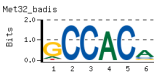
53	Mga1	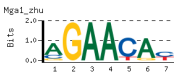			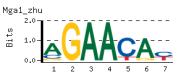
54	Mig1	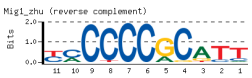	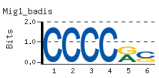		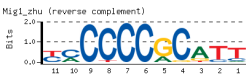
55	Mig2	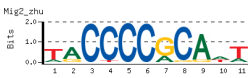	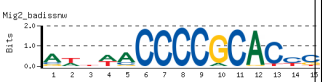		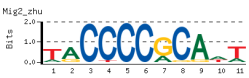
56	Mig3	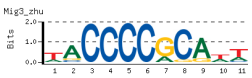	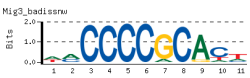		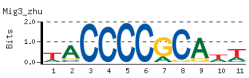
57	Mot3	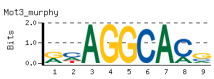			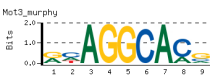
58	Msn1	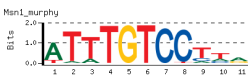		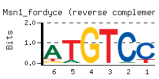	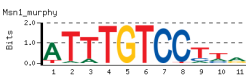
59	Msn2		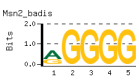	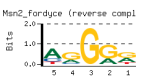	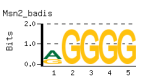
60	Msn4		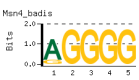		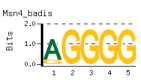
61	Ndt80	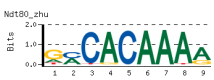			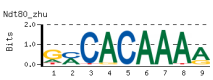
62	Nhp10		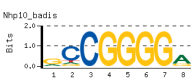		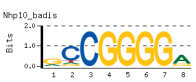
63	Nhp6a	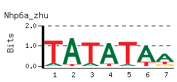			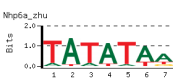

No.	TF	Murphy/Zhu et al. PWM	Badis et al. PWM	Fordyce et al. PWM	Selected PWM
64	Nhp6b	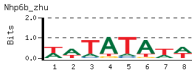			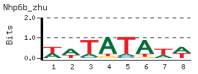
65	Nrg1	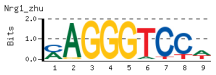			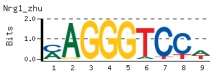
66	Nrg2	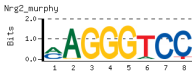		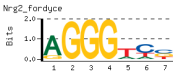	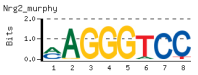
67	Oaf1	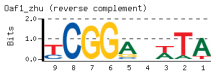	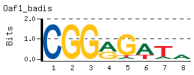		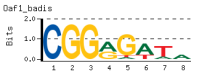
68	Pbf1	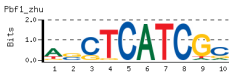	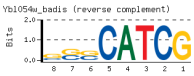		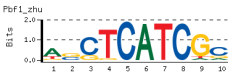
69	Dot6	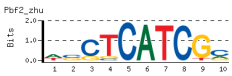	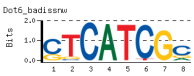		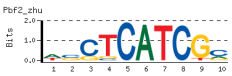
70	Pdr1	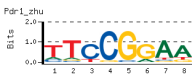	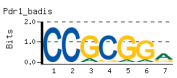		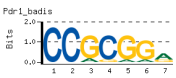
71	Pdr3	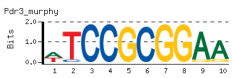		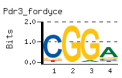	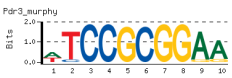
72	Pdr8		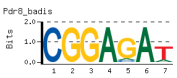		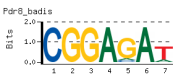
73	Phd1	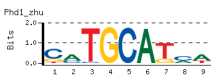	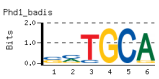		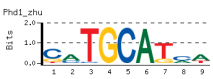
74	Pho2	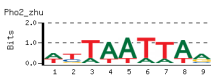	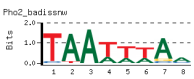		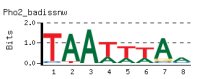
75	Pho4	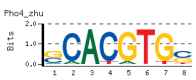	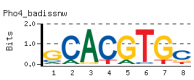	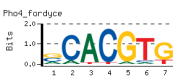	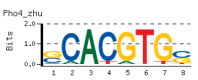
76	Put3	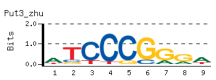	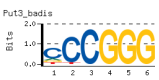		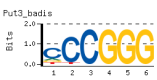
77	Rap1	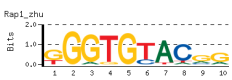			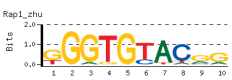
78	Rdr1	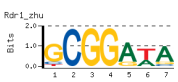	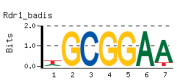		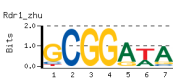
79	Rds1	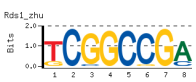	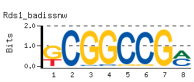		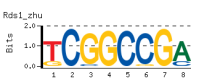

No.	TF	Murphy/Zhu et al. PWM	Badis et al. PWM	Fordyce et al. PWM	Selected PWM
80	Rds2	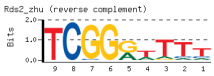	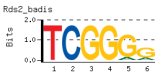		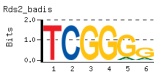
81	Reb1		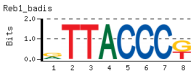	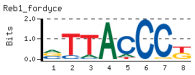	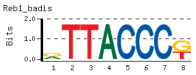
82	Rei1		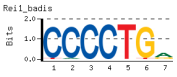		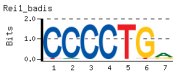
83	Rfx1		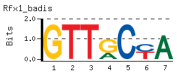		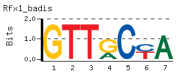
84	Rgm1		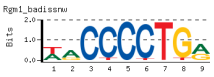		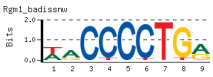
85	Rgt1	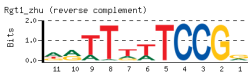	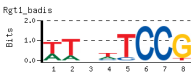		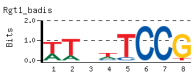
86	Rim101		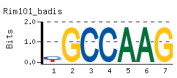		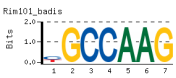
87	Rox1		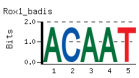	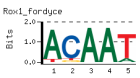	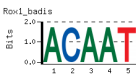
88	Rph1	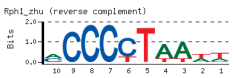	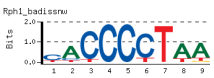		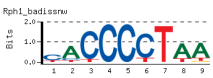
89	Rpn4	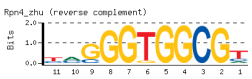	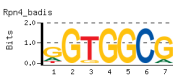	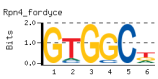	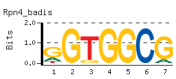
90	Rsc3	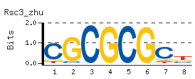	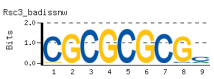		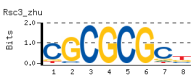
91	Rsc30	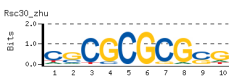	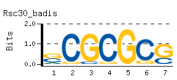		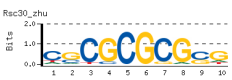
92	Rtg3	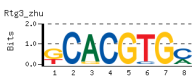			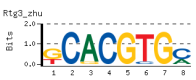
93	Sfl1	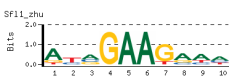			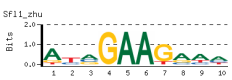
94	Sfp1	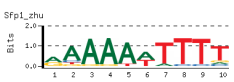			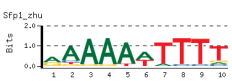
95	Sig1		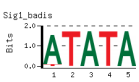		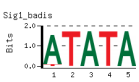

No.	TF	Murphy/Zhu et al. PWM	Badis et al. PWM	Fordyce et al. PWM	Selected PWM
96	Sip4	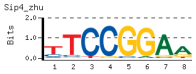	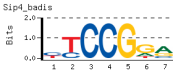		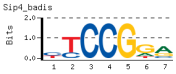
97	Skn7	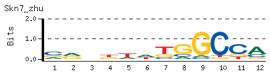	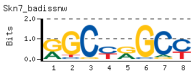		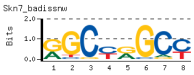
98	Sko1	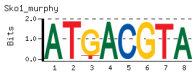		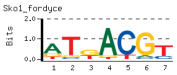	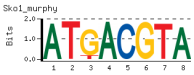
99	Smp1	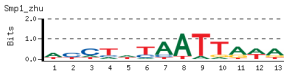			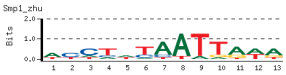
100	Sok2		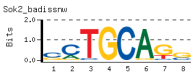		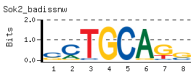
101	Spt15	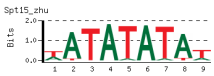			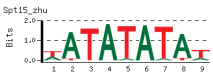
102	Srd1	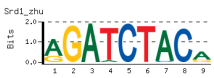	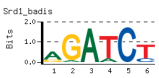		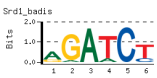
103	Stb3	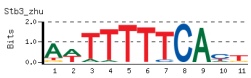			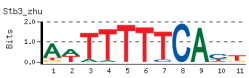
104	Stb4	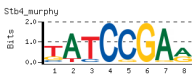	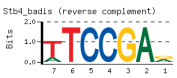		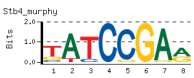
105	Stb5	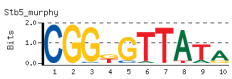	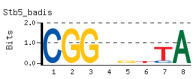	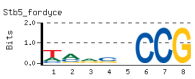	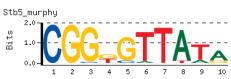
106	Ste12	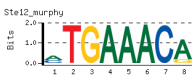	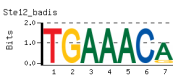		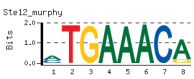
107	Stp1	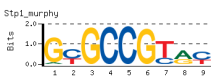			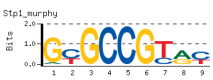
108	Stp2	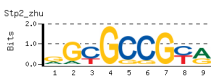			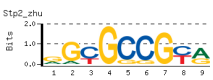
109	Stp3		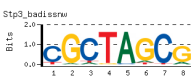		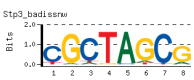
110	Stp4	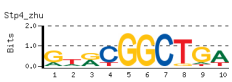	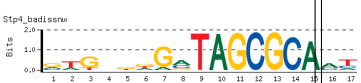		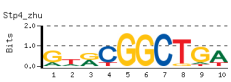
111	Sum1	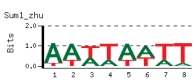	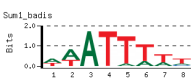		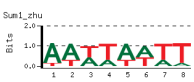

No.	TF	Murphy/Zhu et al. PWM	Badis et al. PWM	Fordyce et al. PWM	Selected PWM
112	Sut1	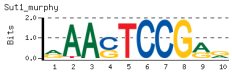			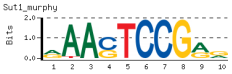
113	Sut2	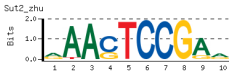			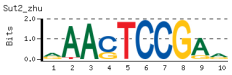
114	Swi4		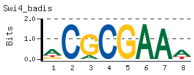		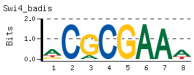
115	Swi5		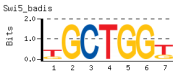		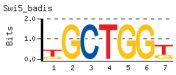
116	Tbf1	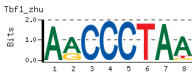	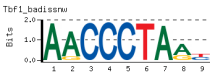		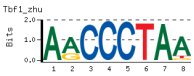
117	Tbs1	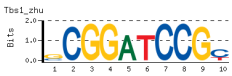	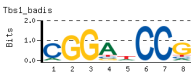		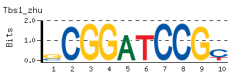
118	Tea1	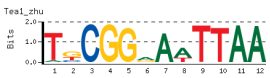	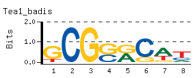		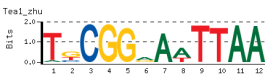
119	Tec1	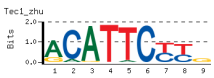	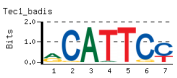		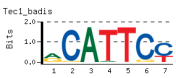
120	Tos8		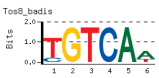		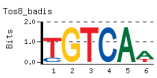
121	Tye7	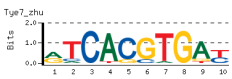	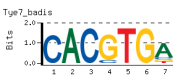		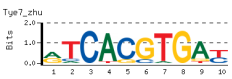
122	Uga3		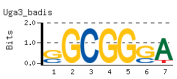		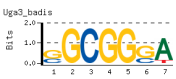
123	Ume6	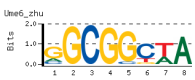	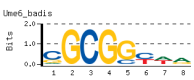		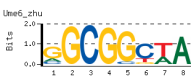
124	Upc2	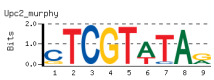			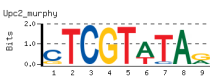
125	Usv1	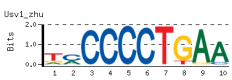	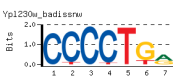		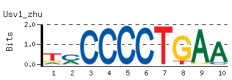
126	Vhr1	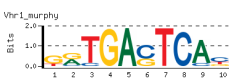			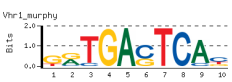
127	Xbp1	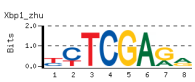	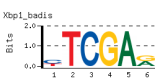		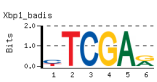

No.	TF	Murphy/Zhu et al. PWM	Badis et al. PWM	Fordyce et al. PWM	Selected PWM
128	Yap1	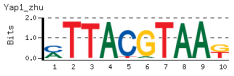		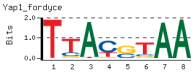	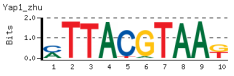
129	Yap3	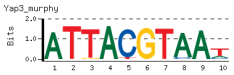	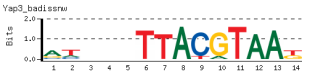	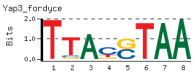	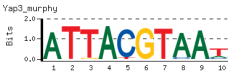
130	Yap6	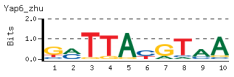			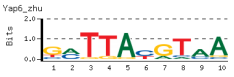
131	Ybr239c	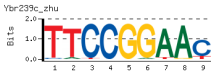	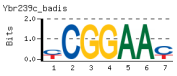		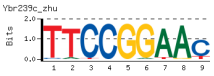
132	Ydr520c	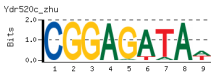	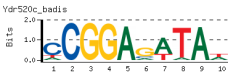		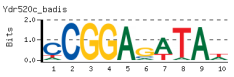
133	Yer064c	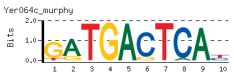			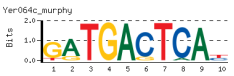
134	Yer130c	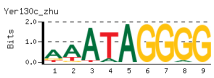	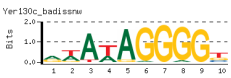		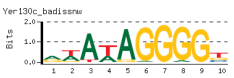
135	Yer184c	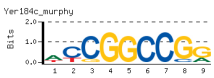	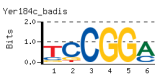		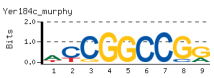
136	Ygr067c	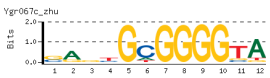	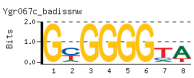		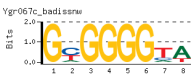
137	Ykl222c	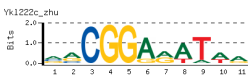	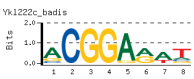		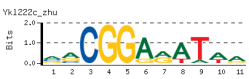
138	Yll054c	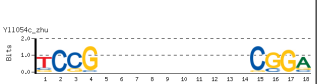	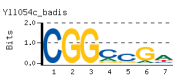		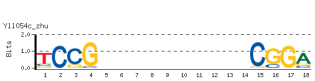
139	Ylr278c	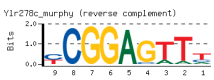	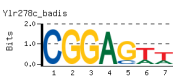		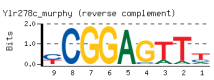
140	Yml081w	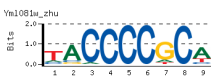	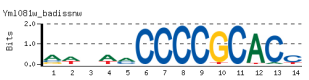		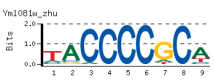
141	Ynr063w	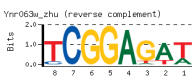	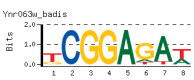		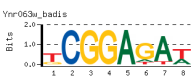
142	Yox1	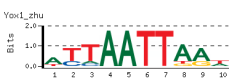	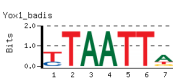		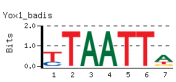
143	Ypr013c	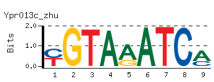	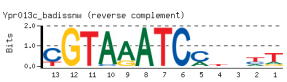		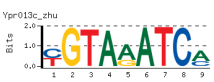

No.	TF	Murphy/Zhu et al. PWM	Badis et al. PWM	Fordyce et al. PWM	Selected PWM
144	Ypr015c	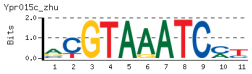			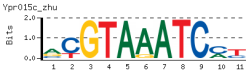
145	Ypr022c		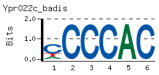		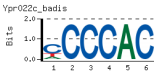
146	Ypr196w	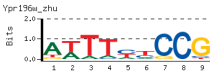	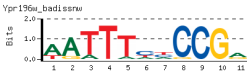		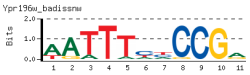
147	Yrm1	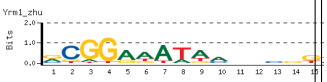	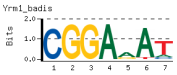		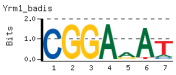
148	Yrr1	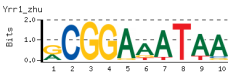	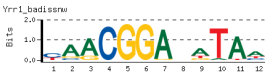		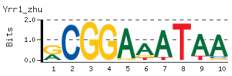
149	Zap1	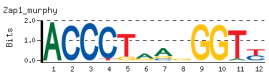			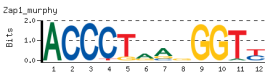
150	Zms1		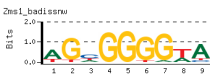		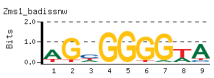
