# Supplementary material for: Curated collection of yeast transcription factor DNA binding specificity data reveals novel structural and gene regulatory insights
Source: Genome Biol. 2011 Dec 21;12(12):R125. doi: 10.1186/gb-2011-12-12-r125 (PMC3334620; doi:10.1186/gb-2011-12-12-r125)
Supplement: Additional file 5 — Table S6. Comparison of in vivo motifs (MacIsaac et al. [20]) and in vitro motifs (selected from this study, Zhu et al. [11], or Badis et al. [10]) for 150 S. cerevisiae TFs. TFs for which the in vivo and in vitro motifs are different are marked in red font. [file gb-2011-12-12-r125-S5.PDF]

Table S6. Comparison of *in vivo* motifs (MacIsaac et al. 2006) and *in vitro* motifs (selected from this study, Zhu et al. 2009, or Badis et al. 2008) for 150 *S. cerevisiae* TFs. TFs for which the *in vivo* and *in vitro* motifs are different are marked in red font.

No.	TF	*In vivo* motif (MacIsaac et al.)	*In vitro* PBM motif (primary)	*In vitro* PBM motif (secondary)
1	Abf1	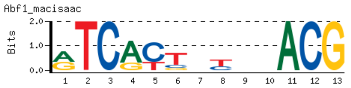	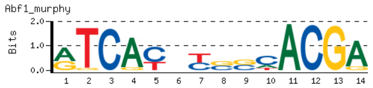	
2	Abf2		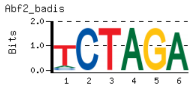	
3	Ace2	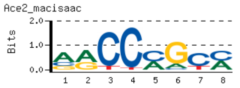	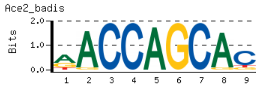	
4	Adr1	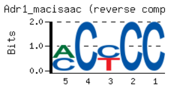	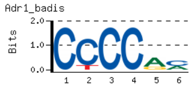	
5	Aft1	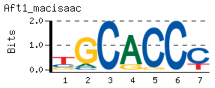	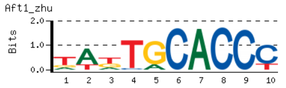	
6	Aft2	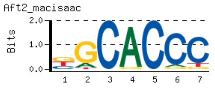	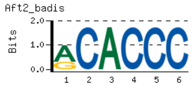	
7	Aro80	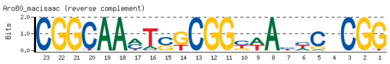	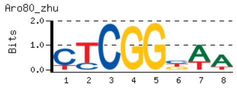	
8	Asg1		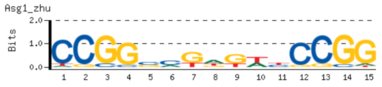	
9	Azf1	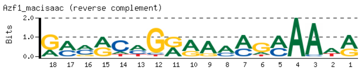	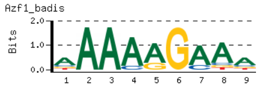	
10	Bas1	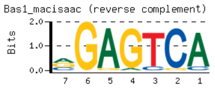	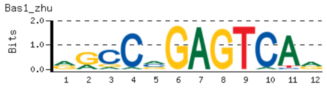	
11	Cad1	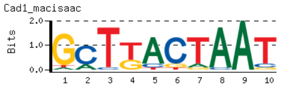	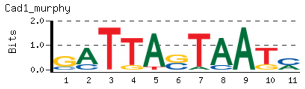	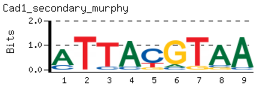
12	Cat8		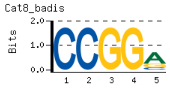	
13	Cbf1	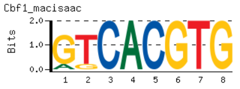	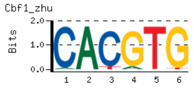	
14	Cep3		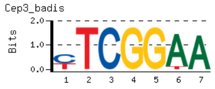	
15	Cha4	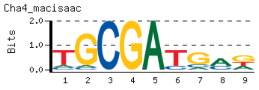	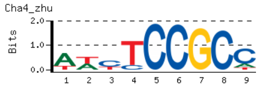	

No.	TF	*In vivo* motif (MacIsaac et al.)	*In vitro* PBM motif (primary)	*In vitro* PBM motif (secondary)
16	Cin5	Cin5_macisaac 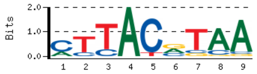	Cin5_murphy 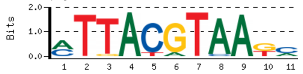	
17	Crz1	Crz1_macisaac (reverse complement) 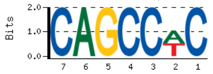	Crz1_badis 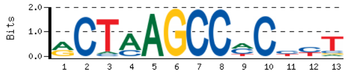	
18	Cst6	Cst6_macisaac 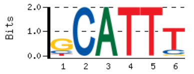	Cst6_murphy 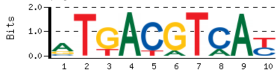	Cst6_secondary_murphy 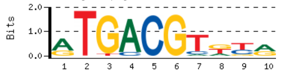
19	Cup9		Cup9_badis 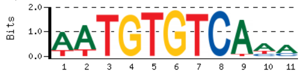	
20	Dal80	Dal80_macisaac 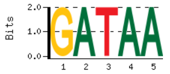	Dal80_badis 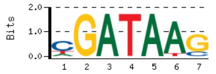	
21	Dal82	Dal82_macisaac 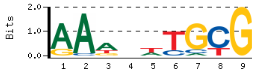	Dal82_badis 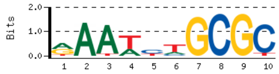	
22	Ecm22	Ecm22_macisaac 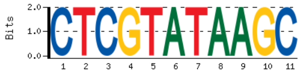	Ecm22_murphy 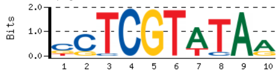	Ecm22_secondary_murphy (reverse comple 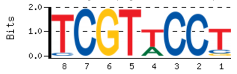
23	Ecm23		Ecm23_badis 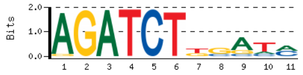	
24	Fhl1	Fhl1_macisaac 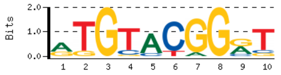	Fhl1_zhu 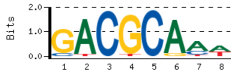	
25	Fkh1	Fkh1_macisaac 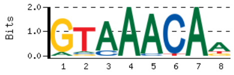	Fkh1_zhu 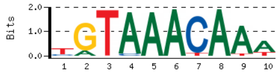	
26	Fkh2	Fkh2_macisaac 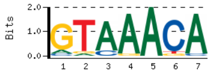	Fkh2_zhu 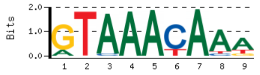	
27	Fzf1		Fzf1_badis 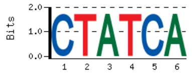	
28	Gal4	Gal4_macisaac 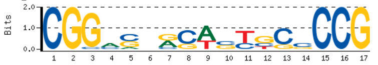	Gal4_zhu 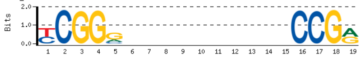	
29	Gat1	Gat1_macisaac 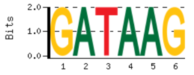	Gat1_zhu (reverse complement) 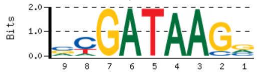	Gat1_secondary_zhu 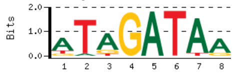
30	Gat3	Gat3_macisaac 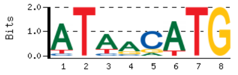	Gat3_zhu 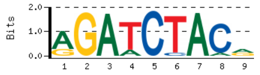	Gat3_secondary_zhu 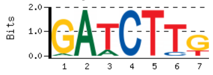
31	Gat4		Gat4_zhu 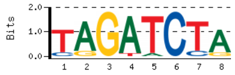	

No.	TF	*In vivo* motif (MacIsaac et al.)	*In vitro* PBM motif (primary)	*In vitro* PBM motif (secondary)
32	Gcn4	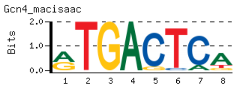	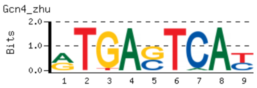	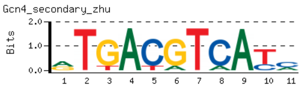
33	Gcr1	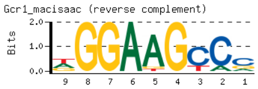	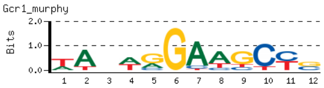	
34	Gis1		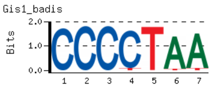	
35	Gln3	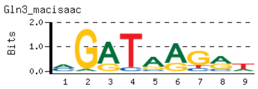	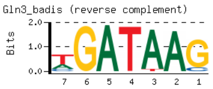	
36	Gsm1		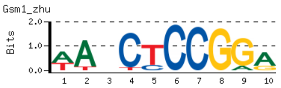	
37	Gzf3	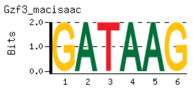	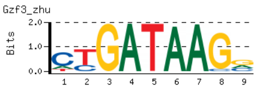	
38	Hac1	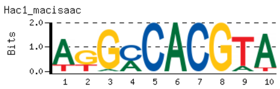	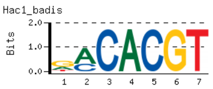	
39	Hal9		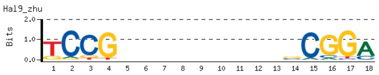	
40	Hap1	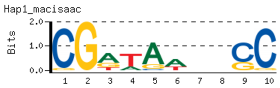	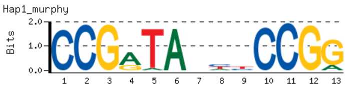	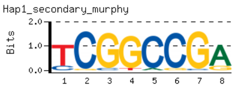
41	Hcm1		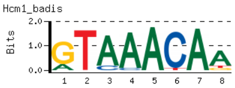	
42	Hmlalpha2		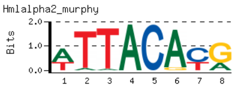	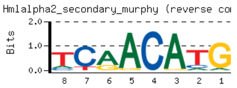
43	Hmra2		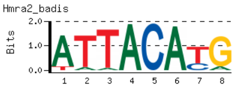	
44	Hsf1	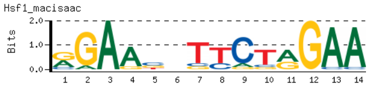	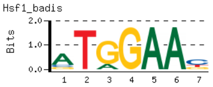	
45	Leu3	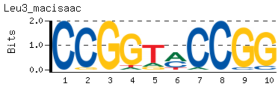	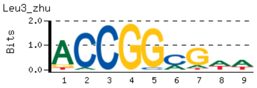	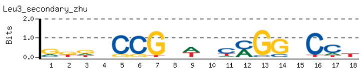
46	Lys14		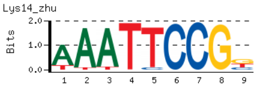	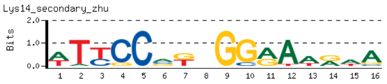
47	Matalpha2		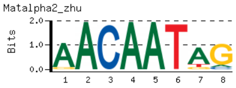	

No.	TF	*In vivo* motif (MacIsaac et al.)	*In vitro* PBM motif (primary)	*In vitro* PBM motif (secondary)
48	Mbp1	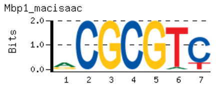	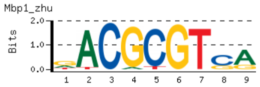	
49	Mcm1	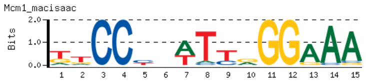	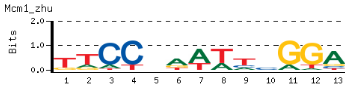	
50	Met31	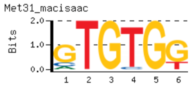	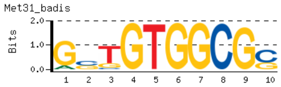	
51	Met32	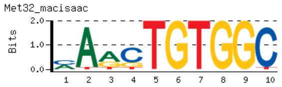	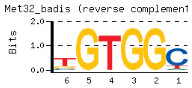	
52	Mga1		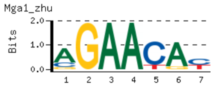	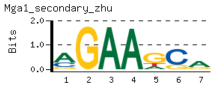
53	Mig1	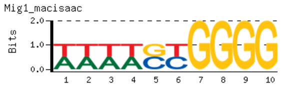	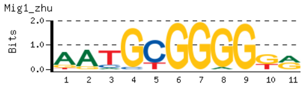	
54	Mig2		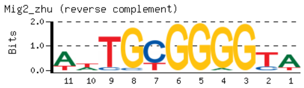	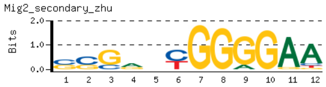
55	Mig3		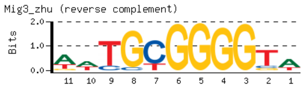	
56	Mot3	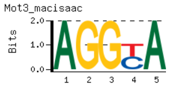	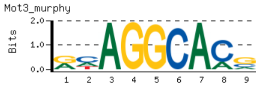	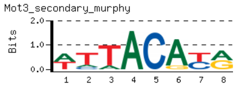
57	Msn1		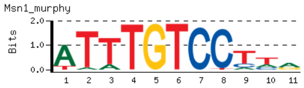	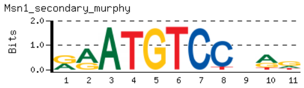
58	Msn2	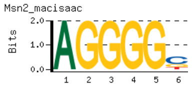	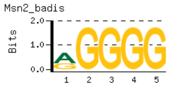	
59	Msn4	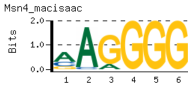	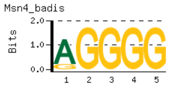	
60	Ndt80		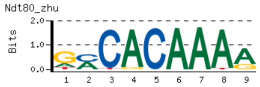	
61	Nhp10		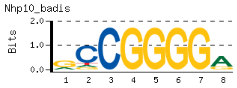	
62	Nhp6a		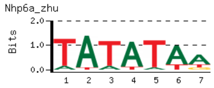	
63	Nhp6b		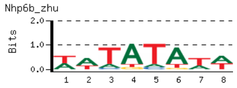	

No.	TF	*In vivo* motif (MacIsaac et al.)	*In vitro* PBM motif (primary)	*In vitro* PBM motif (secondary)
64	Nrg1	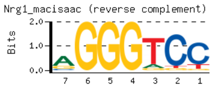	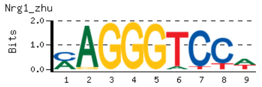	
65	Nrg2		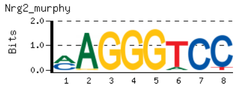	
66	Oaf1		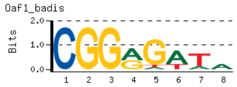	
67	Pbf1		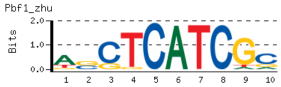	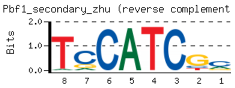
68	Pbf2		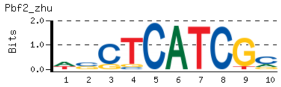	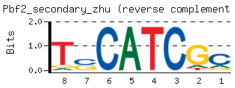
69	Pdr1	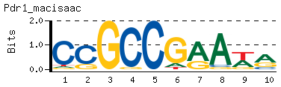	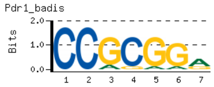	
70	Pdr3	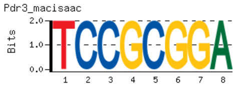	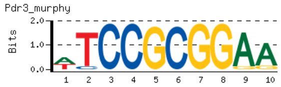	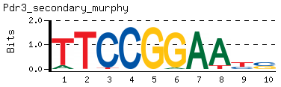
71	Pdr8		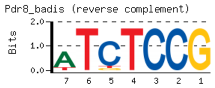	
72	Phd1	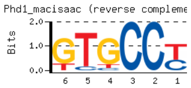	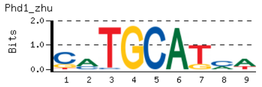	
73	Pho2	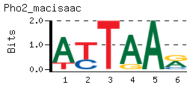	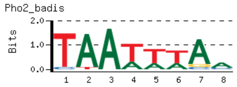	
74	Pho4	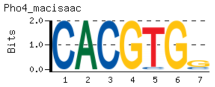	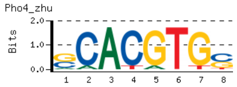	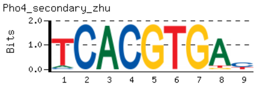
75	Put3	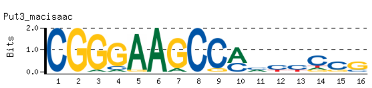	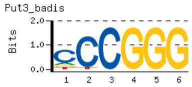	
76	Rap1	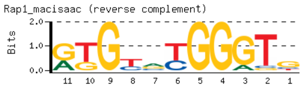	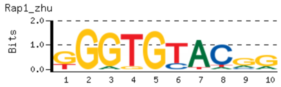	
77	Rdr1		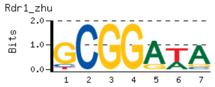	
78	Rds1	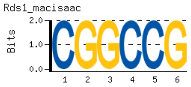	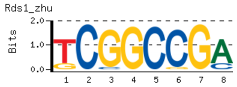	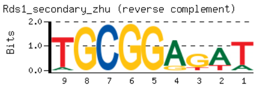
79	Rds2		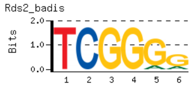	

No.	TF	*In vivo* motif (MacIsaac et al.)	*In vitro* PBM motif (primary)	*In vitro* PBM motif (secondary)
80	Reb1	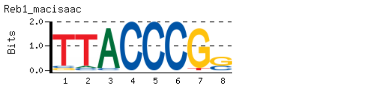	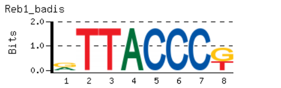	
81	Rei1		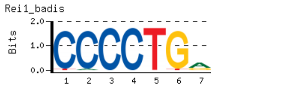	
82	Rfx1	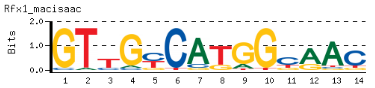	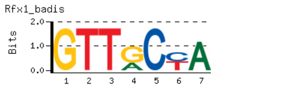	
83	Rgm1		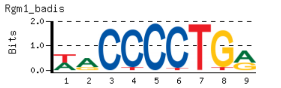	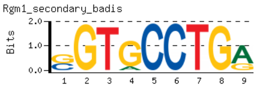
84	Rgt1	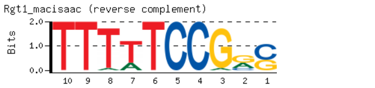	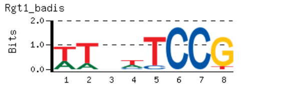	
85	Rim101	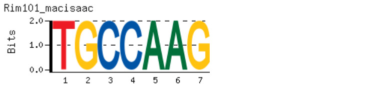	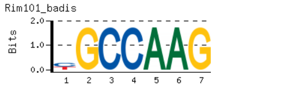	
86	Rox1	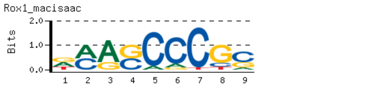	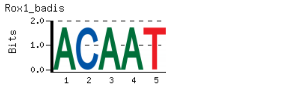	
87	Rph1	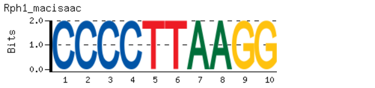	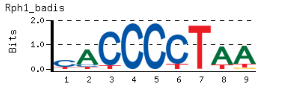	
88	Rpn4	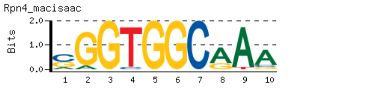	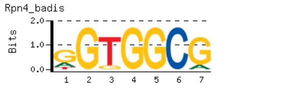	
89	Rsc3		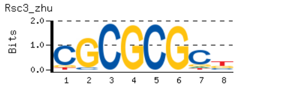	
90	Rsc30		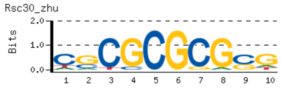	
91	Rtg3	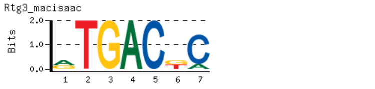	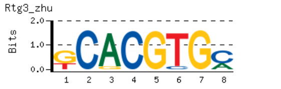	
92	Sfl1	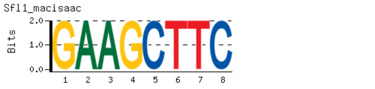	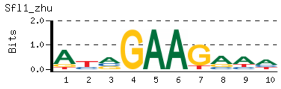	
93	Sfp1	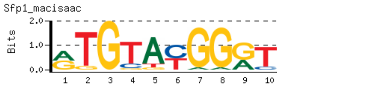	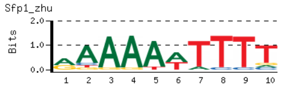	
94	Sig1		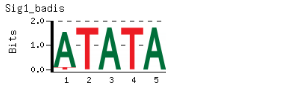	
95	Sip4	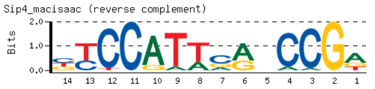	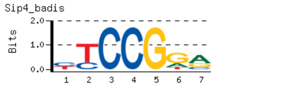	

No.	TF	*In vivo* motif (MacIsaac et al.)	*In vitro* PBM motif (primary)	*In vitro* PBM motif (secondary)
96	Skn7	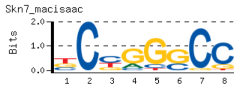	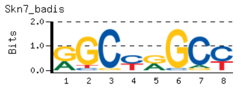	
97	Sko1	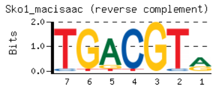	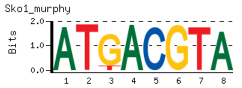	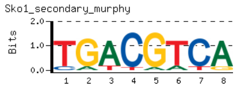
98	Smp1	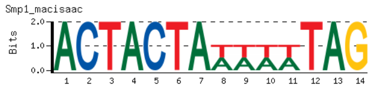	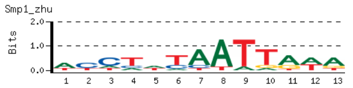	
99	Sok2	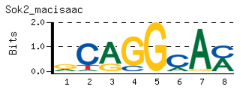	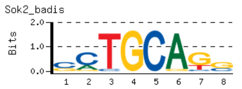	
100	Spt15		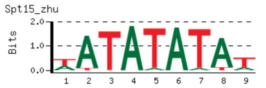	
101	Srd1		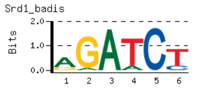	
102	Stb3		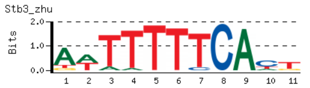	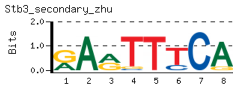
103	Stb4	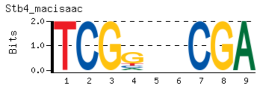	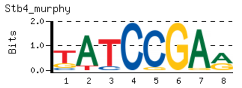	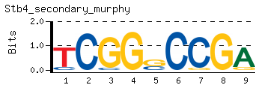
104	Stb5	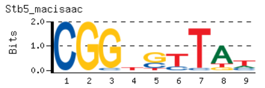	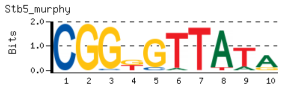	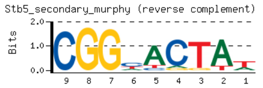
105	Ste12	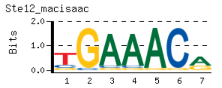	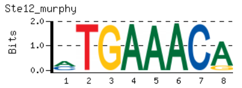	
106	Stp1	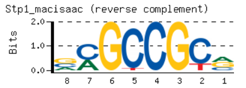	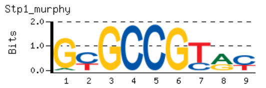	
107	Stp2		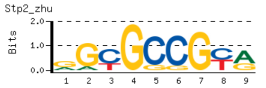	
108	Stp3		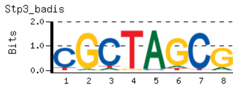	
109	Stp4	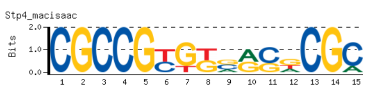	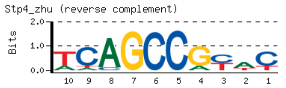	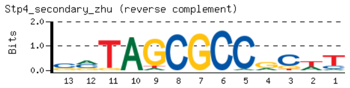
110	Sum1	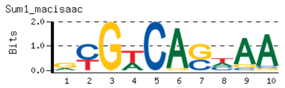	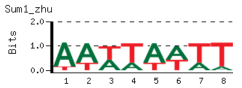	
111	Sut1	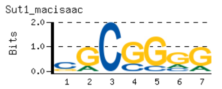	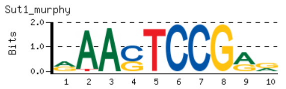	

No.	TF	*In vivo* motif (MacIsaac et al.)	*In vitro* PBM motif (primary)	*In vitro* PBM motif (secondary)
112	Sut2		Sut2_zhu 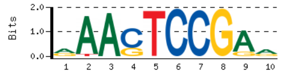	
113	Swi4	Swi4_macisaac 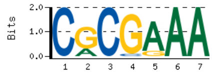	Swi4_badis 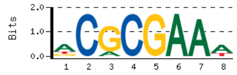	
114	Swi5	Swi5_macisaac 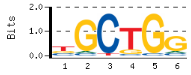	Swi5_badis 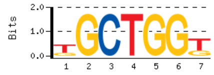	
115	Tbf1		Tbf1_zhu 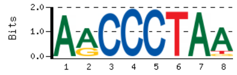	Tbf1_secondary_zhu 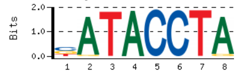
116	Tbs1		Tbs1_zhu 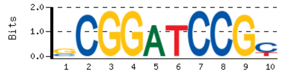	Tbs1_secondary_zhu 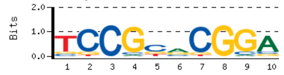
117	Tea1		Tea1_zhu 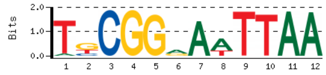	Tea1_secondary_zhu 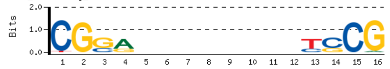
118	Tec1	Tec1_macisaac 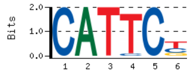	Tec1_badis 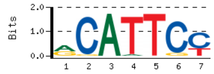	
119	Tos8		Tos8_badis 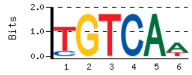	
120	Tye7	Tye7_macisaac 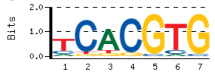	Tye7_zhu 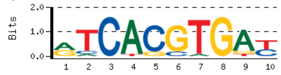	Tye7_secondary_zhu 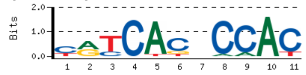
121	Uga3	Uga3_macisaac 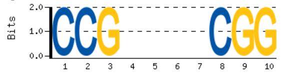	Uga3_badis 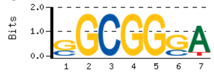	
122	Ume6	Ume6_macisaac (reverse complement) 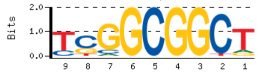	Ume6_zhu 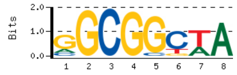	Ume6_secondary_zhu 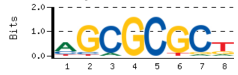
123	Upc2		Upc2_murphy 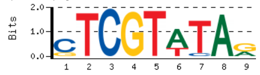	Upc2_secondary_murphy (reverse comp 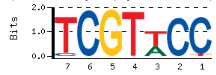
124	Usv1		Usv1_zhu 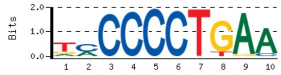	Usv1_secondary_zhu 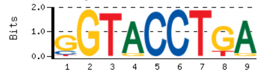
125	Vhr1		Vhr1_murphy 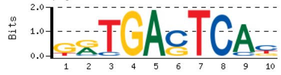	
126	Xbp1	Xbp1_macisaac 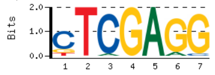	Xbp1_badis 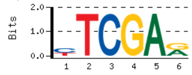	
127	Yap1	Yap1_macisaac 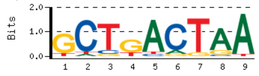	Yap1_zhu 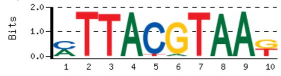	Yap1_secondary_zhu 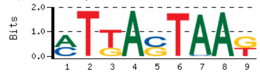

No.	TF	*In vivo* motif (MacIsaac et al.)	*In vitro* PBM motif (primary)	*In vitro* PBM motif (secondary)
128	Yap3	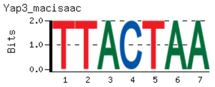	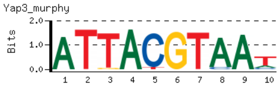	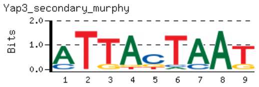
129	Yap6	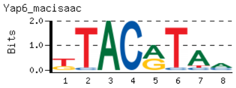	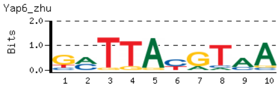	
130	Ybr033w		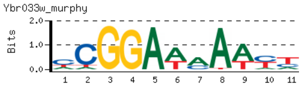	
131	Ybr239c		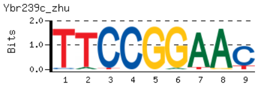	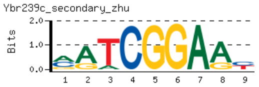
132	Ydr520c	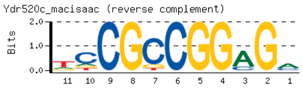	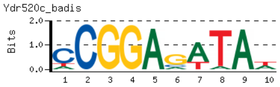	
133	Yer064c		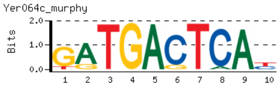	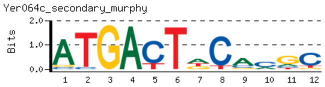
134	Yer130c		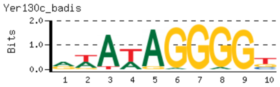	
135	Yer184c		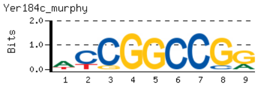	
136	Ygr067c		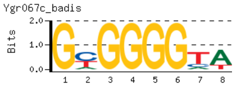	
137	Ykl222c		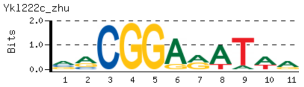	
138	Yll054c		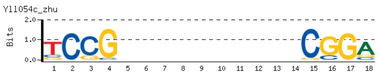	
139	Ylr278c		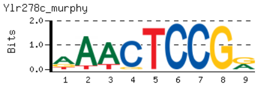	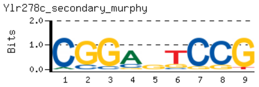
140	Yml081w	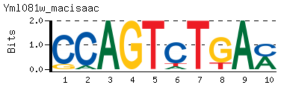	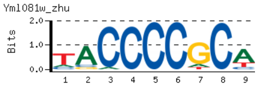	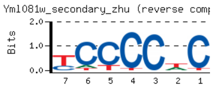
141	Ynr063w		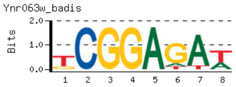	
142	Yox1	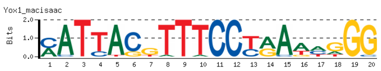	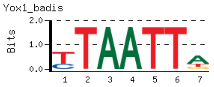	
143	Ypr013c		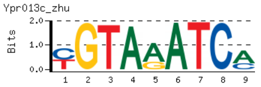	

No.	TF	*In vivo* motif (MacIsaac et al.)	*In vitro* PBM motif (primary)	*In vitro* PBM motif (secondary)
144	Ypr015c		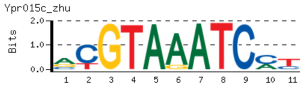	
145	Ypr022c		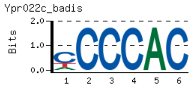	
146	Ypr196w		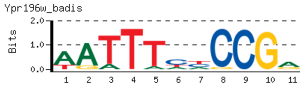	
147	Yrm1		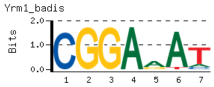	
148	Yrr1	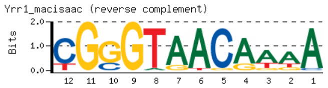	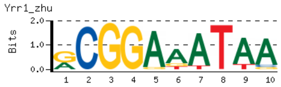	
149	Zap1	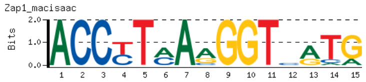	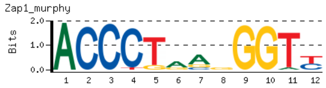	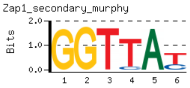
150	Zms1		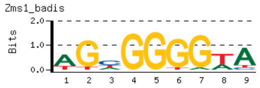	
